# Comparative Genomics of Serial Isolates of *Cryptococcus neoformans* Reveals Gene Associated With Carbon Utilization and Virulence

**DOI:** 10.1534/g3.113.005660

**Published:** 2013-04-01

**Authors:** Kate L. Ormerod, Carl A. Morrow, Eve W. L. Chow, I. Russel Lee, Samantha D. M. Arras, Horst Joachim Schirra, Gary M. Cox, Bettina C. Fries, James A. Fraser

**Affiliations:** * Australian Infectious Diseases Research Centre, University of Queensland, Brisbane, QLD 4072 Australia; † School of Chemistry and Molecular Biosciences, University of Queensland, Brisbane, QLD 4072 Australia; ‡ Centre for Advanced Imaging, University of Queensland, Brisbane, QLD 4072 Australia; § Department of Medicine, Mycology Research Unit, Duke University, Durham, North Carolina 27710; ** Department of Microbiology & Immunology, Albert Einstein College of Medicine, Bronx, New York 10461; †† Department of Medicine, Albert Einstein College of Medicine, Bronx, New York 10461

**Keywords:** microevolution, fungal pathogenesis, cryptococcosis, relapse

## Abstract

The opportunistic fungal pathogen *Cryptococcus neoformans* is a leading cause of mortality among the human immunodeficiency virus/acquired immunodeficiency syndrome population and is known for frequently causing life-threatening relapses. To investigate the potential contribution of in-host microevolution to persistence and relapse, we have analyzed two serial isolates obtained from a patient with acquired immunodeficiency syndrome who suffered an initial and relapse episode of cryptococcal meningoencephalitis. Despite being identical by multilocus sequence typing, the isolates differ phenotypically, exhibiting changes in key virulence factors, nutrient acquisition, metabolic profiles, and the ability to disseminate in an animal model. Whole-genome sequencing uncovered a clonal relationship, with only a few unique differences. Of these, two key changes are expected to explain the phenotypic differences observed in the relapse isolate: loss of a predicted AT-rich interaction domain protein and changes in copy number of the left and right arms of chromosome 12. Gene deletion of the predicted transcriptional regulator produced changes in melanin, capsule, carbon source use, and dissemination in the host, consistent with the phenotype of the relapse isolate. In addition, the deletion mutant displayed altered virulence in the murine model. The observed differences suggest the relapse isolate evolved subsequent to penetration of the central nervous system and may have gained dominance following the administration of antifungal therapy. These data reveal the first molecular insights into how the *Cryptococcus neoformans* genome changes during infection of humans and the manner in which microevolution progresses in this deadly fungal pathogen.

Microevolution enables rapid adaptation to selective pressures, permitting expansion into new niches and persistence in the face of ever-changing harsh environmental conditions. Pathogenic organisms encounter a wide array of challenges during infection of the human host, including oxidative and nitrosative stresses from immune cells, nutrient limitation, and high temperature. Accordingly, genomic changes that confer selective advantages enabling their survival and proliferation have been identified in diverse species. The gastric pathogen *Helicobacter pylori* relies on an elevated mutation rate combined with genetic exchange between strains during mixed infection to generate genetic diversity (Kennemann *et al.* 2011), and chronic *Pseudomonas aeruginosa* infection associated with cystic fibrosis involves selection against virulence factors required for an acute infection (Smith *et al.* 2006; Hoboth *et al.* 2009). Such flexibility is not limited to bacterial pathogens but is also evident in eukaryotic parasites such as *Leishmania* (Ubeda *et al.* 2008) and *Trypanosoma* (Minning *et al.* 2011). This capacity for rapid genomic change renders pathogens difficult to counter as they can develop resistance to drug therapies and evade the host immune response.

Pathogenic fungi also undergo microevolution during infection, most notably in the emergence of drug resistance, which has been described in the three most common systemically infecting fungal genera: *Candida*, *Aspergillus*, and *Cryptococcus* (Selmecki *et al.* 2006; Sionov *et al.* 2010; Mortensen *et al.* 2011). However, beyond antifungal resistance, the adaptive potential of mutation during infection predicts the emergence of fungal strains that are better suited to the hostile host environment. With an estimated global burden of 625,000 deaths per annum (Park *et al.* 2009), *Cryptococcus neoformans* is one such fungal pathogen for which this potential is of significant clinical relevance. A key feature of *C. neoformans* pathogenesis is the ability to establish dormant infections, as immunocompetent hosts are thought to acquire an asymptomatic or subclinical initial infection that remains latent until immune suppression, at which time the infection can emerge (Garcia-Hermoso *et al.* 1999). Patients who appear to have been successfully treated still frequently relapse, with the majority of relapses believed to be a consequence of persistence of the original strain rather than reinfection with a new isolate (Desnos-Ollivier *et al.* 2010). The molecular mechanisms underlying latency and relapse remain unknown and although the patients’ compromised immune system is undoubtedly a contributing factor, changes in the fungal genome may aid the escape from dormancy at the opportune moment.

Previous analyses of serial isolates of *C. neoformans* have established the occurrence of in-host microevolution and attendant phenotypic variation, but the precise molecular events occurring in the genome are poorly understood, although changes in karyotype indicating large-scale genomic rearrangements are frequently observed (Fries *et al.* 1996; Fraser *et al.* 2005). Experimental evolution studies in *Saccharomyces cerevisiae* under nutrient-limiting conditions have demonstrated that large chromosomal mutations occur at a high frequency under such conditions and provide adaptive potential (Dunham *et al.* 2002; Gresham *et al.* 2008). Similarly stressful conditions are likely to be encountered during a pathogens’ progress through the host, providing ideal selective pressure for adaptive mutations to proliferate and spread throughout the population.

Recent studies of *C. neoformans* var. *grubii*, the most clinically prevalent form of this pathogen, have shown that it possesses a remarkably stable genome, with few rearrangements since its divergence from the less common var. *neoformans* an estimated ~20 million years ago (Morrow *et al.* 2012). However, there is evidence that the genome becomes more plastic when placed under the stresses encountered while infecting a human host, with the appearance of remarkably different electrophoretic karyotypes not only between patients, but also between initial and relapse infections of an individual (Fries *et al.* 1996). Clinical isolates readily display profound changes in karyotype (Fries *et al.* 1996), and isolates placed under antifungal stress rapidly accumulate characteristic transient aneuploidies that are lost when the drug pressure is removed (Sionov *et al.* 2010). A nascent theme from ongoing molecular analyses of the systemic fungal pathogens is that although they appear to possess low genetic variability and are genetically stable, once under the selective pressure of a host, microevolution and genetic variation rapidly increases. Elegant studies in the commensal *Candida albicans* have also demonstrated that passage through a host or exposure to various stressors increases the rate of genetic and phenotypic variation (Forche *et al.* 2009, 2011).

Here we describe the first genome sequencing-based investigation into microevolution between serial isolates of *C. neoformans* var. *grubii*. Two isolates were obtained from the cerebrospinal fluid of a woman positive for human immunodeficiency virus suffering from cryptococcal meningoencephalitis, the first during initial diagnosis and the second during a relapse episode 77 days later. Using whole-genome sequencing of both isolates, we describe not only the shared ancestry but also key differences between the two, ranging from single-nucleotide variation to chromosomal aneuploidy. A single mutation in an AT-rich interaction domain (ARID) protein appears responsible for multiple phenotypic differences observed between the isolated strains, and may also contribute to a dissemination defect observed in the later isolate supporting its evolution during infection of the central nervous system (CNS).

## Materials and Methods

### Strains and phenotypic assays

*C. neoformans* var. *grubii* strains used in this study were the type strain H99 (Perfect *et al.* 1980) and serial isolates F0 and F2 and G0, G1, and G2. Fraser Lab H99 is a subculture of the Heitman Lab H99 #4413 (Morrow *et al.* 2012). F0 and F2 were derived from the cerebrospinal fluid (77 days apart) of a patient infected with human immunodeficiency virus who presented with cryptococcal meningoencephalitis to Jacobi Medical Center in the Bronx, New York in 1994. The patient was initially treated with amphotericin B and had also received at least 7 days of fluconazole prior to obtaining the F0 isolate. The patient was discharged and presumably further treated with fluconazole (compliance unclear). She was readmitted 2 months later with relapsed/persistent cryptococcal meningoencephalitis. Amphotericin B and fluconazole treatment was reinstituted. The F2 isolate was cultured from the cerebrospinal fluid 14 days later. Both F0 and F2 have been frozen continuously except during transfer from Albert Einstein College of Medicine. Details of phenotypic assays are given in the Supporting Information, File S1.

### Multilocus sequence typing (MLST) and transposon mapping

Strains were grown in yeast peptone dextrose (YPD) at 30° with shaking for 16 hr. Washed cell pellets were frozen and lyophilized and DNA extracted via the CTAB extraction method as described (Pitkin *et al.* 1996). Sanger sequencing data were analyzed using Sequencher (4.7, Gene Codes Corp, Ann Arbor, MI) and compared with the *Cryptococcus* MLST sequence database (http://cneoformans.mlst.net). A search for known transposons of *C. neoformans* with Basic Local Alignment Search Tool (BLAST) (Altschul *et al.* 1990) was performed to identify matches in the var. *grubii* strain H99 genome. Primers were designed based on these matches to amplify probes from H99 genomic DNA for detection of the DNA transposon Cnirt2 and the retrotransposons Tcn1, Tcn2, Tcn4, and Tcn6. Genomic DNA from each strain was digested, electrophoretically separated, and Southern blot (Southern 2006) hybridization patterns for each selected element were then compared.

### Pulsed-field gel electrophoresis

Preparation of agarose-embedded intact *Cryptococcus* chromosomal DNA was performed as described (Lengeler *et al.* 2000). Chromosomes were separated in 1% pulsed-field certified agarose gels using a CHEF-DRIII pulsed-field gel electrophoresis system (Bio-Rad, Richmond, CA) in 0.5× Tris-borate-ethylenediaminetetraacetic acid running buffer. Running conditions were as follows: Block 1: ramped switch time from 75 sec to 150 sec, 120°, 4 V/cm, 20 hr; Block 2: ramped switch time from 200 sec to 400 sec, 120°, 4 V/cm, 30 hr. All separations were performed at 10−14° using a Bio-Rad cooling module. Chromosomes were stained and visualized with ethidium bromide.

### 1D nuclear magnetic resonance (NMR) spectroscopy

Metabolite extraction, 1D ^1^H NMR spectroscopy, analysis, and metabolite profiling were performed as described (Morrow *et al.* 2012). Strains H99, F0, and F2 were cultured in YNB or YPD plus 2% glucose at 37° for 16 hr for metabolite extraction. The statistical characteristics of the resulting models are listed in Table S3.

### Sequencing and genomic analysis

Using the Illumina Genome Analyzer II, the Australian Genome Research Facility (Brisbane, Australia) generated 75-bp paired end reads (~17 million for F0 and F2, ~8 million for H99) with an average insert size of 200 bp. Reads for H99 were generated to provide a control against which variation in F0 and F2 could be normalized. Details of read trimming, mapping, and assembly are given in File S1. Transposable elements were identified using RepeatMasker (version open-3.3.0) (Smit *et al.* 1996-2010). Structural variation was detected using BreakDancer (Chen *et al.* 2009), CREST (Wang *et al.* 2011), and Dindel (Albers *et al.* 2011). Details are provided in File S1.

### Molecular techniques

Details of molecular techniques are given in File S1 in the supplementary information. *AVC1* was amplified from H99, cloned into pCR2.1-TOPO (Life Technologies), and subsequently subcloned into the neomycin resistance marker containing the plasmid pJAF1 (Fraser *et al.* 2003) and the nourseothricin resistance marker containing pCH233 (McDade and Cox 2001). Biolistic transformation was performed as described (Magditch *et al.* 2012). Details of primers used in this study are contained in Table S4.

### Nematode and murine survival assays

Survival assay of the nematode *Caenorhabditis elegans* was performed according to the protocol of Mylonakis *et al.* (2002) using strain N2 Bristol with *Escherichia coli* OP50 as the food source (Brenner 1974). Assays were performed in triplicate. For murine assays, 7-wk-old female BALB/c mice were infected via nasal inhalation (Cox *et al.* 2000). Ten mice for each strain were inoculated by pipetting a 50-µL drop containing 2.5 × 10^6^ cells (H99, F0, F2) or 5 × 10^5^ (H99, *avc1*Δ, H99 + *AVC1*) onto their nares. Mice were killed when their body weight had reduced by 20% from their preinfection weight. The brain, lungs, spleen, and liver from killed mice were subsequently removed, homogenized, and plated to determine colony-forming units per gram organ weight. For both survival assays, Kaplan-Meier survival curves were plotted using GraphPad Prism 6.0 (GraphPad Software, La Jolla CA) and significance was determined using a log-rank test, whereas organ burden significance was determined using the Student’s *t*-test. *P v*alues of < 0.05 were considered significant. Murine virulence assays were conducted in accordance with the Australian code of practice for the care and use of animals for scientific purposes by the National Health and Medical Research Council and were approved by the Molecular Biosciences Animal Ethics Committee of The University of Queensland (AEC approval number: SCMB/008/11/NHMRC).

## Results

### Serial isolates F0 and F2 share a recent common ancestor

Although relapse of cryptococcal meningoencephalitis is generally caused by a derivative of the original infecting strain (Spitzer *et al.* 1993), recent work has shown that approximately 20% of infections may involve more than one strain, and relapse may sometimes represent a new infection with a new isolate (Desnos-Ollivier *et al.* 2010). We therefore initiated our study of serial isolate strains F0 and F2 by performing MLST analysis with 12 well-established polymorphic loci to determine their relationship (Litvintseva *et al.* 2006). The MLST profiles of F0 and F2 were identical, consistent with isolate F2 being derived from the original F0 strain. This genotype (designated M1) is widespread and in the United States accounted for greater than 25% of isolates in a study by Litvintseva *et al.* (2006); it differs from the well-studied common laboratory strain H99 MLST genotype by only a single nucleotide in one (*LAC1*) of the 12 loci. Although the identical MLST profiles of F0 and F2 are consistent with a serial relationship, the ubiquity of this genotype and similarity to H99 highlights the limited resolution of this technique in a species that is predominantly clonal.

A more powerful epidemiological tool to discriminate between highly related isolates is an analysis of their transposon profile. Approximately 5% of the *C. neoformans* genome consists of transposons (Loftus *et al.* 2005), and the distribution of these has been shown to be highly variable among strains (Jain *et al.* 2005). We used a Southern blotting approach that included the use of probes to five transposons (Cnirt2, Tcn1, Tcn2, Tcn4, and Tcn6) identified by Goodwin & Poulter (2001) to compare their hybridization profile against an array of clinical isolates and each other. Of these transposons, Cnirt2, Tcn1, Tcn2, and Tcn4 showed identical profiles between F0 and F2, profiles that differed from all other isolates tested. The Tcn6 profile was highly variable in all isolates, indicating that this element is likely still functional and highly mobile (Figure S1). Combined with the MLST result, these data show F0 and F2 are clonally related and therefore represent an opportunity to characterize within-host microevolutionary events that occur during human infection.

### F0 and F2 exhibit altered virulence factor expression but respond similarly to multiple stressors

**Figure 1:**
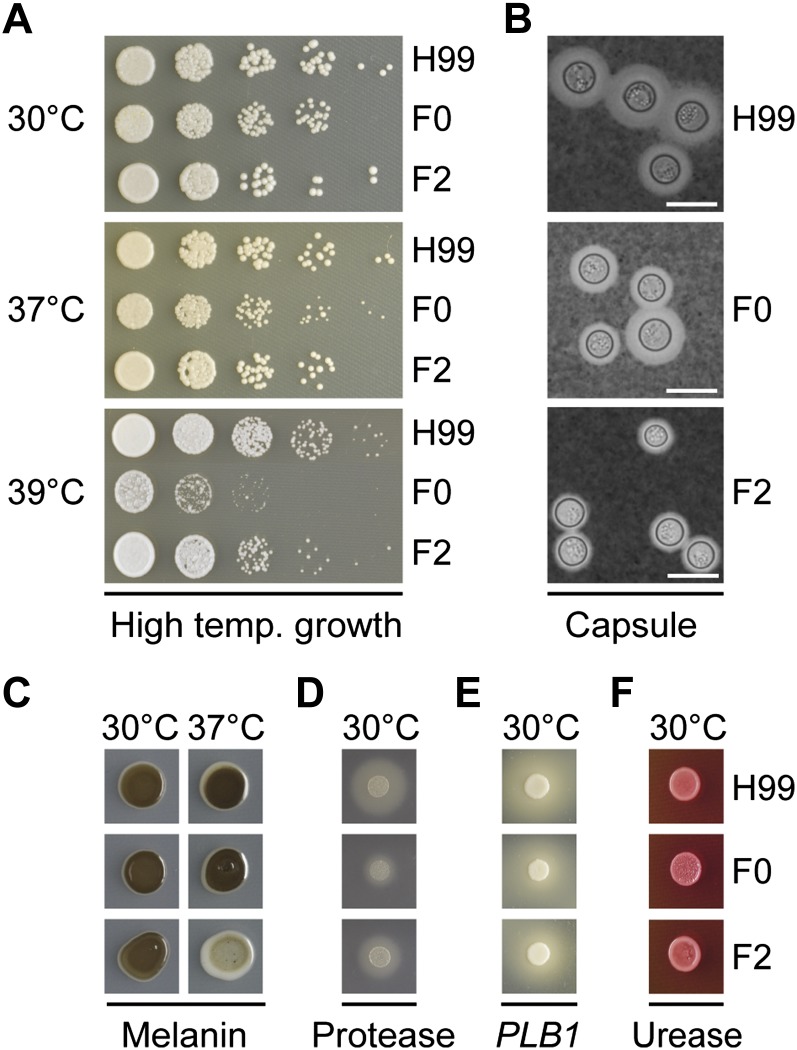
Strains F0 and F2 differ in their production of several virulence factors. (A) Growth assays at 30°, human body temperature of 37°, and febrile body temperature of 39° on YPD. F2 displays similar growth to H99 at 30 and 37° but diminished growth at 39°. (B) India ink staining under light microscopy reveals the capsule; F2 is reduced compared with F0. Scale bar is 10 μM. (C) Melanization was comparable at 30° on l-DOPA−containing media; however, F2 was not melanized at 37°. (D) F0 produces lower levels of extracellular protease when grown on bovine serum albumin agar. (E) and (F) Comparable levels of phospholipase and urease production were observed when strains were grown on egg yolk and Christensen’s agar, respectively.

The theory that *C. neoformans* undergoes microevolutionary change in response to selective pressures encountered in the human host predicts that strains F0 and F2 should exhibit differences in phenotype such as their virulence factor profile and response to stress. Testing revealed growth of the relapse strain F2 exceeded F0 and more closely resembled that of H99, the var. *grubii* type strain, at all temperatures, particularly at 37 and 39°, potentially indicative of selection for optimized growth at human body temperature (Figure 1). Unexpectedly, capsule size was reduced in F2, as was melanization at 37°. Protease production appeared greater in F2 and phospholipase B and urease levels were equivalent between the strains; however, these results are difficult to interpret due to the growth differential between the strains.

Both strains were largely unaffected when exposed to oxidative, nitrosative, ultraviolet rays, membrane and osmotic stress, nutrient limitation, and most cell wall stressors (Figure S2). F0 was severely impaired at pH 9 and greater and F2 at pH 11 (Figure S2). A slight increase in resistance to cell wall stress was observed in F2 grown on caffeine. F2 also displayed increased resistance to fluconazole, with an MIC_90_ (*i.e.*, the minimum inhibitory concentration needed to inhibit the growth of 90% of organisms) of 25 µg/mL, twice that seen for strain F0 (12.5 µg/mL). The acquisition of increased fluconazole resistance is consistent with the patient having received fluconazole as part of their treatment regime.

Serial isolates F0 and F2 therefore meet our criteria for strains that have undergone microevolution: MLST and transposon studies support a common origin, whereas phenotypic analysis reveals emergent differences in key phenotypes associated with proliferation and virulence. This indicates microevolutionary events not observable through MLST and transposon profile analysis have occurred during infection, presenting an ideal opportunity to utilize whole genome sequencing technologies to uncover their causative mutations.

### Substantial genomic variation exists between F0 and F2 and the reference strain H99

**Figure 2:**
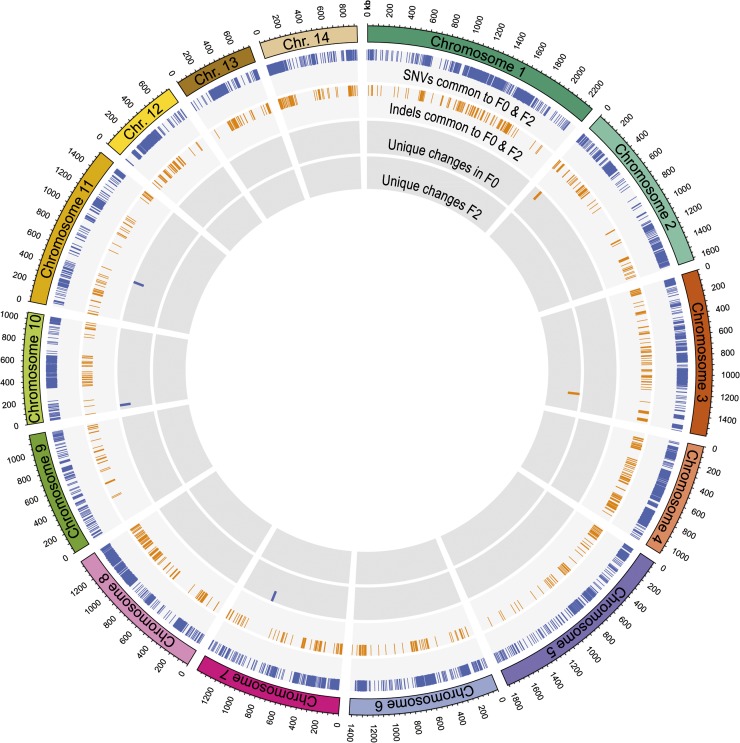
Extensive SNVs and indels are observed in F0 and F2 compared with H99. Circos plot of the 14 *Cryptococcus* chromosomes (outermost rectangles) depicting the >12,000 SNVs and 1200 indels detected in serial isolates F0 and F2 compared with the reference strain H99. Inner rings represent (from outermost): SNVs common to F0 and F2, indels common to F0 and F2, SNVs and indels unique to F0; and SNVs and indels unique to F2.

Illumina Genome Analyzer II reads for each strain were mapped against the Broad Institute’s unpublished 19 Mb draft genome of H99, which comprises 14 chromosomes ranging in size from ~700 kb to ~2.3 Mb. Alignment produced 99.5% coverage of the reference genome at 59×, 58×, and 30× read depth for F0, F2, and H99, respectively. In our MLST analysis we had identified one single-nucleotide variant (SNV) between H99 and our serial clinical isolates F0 and F2; when expanded to the whole genome, 12,119 SNVs were identified in F0 and F2 in comparison with H99, representing 0.06% of the genome (Figure 2). In addition, 1279 shared insertions and deletions less than 50 bp in length (those affecting genes with functional annotation are listed in Table S1A) and 33 larger deletions (Table S1B) were detected in the alignments. Seven additional insertions present in F0 and F2 were subsequently detected using *de novo* assembly of reads that remained unmapped to the reference sequence (Table S1B). In short, the strains differ considerably from the control strain H99 despite their almost identical MLST profile.

### Unique differences exist between F0 and F2

Of the 12,000 differences we detected in F0 and F2 in comparison with the reference strain H99, almost all were common to both isolates. F0 contains just three unique SNVs, one unique indel and a large inversion affecting the first ~210 kb on the left arm of chromosome 3, whereas F2 possesses one unique dinucleotide substitution (Table 1). Of the unique SNVs and indels in F0, the three SNVs are predicted to be silent mutations, whereas the indel alters the coding sequence of DNA polymerase γ (CNAG_06769). This creates a deletion of serine 817, a residue conserved in *Cryptococcus* species, but within a large, 200-residue insertion found only in basidiomycete genomes. Polymerase γ has been shown to be essential in the maintenance of the mitochondrial genome in both *S. cerevisiae* (Foury 1989) and *Schizosaccharomyces pombe* (Chu *et al.* 2007). As we estimated the number of mitochondrial genomes per cell to be similar between F0 and F2 (11 and 9, respectively, based on read coverage), there is likely no phenotypic effect from this mutation. The unique inversion in F0 affects the first ~210 kb on the left arm of chromosome 3 and affects two genes: a small oligopeptide transporter (CNAG_03013) and a short hypothetical protein (CNAG_03012) likely to be an incomplete annotation. A BLAST search identifies the transporter as part of the OPT oligopeptide transporter superfamily. The F2 relapse genome contains one unique change, a substitution of a TA dinucleotide with a G within an exon of a hypothetical protein (CNAG_02579) on chromosome 3; the ensuing frameshift results in a predicted truncation of the protein from 1543 to 130 residues. The gene appears to be unique to *Cryptococcus*, with high identity to homologs in *C. neoformans* var. *neoformans* and *C. gattii* but no other species. The predicted protein contains an ARID found in proteins associated with transcriptional regulation (Kortschak *et al.* 2000), which is lost after truncation of the protein. The presence of genetic variations in F0 absent from F2 suggests the relationship between the isolates is not a simple chronological one and instead likely represents a clonal expansion of an original infecting isolate to form multiple independent lineages, each having evolved concurrently during infection.

**Table 1:** Unique SNVs and indels in F0 and F2

Strain	Chr	H99	Change	Gene function	Effect of mutation
**F0**	2	CTT	—	DNA polymerase γ	Deletion of S817 (of 1,433)
	7	C	A	Conserved hypothetical protein (280 bp upstream)	
	10	T	A	Flavin-containing monooxygenase (30 bp downstream)	
	11	G	C	Dynamin	Synonymous mutation
**F2**	3	AT	G	Conserved hypothetical protein	Q39R, truncation at 130 (of 1,543)

### Both F0 and F2 exhibit aneuploidies of chromosome 12

**Figure 3:**
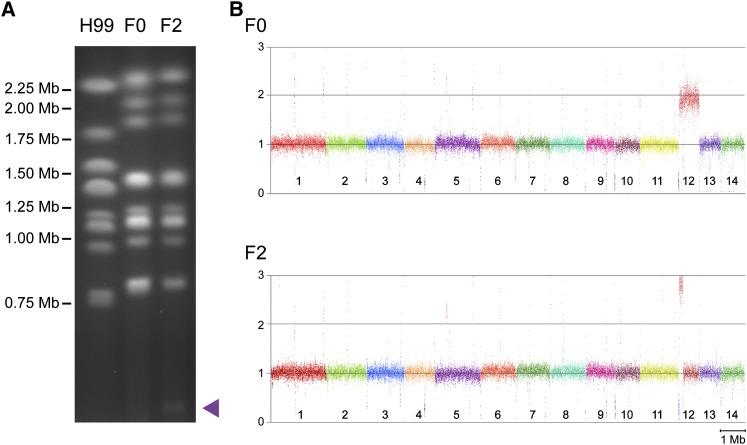
Karyotypic analysis reveals a minichromosome in F2 associated with aneuploidy observed via read depth analysis. (A) Pulsed-field gel electrophoresis of reference strain H99 and serial isolates F0 and F2 reveals a minichromosome of approximately 300 kb. (B) Read depth analysis of serial isolates F0 and F2 in comparison with H99 indicates a duplication of chromosome 12 in F0 and a triplication of the left arm of chromosome 12 in F2.

Careful inspection of the karyotype of strain F2 reveals a minichromosome of approximately 300 kb, only faintly visible in high quality pulsed-field gels (Figure 3A). Evaluation of read depth revealed a triplication of the left arm of chromosome 12 in F2 (Figure 3B). The size of the minichromosome suggested it may represent an isochromosome comprising both the extra copies of the 140 kb left arm of chromosome 12 (12L). Subsequent Southern hybridizations using targeted probes confirmed the minichromosome contained 12L, and digests of intact whole chromosomes on pulsed-field gels yielded bands that were larger than would be expected if each copy of 12L were to be maintained individually (results not shown). Sequence read depth analysis also uncovered an aneuploidy of chromosome 12 in strain F0, which possesses two complete copies of the chromosome. Via parsimony, this suggests that the duplicate copy of the right arm of chromosome 12 was lost during the infection process and the left arm was then triplicated, potentially indicating a selective advantage associated with multiple copies of this genomic region.

Chromosome 12 contains 270 predicted genes, 3.9% of the total genes currently annotated in the H99 genome; 56 of these are contained on the left arm (Table S2). We canvassed the genes on this arm for those that may be potentially advantageous during infection. There are four dehydrogenases present, including three aldehyde and aryl-alcohol dehydrogenases potentially involved in oxidative stress resistance. Interestingly, both genes required for asparagine catabolism are present, asparaginase (CNAG_06008) and aspartate transaminase (CNAG_06026). Asparagine is a preferred nitrogen source of *Cryptococcus* and produces robust melanization (Lee *et al.* 2011). However, our initial assays revealed F2 was unable to produce significant amounts of melanin on l-DOPA media containing asparagine as the nitrogen source at 37°. We extended our analysis of melanization to other nitrogen sources to discern any potential gene dosage effects from the aneuploidies. Melanin production remained low in F2 at 37° on all nitrogen sources tested (Figure S3), and this defect extended to 30° on most other nitrogen sources, although the extent of inhibition was not as drastic.

**Figure 4:**
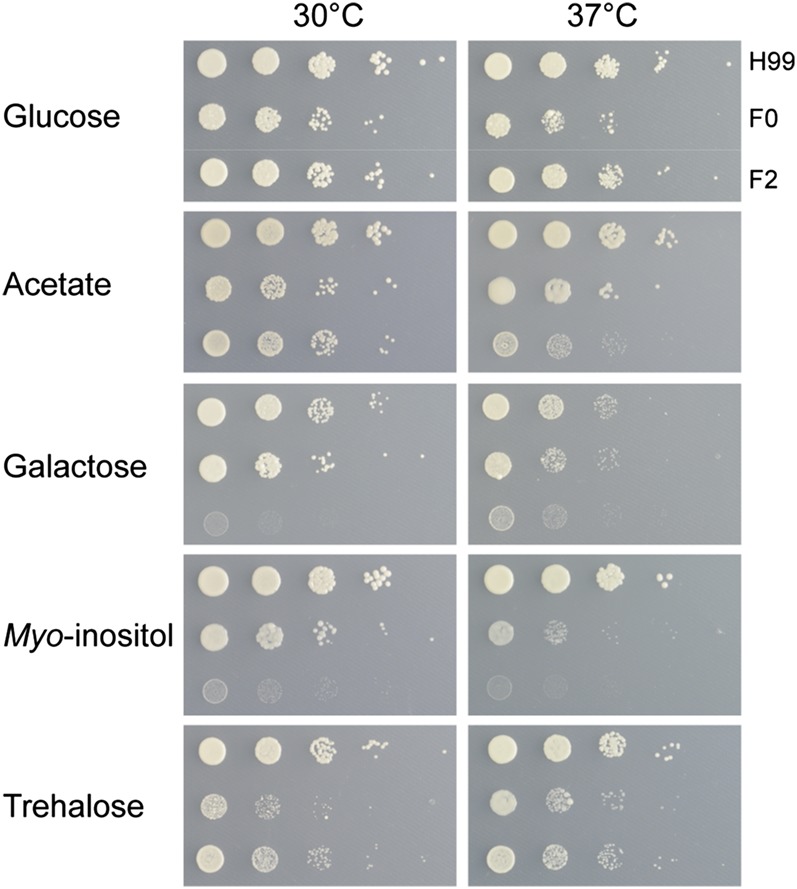
Strains F0 and F2 exhibit different growth on alternate carbon sources. Growth assays on minimal media supplemented with various carbon sources. F0 displays diminished growth on glucose and trehalose at 30 and 37°. F2 displays poor growth on galactose and *myo*-inositol at both temperatures, and acetate at 37°.

We next examined genes on the right arm of chromosome 12 for any that could potentially contribute to the phenotypic differences between the two strains. We first noted the presence of the virulence factor gene phospholipase B (*PLB1*, CNAG_06085); however, increased copy number of this gene in F0 did not yield increased levels of secreted phospholipase B as determined by egg yolk agar assay (Figure 1E). Also present on this arm is the galactose gene cluster, consisting of UDP-glucose epimerase 2 (*GAL10*), galactokinase (*GAL1*), and galactose-1-phosphate uridylyltransferase (*GAL7*). Unlike the clusters of *Saccharomyces*, *Candida*, and *Schizosaccharomyces*, the *Cryptococcus* cluster also includes a putative trehalose permease (Slot and Rokas 2010). We subsequently assayed growth of F0 and F2 on galactose and trehalose and found F2, which in previous assays showed stronger growth, was severely impaired on galactose at both 30 and 37°, whereas both strains were inhibited on trehalose (Figure 4). This finding raised the possibility that the growth differential seen between F0 and F2 was sensitive to the carbon source provided. Growth assays on a range of other sugar and nonsugar carbon sources revealed F2 was also diminished on lactose and maltose, while the apparently weaker strain F0 outperformed F2 on most non-sugar carbon sources tested, except ethanol (Figure S4). Growth on combinations of glucose plus a variety of nonsugar carbon sources resembled that of glucose alone, suggesting the phenotype is dominant (data not shown).

### F0 and F2 exhibit differences in metabolic profiles

**Figure 5:**
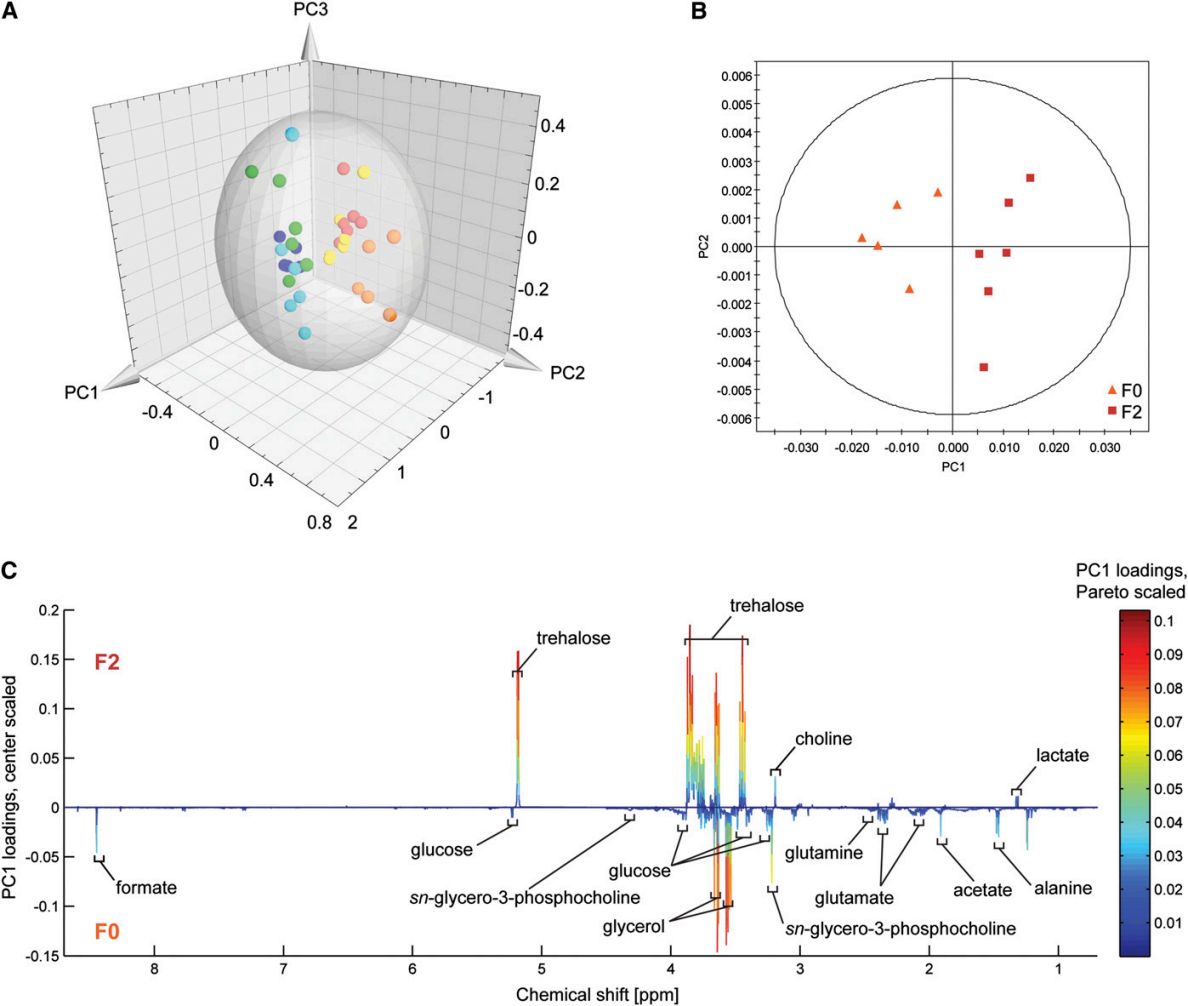
Strains F0 and F2 exhibit different metabolic profiles. (A) Three-dimensional scores plot of the PCA analysis of F0, F2, and H99 under two different growth conditions. All six metabolic types can be distinguished. Red: F2 in YNB; orange: F0 in YNB; yellow: H99 in YNB; green: F2 in YPD; cyan: F0 in YPD; blue: H99 in YPD. Hotelling’s 95% confidence range of the PCA model is indicated by the ellipsoid. (B) PCA scores plot of principal components 1 and 2 for F0 (orange triangles) and F2 (red squares) in YNB. Distance between the points is an indicator of similarity between the samples. (C) Corresponding bivariate loadings line plot. The line plot displays on the loadings coefficient axis the (center scaled) correlation coefficients that relate individual integral regions of the NMR spectrum to the PC1 axis of the scores plot. Individual peaks correspond to peaks in the 1D NMR spectra; peaks that are positive indicate metabolites significantly increased in F2 whereas negative peaks are increased in F0. The overlaid heat map relates the relative contribution of the peak to the scores plot when using Pareto scaling instead of center scaling.

The observed differences in nutrient acquisition prompted an investigation of the metabolic profiles of F0 and F2. We performed 1D proton NMR spectroscopy on metabolites extracted after growth at 37° in rich or minimal media containing the most easily assimilated carbon source glucose. Correlation of spectral peaks with existing metabolite databases and chemical shift standards revealed primarily sugars and amino acids were present, in particular glucose and trehalose, as well as other metabolites such as succinate, acetate, and glycerol. Principal components analysis (PCA) showed systematic differences between all three strains in both growth media (Figure 5A) with the metabolic fingerprint of F2 being more similar to H99 than F0, in agreement with the growth pattern observed in other glucose based assays. Pairwise comparisons between F0 and F2 in minimal media revealed clear separation (Figure 5B), with strain F2 associated with greater levels of trehalose, choline, and lactate and decreased levels of glucose, glycerol, glutamate, glutamine, formate, acetate, alanine, and glycerophosphocholine (Figure 5C). These differences in metabolite profiles were much smaller in rich media than in minimal media, with isolates not dividing into separate clusters and the corresponding model having only marginal predictive power, indicating compromised metabolic pathways may be present in F0 which could result in a greater sensitivity to environmental conditions (Figure S5, A and B, and Table S3). In addition, systematic differences were observed for each individual isolate when profiles were compared between rich and minimal media (Figure S5, C-F).

The metabolites observed are in agreement with those identified in previous *C. neoformans* metabolomics studies, which have reported dominance of trehalose and lipids (Bubb *et al.* 1999; Himmelreich *et al.* 2001). Trehalose is also associated with capacity to grow at 37° (Petzold *et al.* 2006) and increased levels of trehalose have been associated with heat tolerant mutants of *Cryptococcus* (Morrow *et al.* 2012), in agreement with the increased capacity of F2 for growth at this temperature. The large number of genes affected by the duplication of chromosome 12 likely contributes to the metabolome differences observed between the strains; for example the right arm contains two glycerol-1-phosphatases, amplification of which may be responsible for increased glycerol levels in F0. However, more than half the genes on this chromosome have no functional annotation, leaving many potential contributors unknown.

### F0 and F2 are equally virulent in nematode and murine models but F2 displays compromised dissemination

**Figure 6:**
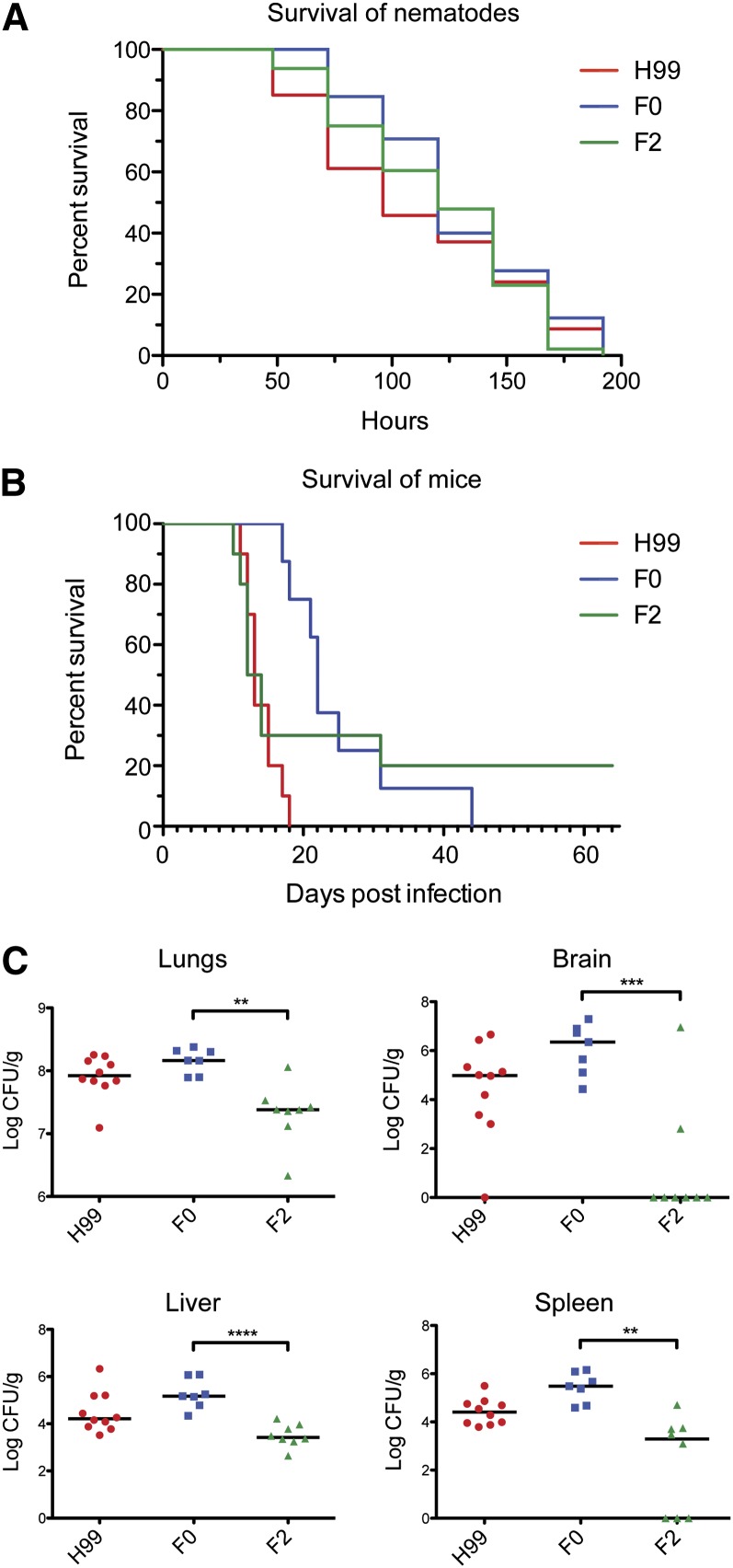
F0 and F2 exhibit significantly different dissemination patterns despite apparently similar virulence. (A) No significant difference in survival was seen between each of the strains when young adult *C. elegans* worms were cultured on indicated strains. (B) A murine virulence assay revealed no significant difference in virulence between F0 and F2; H99 was significantly more virulent than F0 (*P* = 0.0001). (C) F0 and F2 exhibited significantly different patterns of organ burden: *P < 0.05, **P < 0.01, ***P < 0.001, ****P < 0.0001; Student’s two-tailed *t*-test, two sample, equal variance (liver) or unequal variance (lungs, brain, spleen).

To determine whether the microevolutionary changes observed between strains F0 and F2 are associated with a selective advantage *in vivo*, we conducted virulence assays in two separate animal model systems. In the nematode infection model, no significant change in survival time of *Caenorhabditis elegans* was observed when cultured on strains F0 and F2 over 8 d (Figure 6A). In the murine inhalation model, we initially observed apparently similar virulence between strains F2 and H99, whereas F0 appeared attenuated (Figure 6B). However, two mice cleared the infection with strain F2, whereas all F0-infected mice died. Overall, survival times did not differ significantly between F0 and F2. Analysis of fungal burden within multiple organs, however, revealed significant differences in the progression of infection between the two strains: F2 cells disseminated from the lungs poorly, and failed to reach the brain in all but two mice (Figure 6C). Organ colony-forming unit per gram was significantly reduced for the brain, liver, and spleen of F2-infected mice, and fungal burden in the lungs themselves was also reduced. Given it was isolated from the CNS, this dissemination defect suggests the evolution of F2 after penetration of the blood brain barrier by the infecting ancestral strain.

### Mutation of the ARID protein occurs in multiple, independent serial isolates

Given no clear association between the genes on chromosome 12 and the observed phenotypes, we decided to investigate the ARID-containing gene affected by the single unique mutation identified in F2. We thus performed sequencing of this gene in our existing collection of 14 clinical isolate series. To our surprise, despite the relatively small sample size, we discovered a second series in which a mutation disrupts this same gene in a relapse isolate, this time in the third of three isolates denoted G0, G1, and G2. Probing of Southern blotted G2 DNA revealed that a translocation interrupts exon 3 of the ARID-containing gene (results not shown), which would result in a truncated protein of approximately 500 residues (of 1543). The disruption of this gene in a second, unrelated relapse isolate suggests a potential role in disease recurrence. Further investigation revealed G2 exhibited similar growth inhibition on carbon sources as observed in F2, as well as reduced capsule size. None of the strains in the series produced significant amounts of melanin at 37°. Reintroduction of the ARID-containing gene into both F2 and G2 mostly abolished the carbon source phenotype in both strains (Figure S6). Virulence factor testing revealed melanin production was not restored in F2 however capsule size increased in both complemented strains (Figure S7 and Figure S8).

**Figure 7:**
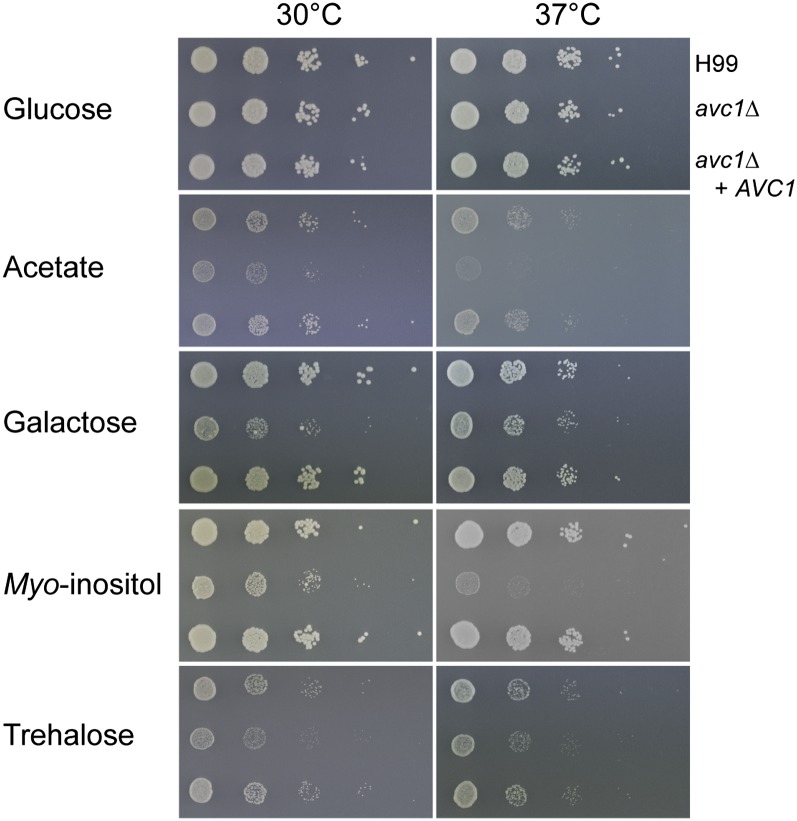
The growth of the deletion mutant *avc1*Δ resembles that of relapse isolate F2 on alternate carbon sources. Growth assays on minimal media supplemented with various carbon sources. Growth inhibition of *avc1*Δ is abolished after reintroduction of the gene.

**Figure 8:**
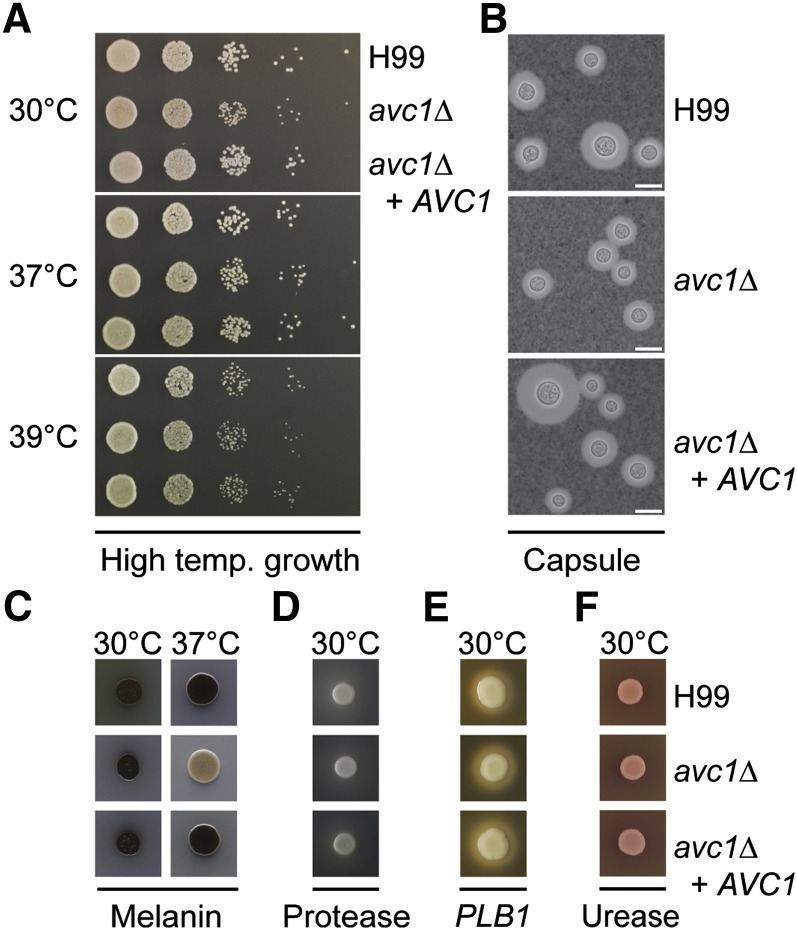
Capsule and melanin production are inhibited in *avc1*Δ. (A) Growth assays at 30°, human body temperature of 37° and febrile body temperature of 39° on YPD; no change was observed following deletion of *AVC1*. (B) India ink staining under light microscopy reveals the capsule; capsule production is inhibited in *avc1*Δ and restored following reintroduction of the gene. Scale bar is 10 μM. (C) Melanization on l-DOPA containing media is inhibited in *avc1*Δ and restored following reintroduction of the gene. (D−F) Comparable levels of protease, phospholipase, and urease production were observed in all strains when grown on bovine serum albumin, egg yolk, and Christensen’s agar, respectively.

**Figure 9:**
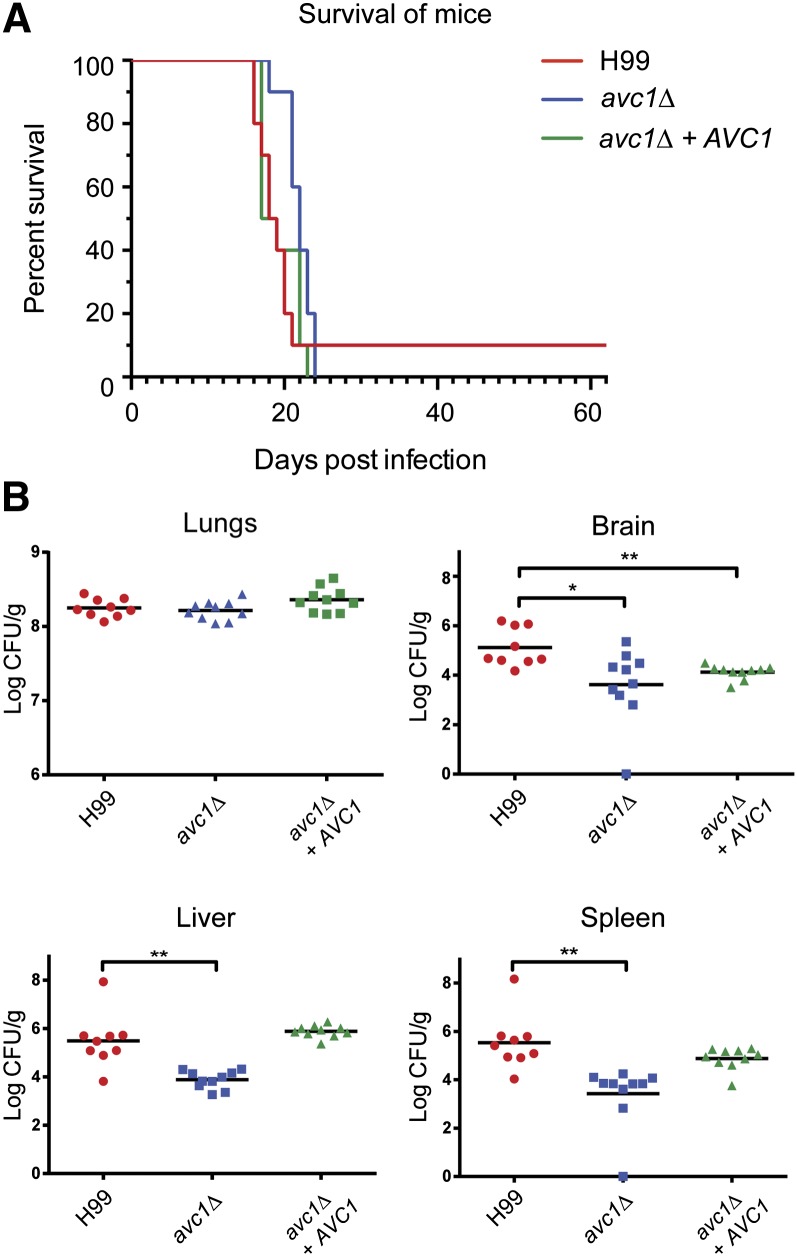
The deletion mutant *avc1*Δ exhibits reduced virulence and altered dissemination. (A) A murine virulence assay revealed a significant difference in virulence between H99 and *avc1*Δ (*P* = 0.0077); no significant difference was observed between H99 and *avc1*Δ + *AVC1*. (B) H99 and *avc1*Δ exhibited significantly different patterns of organ burden: *P < 0.05, **P < 0.01; Student’s two-tailed *t*-test, two sample, equal variance [lungs, brain (*avc1*Δ), spleen (*avc1*Δ)] or unequal variance [brain (*avc1*Δ + *AVC1*), liver, spleen (*avc1*Δ + *AVC1*)].

A deletion mutant was subsequently created in the H99 background and revealed similar phenotypic characteristics: reduced growth on a selection of carbon sources, capsule inhibition, and poor melanization at 37°. We were able to rescue all three of these phenotypes through reintroduction of the gene (Figures 7 and 8). We therefore dubbed the gene *AVC1* for ARID-containing regulator of virulence traits and carbon assimilation. The consistency of the carbon source phenotype between F2 and G2 and *avc1Δ* indicates the known glucose-related phenotype of H99 is not distorting these results (Morrow *et al.* 2012). We also tested the three strains in the presence of fluconazole and found slightly increased resistance when *AVC1* is disrupted, which was ablated when the gene was reintroduced into *avc1Δ* and F2, but not G2, suggesting the effect is genotype dependent (Figure S9). Significantly, infection with *avc1Δ* resulted in a reduction in fungal burden in a similar manner to that observed in F2, although not as severe, as more isolates successfully penetrated the CNS (Figure 9). However, unlike in F2, growth in the lungs was not inhibited in *avc1Δ*. We also observed an overall significant reduction in virulence of *avc1*Δ which was not seen in F2.

## Discussion

One of the challenges regarding the exploration of within-host microevolution is conclusively demonstrating the relationship between serial isolates. Traditionally, it was believed that most relapse was caused by the original infecting isolate and that these reinfections often corresponded with karyotypic change. More recently it has been shown that mixed infections occur in up to 20% of cases (Desnos-Ollivier *et al.* 2010) and karyotypic change described as microevolutionary may in fact reflect infection with other unrelated strains. Although MLST analysis has been used to distinguish sequential isolates from co-infection, the resolution of this technique limits the capacity for differentiation as evidenced by our MLST result comparing F0 and F2 with H99. Sequencing technology now permits complete sequencing of serial isolates, and our preliminary work in a larger collection of series has shown that strain relationships fall within two distinct groups: those that differ by thousands of SNVs, which we propose are coinfections, and those that differ by only a few, which we propose are cases of microevolution, such as occurs in F0 and F2. Significantly, our results indicate the evolution of multiple divergent lineages during infection. Although we cannot conclusively state the timeframe over which these mutations occurred, strain F0 had already accumulated unique changes from the common infecting ancestor at the time of initial diagnosis. This is in line with the observed prevalence of rapid and large-scale change in studies of *S. cerevisiae* (Dunham *et al.* 2002; Lynch *et al.* 2008) as well as other fungi such as *C. glabrata* (Polakova *et al.* 2009) and *Mycosphaerella graminicola* (Stukenbrock *et al.* 2010). The dissemination defect seen in F2 supports its evolution in-host, once the infection had reached the CNS. These findings give further credence to the idea that fungal pathogens may possess dedicated mechanisms for rapidly generating such large scale changes as a means of adaptation (Selmecki *et al.* 2010) especially given the stable genome inheritance of var. *grubii* since divergence from var. *neoformans* 20 million years ago.

### Aneuploidy of chromosome 12 occurs in both F0 and F2

The development of aneuploidy as an evolutionary process has been extensively described in multiple yeast species, both *in vitro* and *in vivo* (Kellis *et al.* 2004; Selmecki *et al.* 2006; Polakova *et al.* 2009). The associated gene duplications can have a profound effect on the proteome (Pavelka *et al.* 2010) and may also be a substrate for neofunctionalization (Ohno 1970) through accelerated mutation in gene copies that are largely free of selection (Kellis *et al.* 2004). Although we did not observe an increase in mutation rate on chromosome 12 using a crude measure of SNV per nucleotide, we do not know whether this duplication event occurred before or during infection; if it did occur during infection, the timeframe since its appearance may be too short to detect an increase in accumulation of mutations.

Tolerance of aneuploidy is contentious; aneuploidy is common and well tolerated in *C. albicans* (Selmecki *et al.* 2009), but demonstrated to be detrimental or advantageous in *S. cerevisiae*, presumably depending on genetic context and growth conditions (Torres *et al.* 2007; Pavelka *et al.* 2010). Although certain disomic *C. neoformans* strains have displayed impaired growth and attenuated virulence in mice (Sionov *et al.* 2010), many are afforded increased azole resistance. Simultaneous amplification of multiple chromosomes has been linked to changes in melanin production in *C. neoformans* (Hu *et al.* 2011). Accumulating examples of aneuploidies suggest chromosomal amplification as an important mechanism of variation in *C. neoformans*. Our data support short-term tolerance of aneuploidy in *C. neoformans* with minimal phenotypic consequences.

### Disruption of transcriptional regulator *AVC1* in F2

The ARID is associated with chromatin remodeling, and loss of such a gene may be pleiotropic and confer broad phenotypic deviation, as observed in other *C. neoformans* regulatory gene mutants (Cheon *et al.* 2011; Lee *et al.* 2011). ARID-containing proteins are found in diverse species, including humans, plants, insects, and fungi, and can bind DNA in both a sequence-specific and nonsequence-specific manner (Kortschak *et al.* 2000). Seven subfamilies exist in mammalian and insect genomes: ARID1 to ARID5 and JARID1 and JARID2. Although originally named for their interaction with AT-rich sequences, only two of the subfamilies, ARID3 and ARID5, show AT-rich site preference (Patsialou *et al.* 2005). Orthologs of ARID1, JARID1 and potentially ARID2 and JARID2 have been identified in fungi (Wilsker *et al.* 2005). Swi1, an ARID1 ortholog in *S. cerevisiae*, is a component of the SWI/SNF chromatin remodeling complex that regulates a wide variety of transcripts, including invertase (*SUC2*) and galactokinase (*GAL1*) (Sudarsanam and Winston 2000). *S. cerevisiae* has one other annotated ARID-containing protein (Ecm5), whereas *Schizosaccharomyces pombe* contains four including Sol1, a SWI/SNF component. *AVC1* is one of three annotated ARID-containing proteins in *C. neoformans* var. *grubii*; CNAG_00240 on chromosome 1 has no known function while CNAG_07411, within the mating-type locus on chromosome 5, is the transcription factor Rum1.

Typically, although amino acid identity is high within the ARID region within a subfamily (70−80%), between subfamilies identity drops to below 30%. Fungal ARID regions drop significantly in identity, however, with less than 30% identity between Saccharomyces Swi1 and other members of the ARID1 subfamily, making ortholog identification difficult. Members of the ARID1 subfamily contain glutamine-rich regions and LXXLL motifs, although the position of these is not conserved between family members. Avc1 contains both glutamine-rich regions and a single LXXLL motif, as well as proline and alanine-rich regions. The other uncharacterized ARID-containing protein in var. *grubii*, CNAG_00240, also contains glutamine-rich regions but no LXXLL motifs. BLAST of both ARID regions yields Swi1 orthologs for Avc1 only. Further work is needed to determine whether either of these genes is the *Cryptococcus* homolog of *SWI1*.

### Multiple independent disruptions of *AVC1* indicate a role in relapse

The significance of *AVC1* is increased by its being identified in two relapse isolates, indicative of a role in promoting infection persistence. However, phenotypic analysis revealed decreased production of two key virulence traits, capsule and melanin, and an overall decrease in virulence was observed in *avc1*Δ. Clinical isolates of *C. neoformans* produce inconsistent virulence profiles, however, and melanin in particular is frequently variable in these isolates (Clancy *et al.* 2006; Pereira *et al.* 2009). Mutants lacking melanin production due to mutation in the laccase gene *LAC1* have been shown to have compromised ability to escape from the lungs, whereas growth in the brain is unaffected (Noverr *et al.* 2004). Although both F2 and *avc1*Δ exhibited a degree of dissemination defect, the liver was colonized in all mice, indicating the infection was not trapped in the lungs. However, often gene products affecting melanin also affect virulence and/or dissemination independently (Chun *et al.* 2007; Waterman *et al.* 2007). Changes in carbon source utilization as well as melanin production at 37° have also been observed in *snf1* mutants (Hu *et al.* 2008). Snf1, a serine/threonine protein kinase, was found to increase the expression of *LAC1* under low glucose conditions and mutants were unable to persist in the brain, rendering them avirulent. Although the majority of F2 infections failed to reach the brain, the titer when infection was established was high, indicating F2 was unable to reach the brain rather than unable to persist. Capsule inhibition has been shown to produce strains both with limited growth in the lungs and dissemination to the brain (Wilder *et al.* 2002). Although F2 showed lower colony-forming unit in the lungs, *avc1*Δ did not, indicating this is unlikely to be contributing to the virulence phenotype of *avc1*Δ. Future work consisting of virulence studies utilizing the intracranial infection model will help to clarify the role of *AVC1* in infection without the confounding variable of CNS penetration.

This work reveals the simultaneous evolution of multiple lineages derived from a very recent common ancestor during *C. neoformans* infection. The observed aneuploidies support via parsimony the presence of a chromosome 12 duplication in the common ancestor; however, we cannot determine whether this arose during infection or was present *ex vivo*. The CNS dissemination defect in F2 (coupled with its recovery 77 d after the initial diagnosis) suggests it arose after penetration of the CNS, and the associated increase in fluconazole resistance may have given the isolate a selective advantage after antifungal therapy. Disruption of a single gene, *AVC1*, in separate relapse isolates supports a role for this gene in disease persistence or relapse. These results illustrate the importance of population diversification in pathogenic organisms and the clonal expansion of the fittest member as selective pressures alter. Studies of more serial isolates may facilitate the identification of further genomic variation pertinent to clinical relapse and persistence associated with this important fungal pathogen.

## Supplementary Material

Supporting Information
