# Supplementary material for: Comparative Genomics of Serial Isolates of Cryptococcus neoformans Reveals Gene Associated With Carbon Utilization and Virulence
Source: G3 (Bethesda). 2013 Apr 1;3(4):675–86. doi: 10.1534/g3.113.005660 (PMC3618354; doi:10.1534/g3.113.005660)
Supplement: Supporting Information [file supp_3_4_675__index.html]

Comparative Genomics of Serial Isolates of Cryptococcus neoformans Reveals Gene Associated With Carbon Utilization and Virulence — Supporting Information 

# Comparative Genomics of Serial Isolates of *Cryptococcus neoformans* Reveals Gene Associated With Carbon Utilization and Virulence

## Supporting Information for Ormerod *et al.*, 2013

**Files in this Data Supplement:**

- Supporting Information - Figures S1-S9, File S1, and Tables S1-S4 (PDF, 3 MB)
- Figure S1 - Transposon profiles of F0 and F2 are the same for Cnirt2, Tcn1, Tcn2 and Tcn4 but different for Tcn6, a highly mobile transposon (PDF, 490 KB)
- Figure S2 - F0 and F2 exhibit similar responses to a variety of stresses (PDF, 712 KB)
- Figure S3 - Melanin production in F2 is reduced on multiple nitrogen sources (PDF, 225 KB)
- Figure S4 - F0 and F2 exhibit different growth on alternate carbon sources(PDF, 139 KB)
- Figure S5 - Pairwise comparisons of metabolic profiles with principal components analysis (PDF, 237 KB)
- Figure S6 - Reintroduction of ARID-containing gene *AVC1* rescues growth on alternate carbon sources (PDF, 122 KB)
- Figure S7 - Reintroduction of ARID-containing *AVC1* rescues capsule but not melanin production in F2 (PDF, 469 KB)
- Figure S8 - Reintroduction of ARID-containing *AVC1* rescues capsule production in G2 (PDF, 581 KB)
- Figure S9 - Deletion of *AVC1* increases resistance to fluconazole (PDF, 229 KB)
- File S1 - Supplementary materials and methods (PDF, 135 KB)
- Table S1 - S1A) Indels identified between H99 and F0 and F2 within genes with functional annotation S1B) Structural variation identified between H99 and F0 and F2 (PDF, 113 KB)
- Table S2 - Genes on the left arm of chromosome 12 (PDF, 105 KB)
- Table S3 - Statistical parameters of multivariate PCA models of metabolomic data (PDF, 76 KB)
- Table S4 - Primers used in this study (PDF, 82 KB)
