# Supplementary material for: Comparative Genomics of Serial Isolates of Cryptococcus neoformans Reveals Gene Associated With Carbon Utilization and Virulence
Source: G3 (Bethesda). 2013 Apr 1;3(4):675–86. doi: 10.1534/g3.113.005660 (PMC3618354; doi:10.1534/g3.113.005660)
Supplement: Supporting Information [file supp_g3.113.005660_005660SI.pdf]

G3·Genes | Genomes | Genetics

Comparative genomics of serial isolates of *Cryptococcus neoformans* reveals gene associated with carbon utilization and virulence

Kate L. Ormerod[*,§], Carl A. Morrow[*,§], Eve W. L. Chow[*,§], I. Russel Lee[*,§], Samantha D. M. Arras[*,§], Horst Joachim Schirra[†,§], Gary M. Cox[‡], Bettina C. Fries[§§,**] and James A. Fraser[*,§1]

Australian Infectious Diseases Research Centre,
University of Queensland, Brisbane, QLD 4072 Australia[*]

School of Chemistry and Molecular Biosciences,
University of Queensland, Brisbane, QLD 4072 Australia[§]

Centre for Advanced Imaging,
University of Queensland, Brisbane, QLD 4072 Australia[†]

Department of Medicine,
Mycology Research Unit,
Duke University, Durham, NC 27710 USA[‡]

Department of Microbiology & Immunology,
Albert Einstein College of Medicine, Bronx, NY 10461 USA[§§]

Department of Medicine,
Albert Einstein College of Medicine, Bronx, NY 10461 USA[**]

[1]Corresponding author:
358 Molecular Biosciences Building, Cooper Road
The University of Queensland
Brisbane, QLD 4072 Australia
Phone: +61 7 3365 4868
Fax: +61 7 3365 4273
Email: jafraser@uq.edu.au

DOI: 10.1534/g3.113.005660

(A)

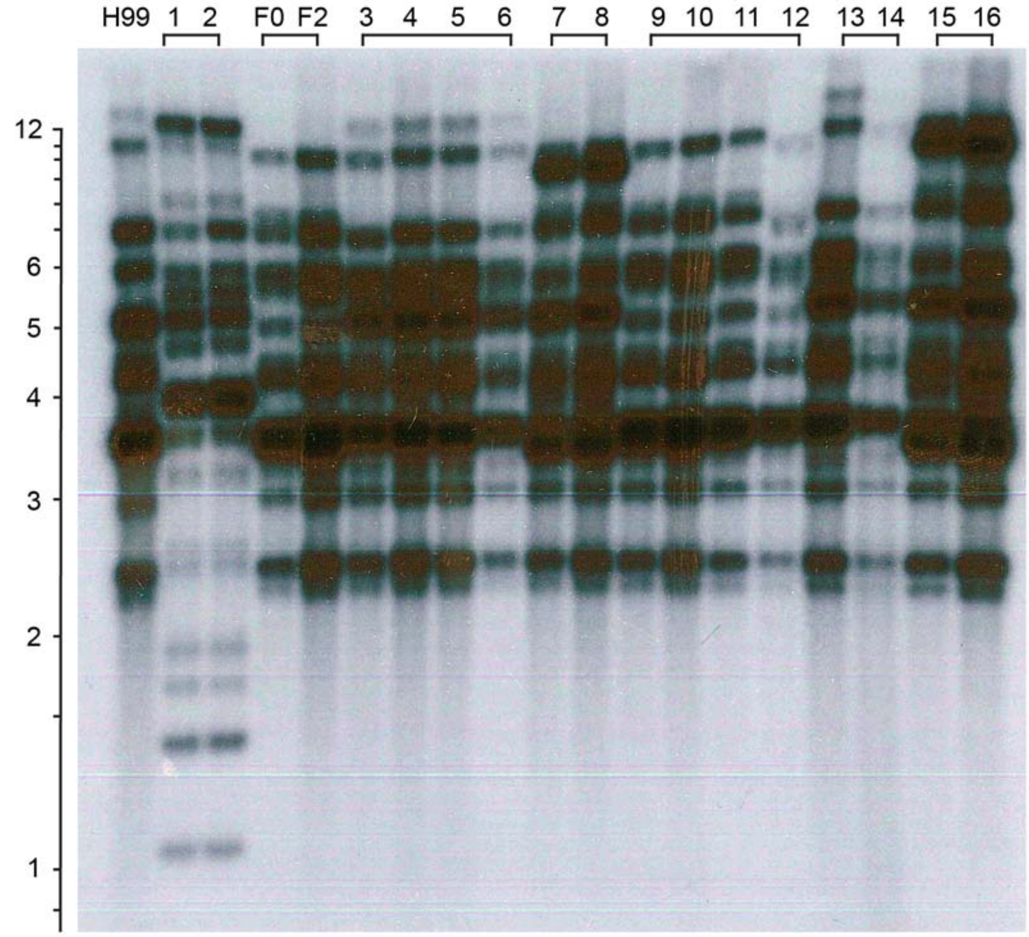

(B)

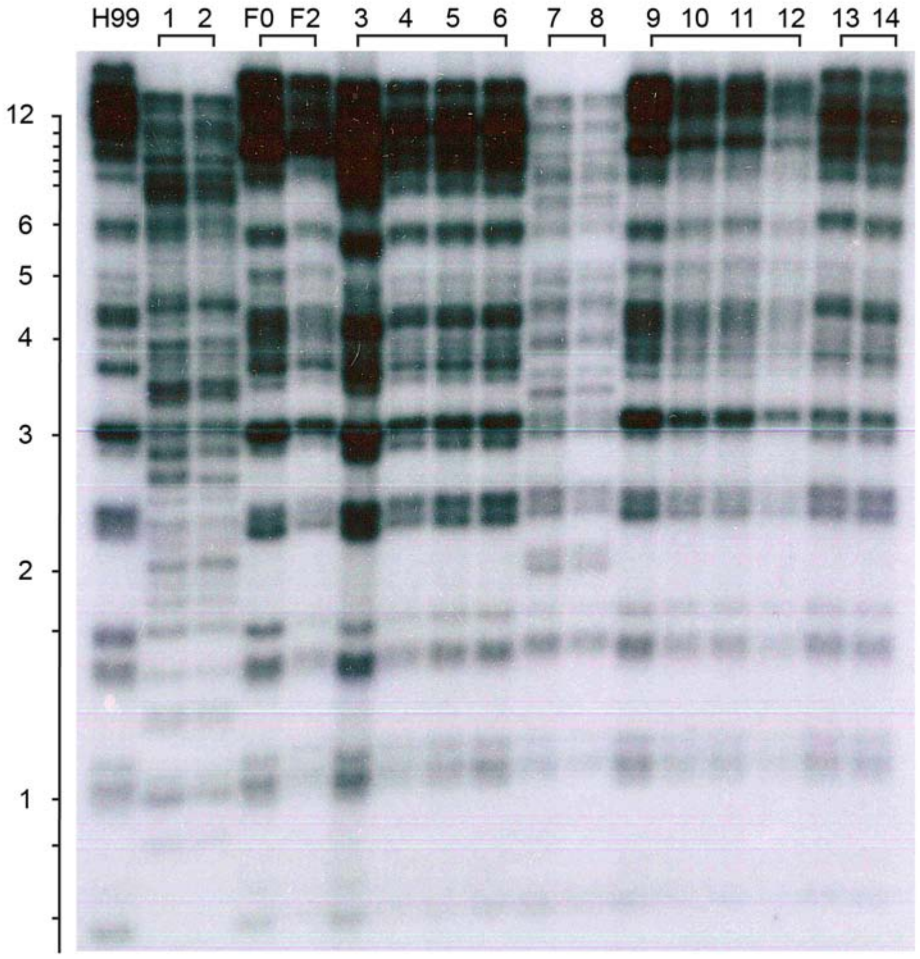

(C)

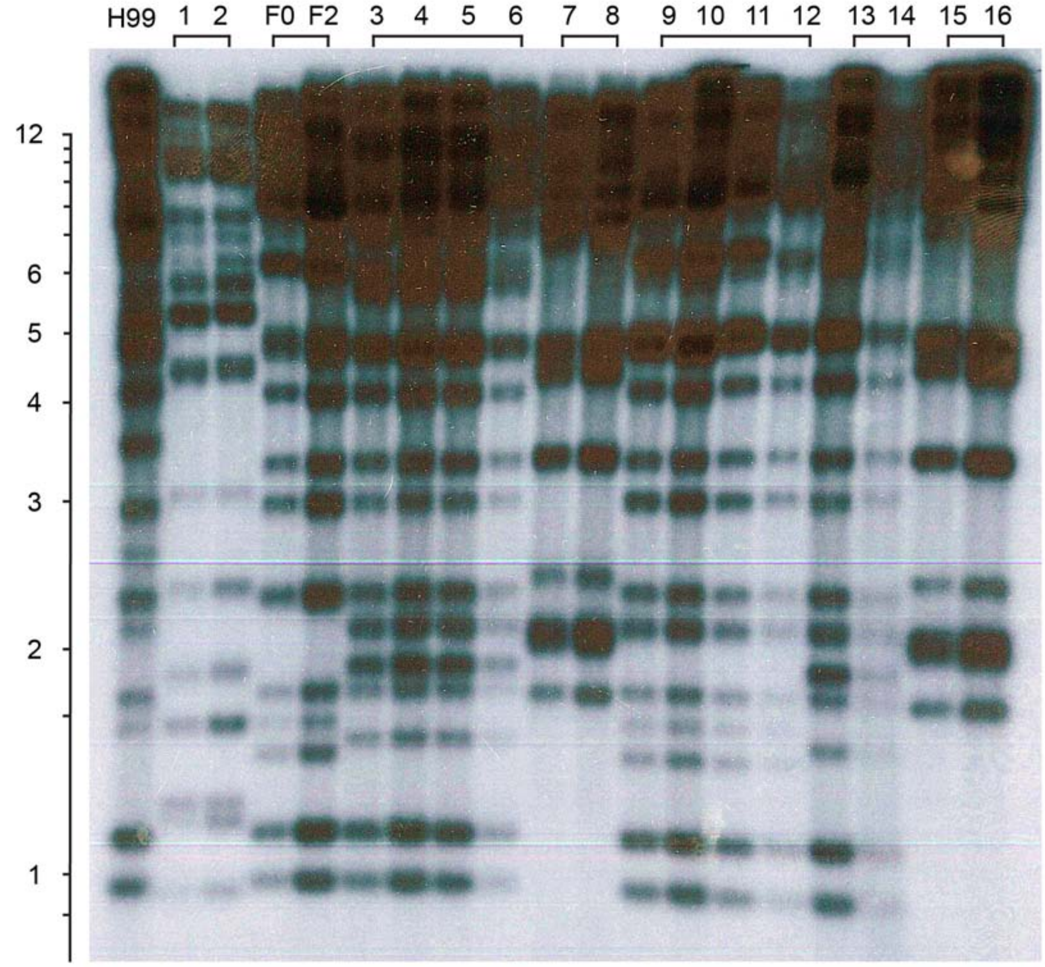

(D)

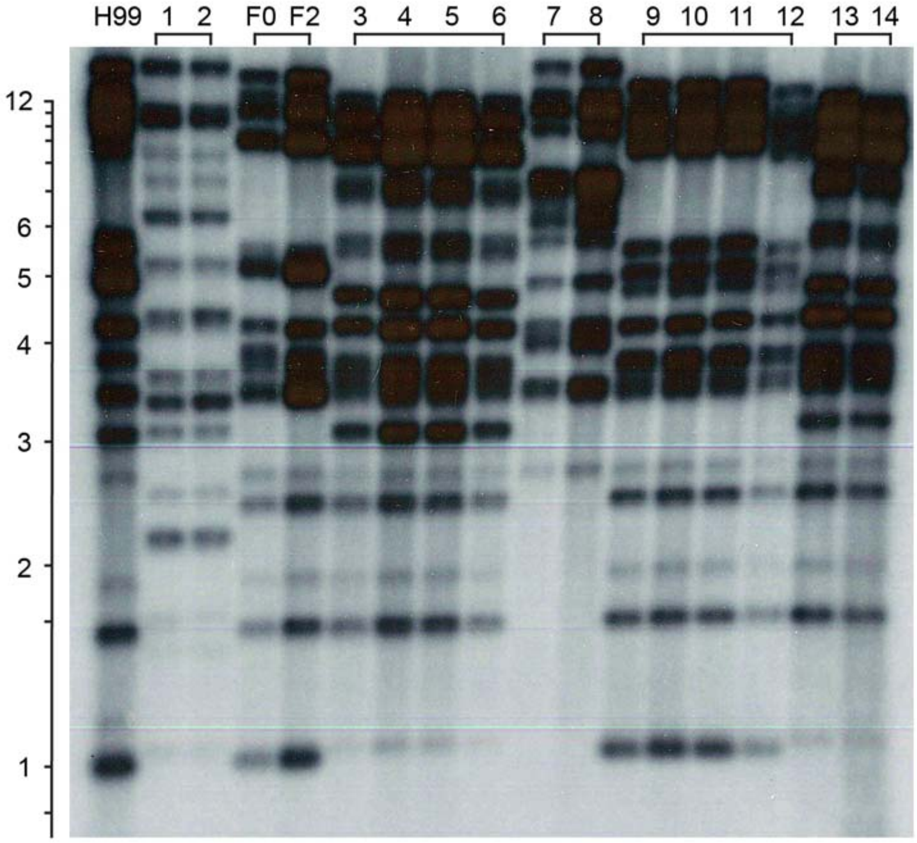

(E)

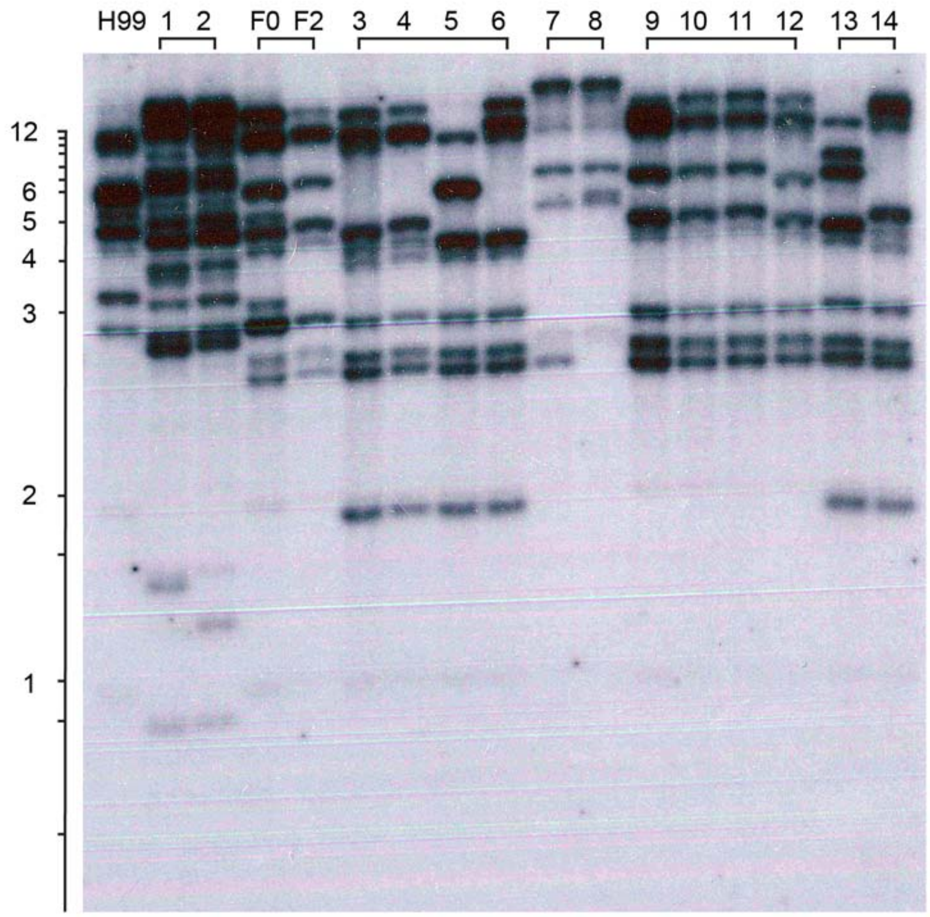

(F)

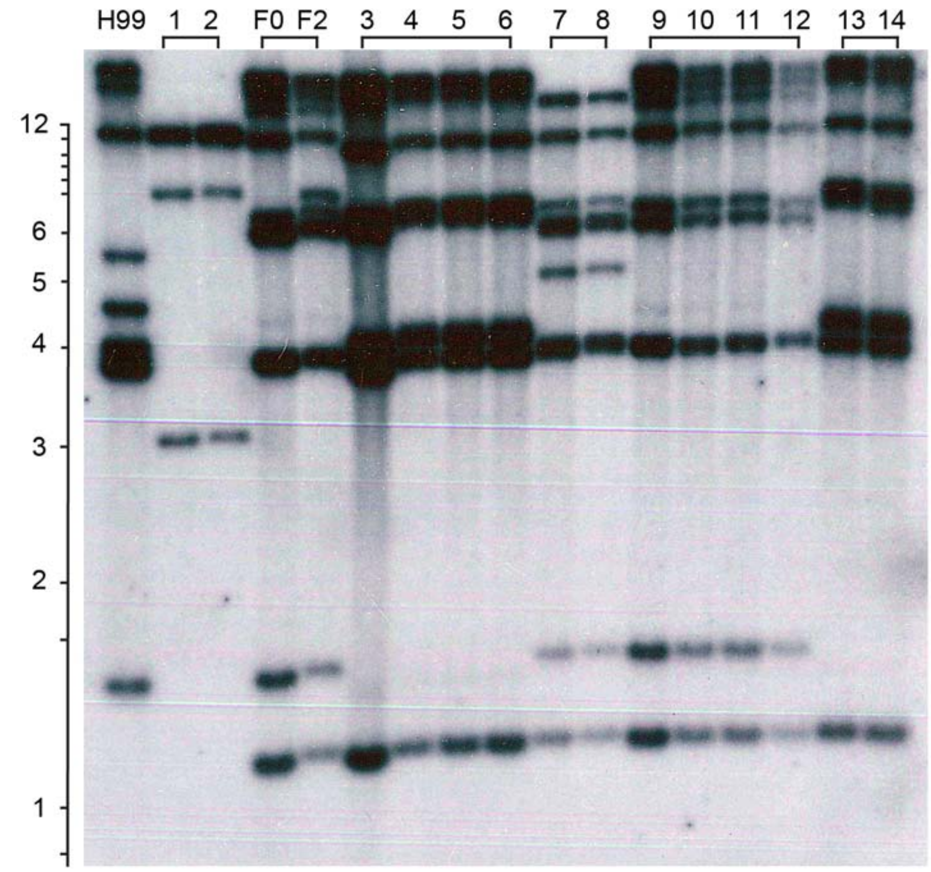

FIGURE S1 Transposon profiles of F0 and F2 are the same for Cnirt2, Tcn1, Tcn2 and Tcn4 but different for Tcn6, a highly mobile transposon. Southern blot analysis of serial isolates (sets marked with rectangles) probed with transposon fragments of **(A)** Cnirt2, **(B)** Tcn1, **(C)** Tcn2, **(D)** Tcn4, **(E)** Tcn6 internal region, **(F)** Tcn6 LTR. Strains 1 to 16 are serial clinical isolates that have not as yet been characterized.

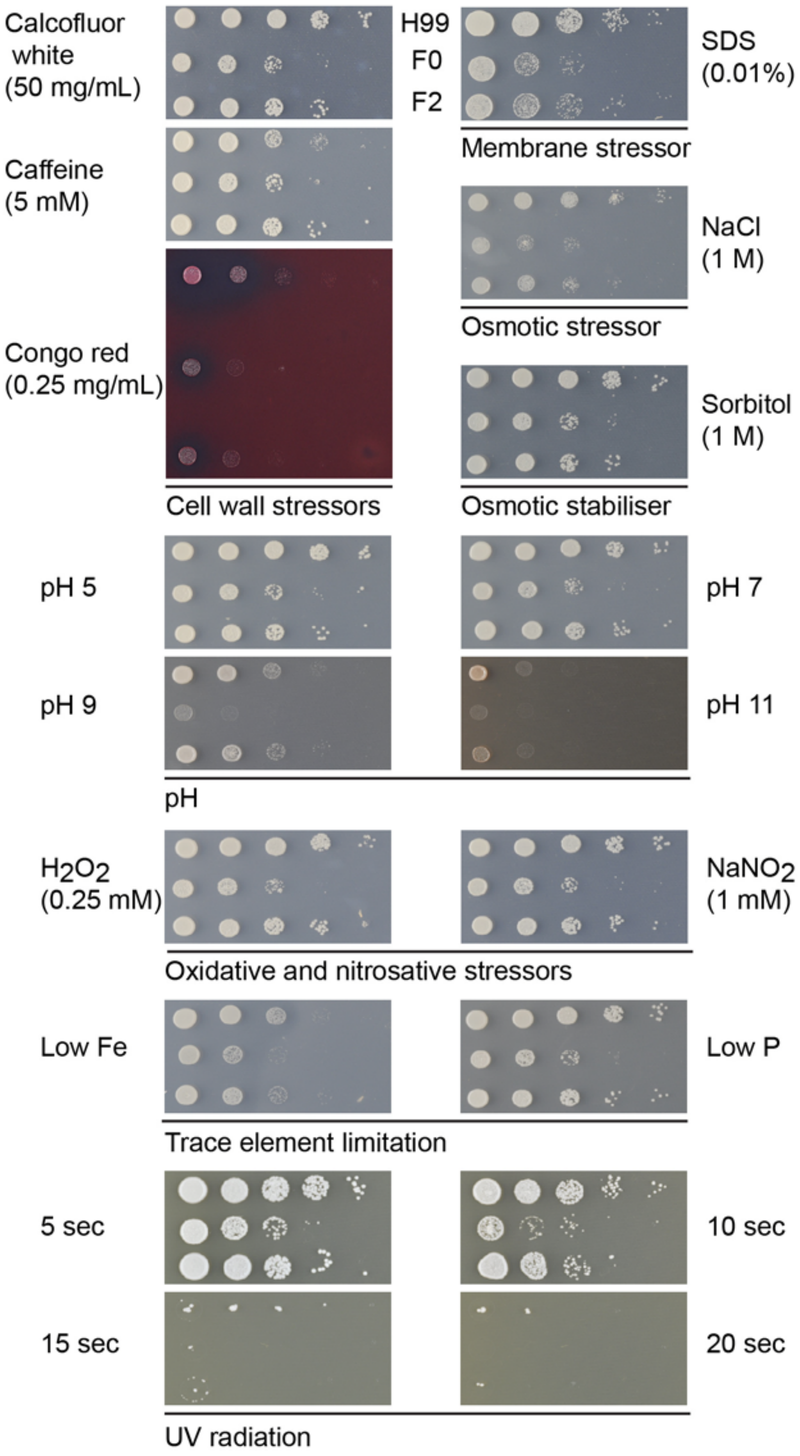

FIGURE S2 F0 and F2 exhibit similar responses to a variety of stresses. 10-fold serial dilutions of indicated strains were spotted onto minimal media supplemented with various stressors and incubated at 30˚ for 2-3 days. The growth defect of F0 is not exacerbated by growth on cell wall stressors. F0 and F2 show increased sensitivity to cell membrane and osmotic stress. No difference in growth is seen on the osmotic stabilizer sorbitol. Increased sensitivity to high pH (9 and 11) is seen in F0. No increased sensitivity is seen in either F0 or F2 under selected oxidative or nitrosative stressors or nutrient limitation. No increased sensitivity is seen in F0 or F2 when exposed to UV radiation.

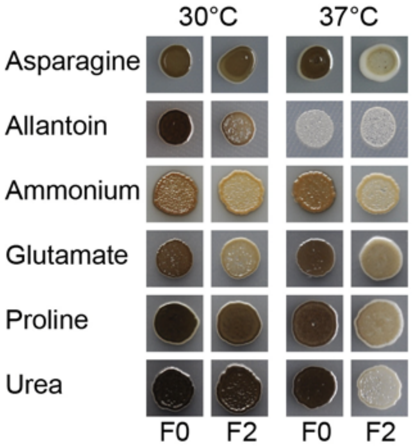

FIGURE S3 Melanin production in F2 is reduced on multiple nitrogen sources. When grown on L-DOPA containing Cryptococcus melanization media supplemented with various nitrogen sources, F2 exhibits a significant melanization defect at 37°, also visible on many nitrogen sources at 30°.

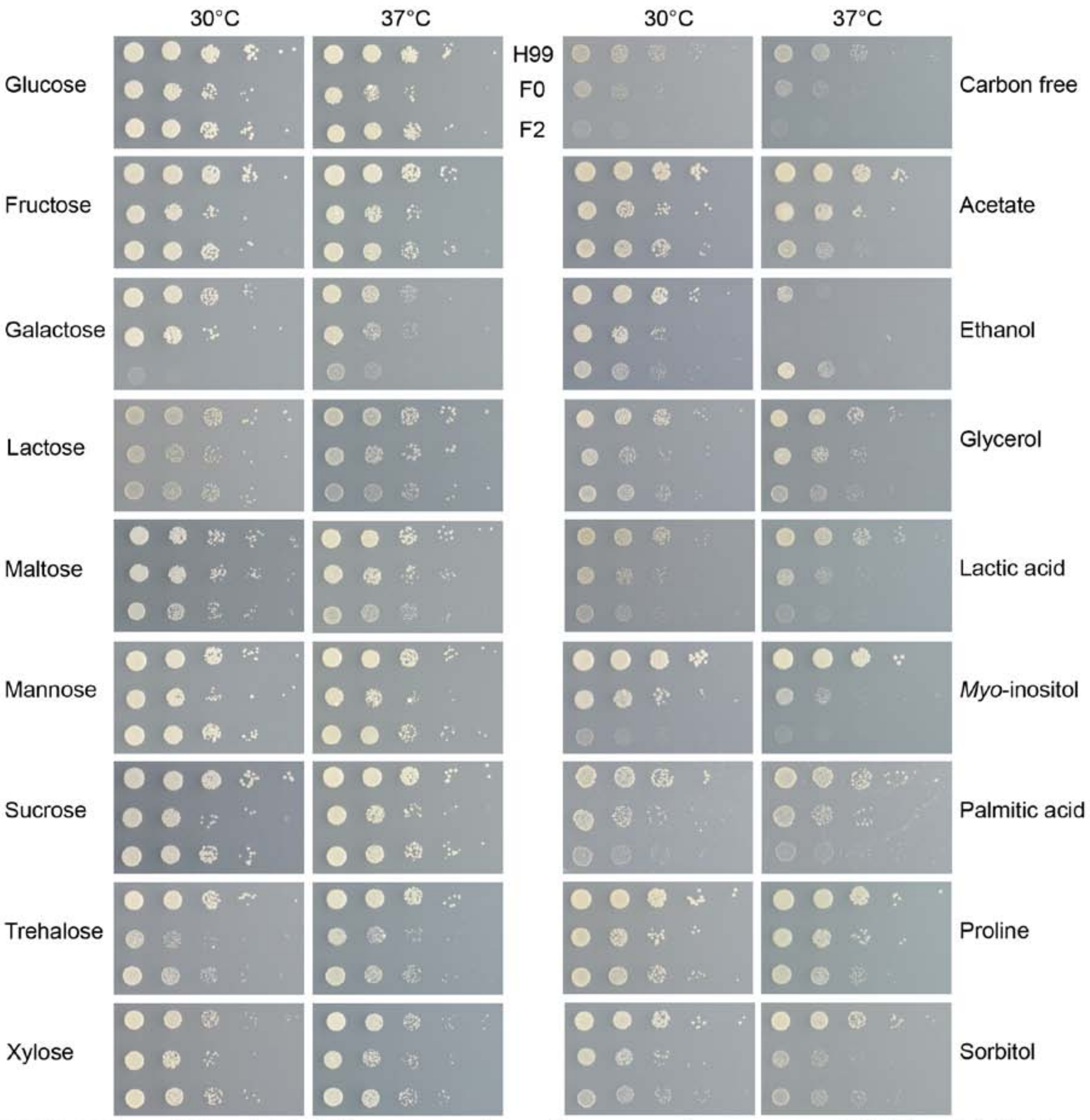

FIGURE S4 F0 and F2 exhibit different growth on alternate carbon sources. 10-fold serial dilutions of indicated strains were spotted onto minimal media supplemented with various carbon sources and incubated at 30 and 37° for 2 to 3 days.

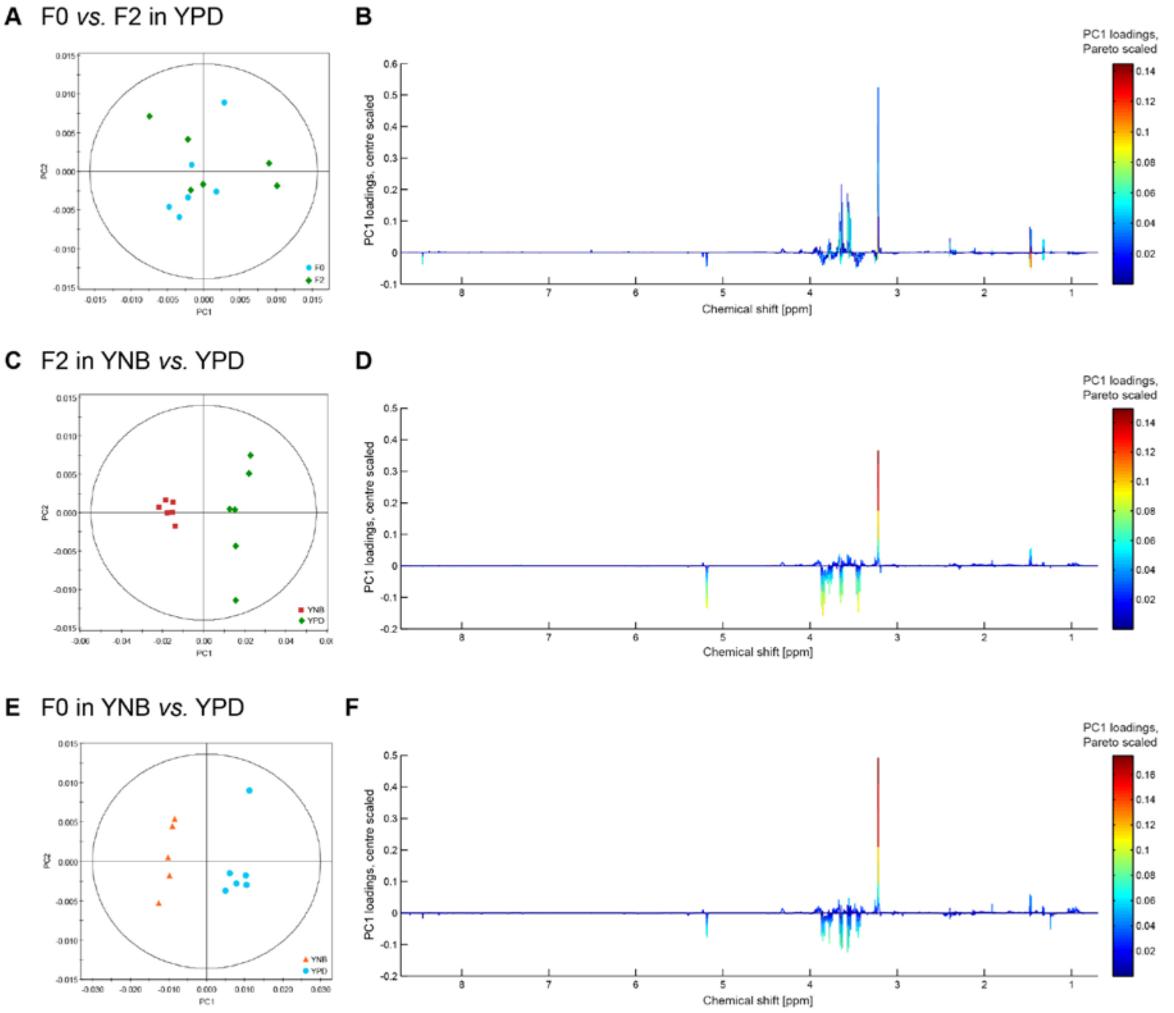

FIGURE S5 Pairwise comparisons of metabolic profiles with principal components analysis. (A) & **(B)** Comparison of F0 (cyan circles) with F2 (green diamonds) in YPD medium. **(C)** & **(D)** Comparison between F2 in YNB (red squares) and YPD (green diamonds) medium. **(E)** & **(F)** Comparison between F0 in YNB (orange triangles) and YPD (cyan circles) medium. **(A)**, **(C)** & **(E)**: Principal components analysis scores plots. Hotelling's 95% confidence range of the PCA model is indicated in the scores plots by an ellipse. **(B)**, **(D)** & **(F)**: Bivariate loadings plot with center scaled loadings coefficients shown as intensity, and Pareto scaled coefficients as heatmap.

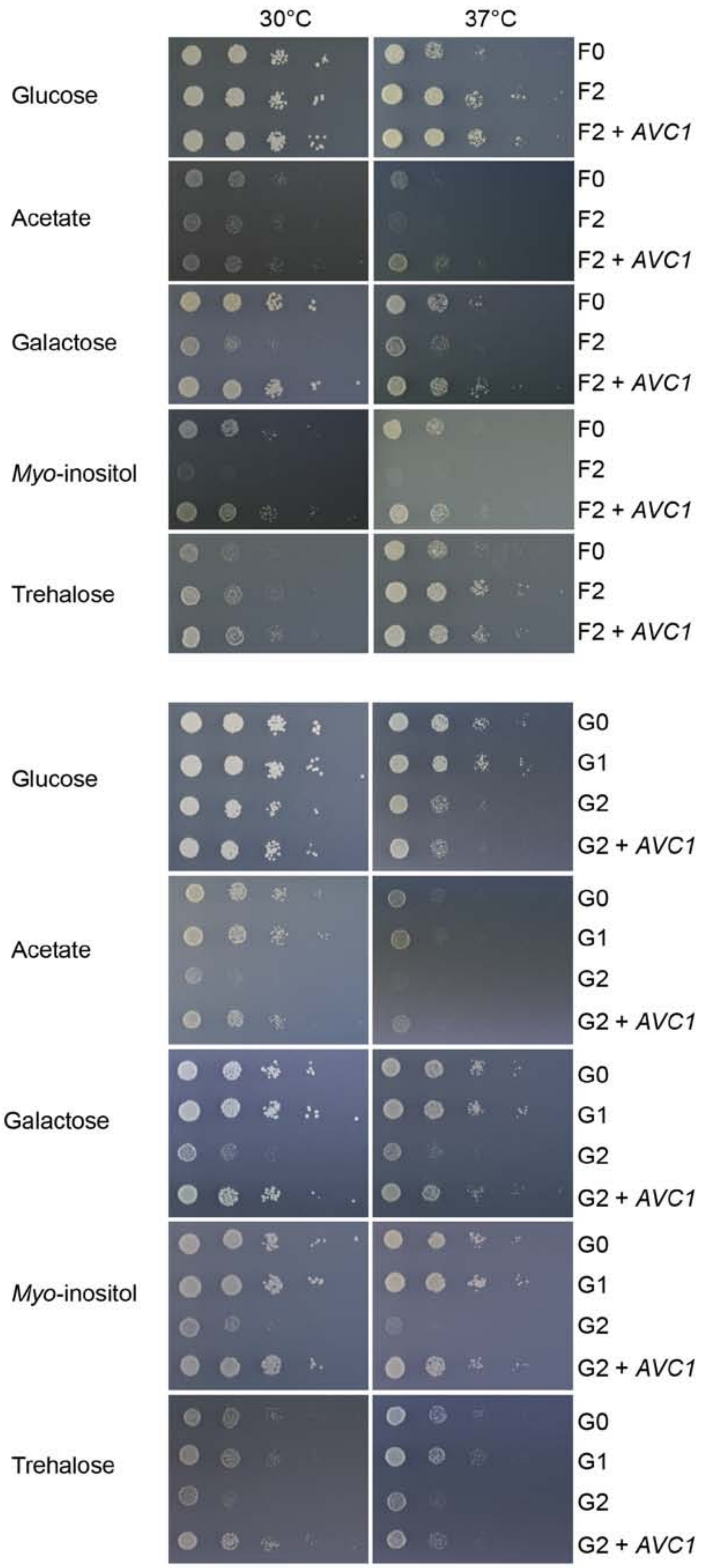

FIGURE S6 Reintroduction of ARID-containing gene *AVC1* rescues growth on alternate carbon sources. Growth assays on minimal media supplemented with various carbon sources.

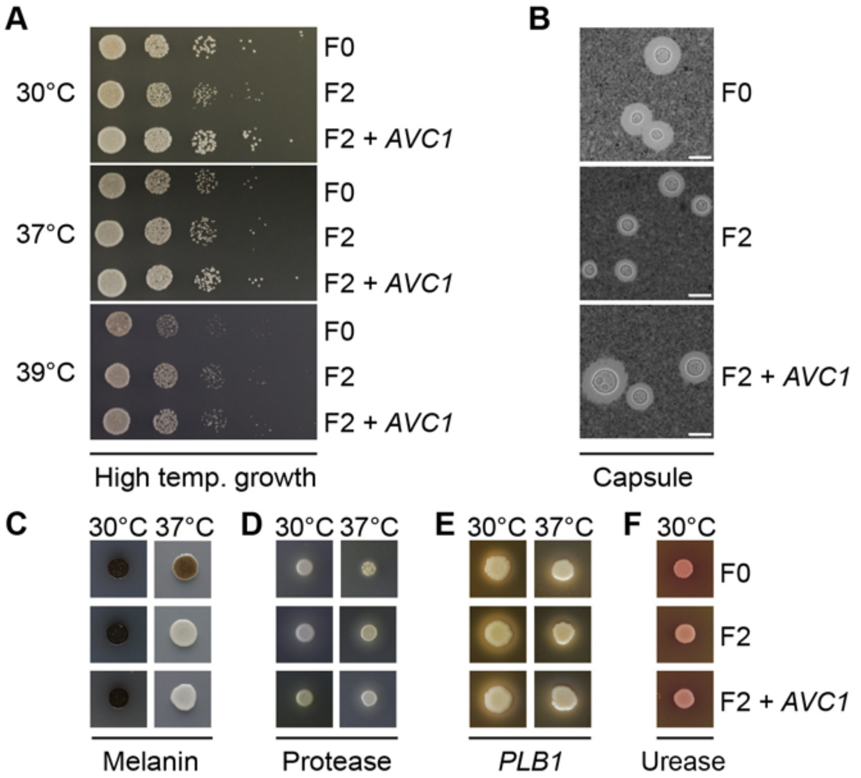

FIGURE S7 Reintroduction of ARID-containing *AVC1* rescues capsule but not melanin production in F2. (A) Growth assays at 30˚, human body temperature of 37˚ and febrile body temperature of 39˚ on YPD; no change was observed following reintroduction of *AVC1*. **(B)** India ink staining under light microscopy reveals the capsule; capsule production in F2 is increased following reintroduction of *AVC1*. Scale bar is 10 μM. **(C)** Melanization on L-DOPA containing media continued to be inhibited in F2 following reintroduction of *AVC1*. **(D)**, **(E)** & **(F)** Comparable levels of protease, phospholipase and urease production were observed following reintroduction of *AVC1* when strains were grown on BSA, egg yolk and Christensen's agar, respectively.

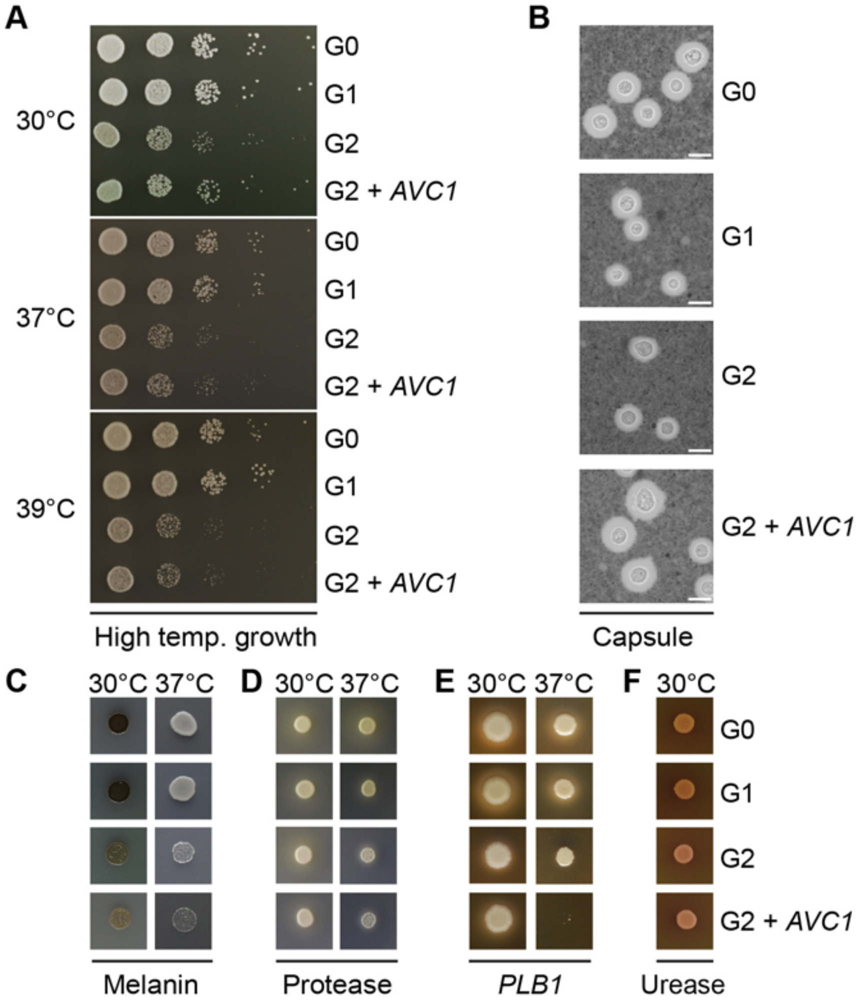

FIGURE S8 Reintroduction of ARID-containing *AVC1* rescues capsule production in G2. (A) Growth assays at 30˚, human body temperature of 37˚ and febrile body temperature of 39˚ on YPD; no change was observed following reintroduction of *AVC1*. **(B)** India ink staining under light microscopy reveals the capsule; capsule production in G2 is increased following reintroduction of *AVC1*. Scale bar is 10 µM. **(C)** Melanization on L-DOPA containing media continued to be inhibited in G2 following reintroduction of *AVC1*. **(D)**, **(E)** & **(F)** Comparable levels of protease, phospholipase and urease production were observed following reintroduction of *AVC1* when strains were grown on BSA, egg yolk and Christensen's agar, respectively. No growth was observed for G2 + *AVC1* on egg yolk agar at 37˚.

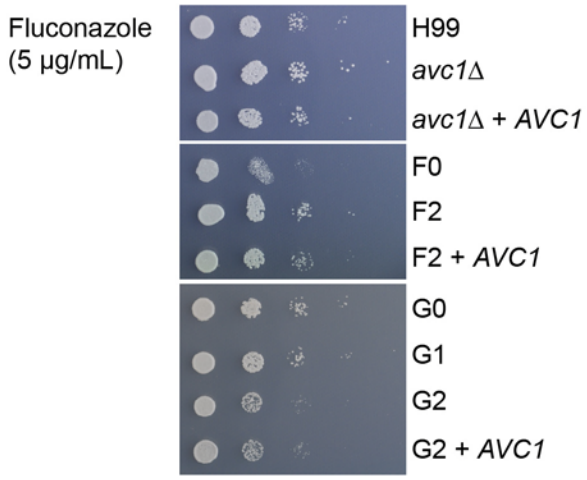

FIGURE S9 Deletion of *AVC1* increases resistance to fluconazole. Both *avc1*Δ and F2 exhibit increased growth on minimal media containing fluconazole (5 µg/mL) which is abolished when the gene is reintroduced. Growth is comparable between G2 and G2 + *AVC1*.

File S1

Supplementary materials and methods

Media

Cryptococcus strains were cultured in YPD (1% yeast extract, 2% Bacto-peptone, 2% glucose) and maintained at 4˚C on YPD solidified with 2% agar or stored at -80˚C in 15% glycerol. Phenotypic assays were conducted on YNB (yeast nitrogen base without amino acids and ammonium sulfate (BD, Franklin Lakes, NJ), 2% sugar or 10 mM carbon source, 10 mM nitrogen source, 2% agar). L-3,4-dihydroxyphenylalanine (L-DOPA) media with 10 mM nitrogen source for melanization assays was prepared as described (Chaskes and Tyndall 1975). Urease assays were conducted on Christensen's urea agar (Christensen 1946). Protease production was assayed using YNB with amino acids and ammonium sulfate supplemented with 2% glucose and 0.1% bovine serum albumin (Sigma-Aldrich, St Louis, MO). Phospholipase production was assayed on egg yolk agar as described (Chen *et al.* 1997). Capsule production was induced in liquid RPMI 1640 medium (Life Technologies, Carlsbad, CA) supplemented with 2% glucose and 10% foetal bovine serum (Life Technologies) as described (Zaragoza *et al.* 2003). *C. elegans* was maintained at 16ºC on nematode growth medium as described (Brenner 1974).

Phenotypic assays

Starter cultures were prepared by growth in YPD at 30ºC overnight with shaking, diluted to OD595 nm = 0.05 in water, then further diluted 10-fold in series. For growth, carbon utilization, melanization and stress assays, dilutions were spotted onto YPD, YNB supplemented with 2% sugar or 10 mM carbon source, L-DOPA or YNB supplemented with 5 mM caffeine, 0.25 mg/mL Congo red, 50 mg/mL calcofluor white, 0.01% SDS, 1 M sorbitol, 1 M NaCl, 0.25 mM H_2O_2 or 1 mM $NaNO_2$ as indicated. Images were taken following 2-3 days incubation at 30 (stress, phospholipase, protease, urease, nitrogen utilization), 30 and 37˚C (carbon utilization and melanization), or 30, 37 and 39˚C (growth). UV stress assay was conducted by exposing freshly spotted (YPD) cultures to 48 mJ/cm^2 UV light for 6, 12, 18 or 24 seconds in a UV Stratalinker (Stratagene). Capsule production was assayed at 30̊C with shaking and aliquots taken at 24 h. India ink (BD) staining was performed prior to visualization on a Zeiss Axioplan 2 epifluorescent/light microscope fitted with Axiocam greyscale camera running AxioVision AC software. Difference in capsule size was determined by calculating the ratio of capsule to cell diameter for each strain, measuring 50 cells per strain across 10 independent fields (Zaragoza *et al.* 2003). Statistical significance was determined using the unpaired, two-tailed *t*-test with *p* values <0.05 considered significant.

Fluconazole minimum inhibitory concentration determination

Minimum inhibitory concentration (MIC) assays were undertaken in biological triplicate following the broth microdilution protocol published by the Clinical and Laboratory Standards Institute (M27-A3) modified for *Cryptococcus* (Ghannoum *et al.* 1992). Fluconazole concentrations tested ranged from 0.1 to 50 µg/mL in two-fold dilutions. Plates were incubated at

35˚C for 72 h, scored visually at 24 h intervals and end-point reading taken electronically using a SpectraMax 250 (Molecular Devices). MIC_{90} (the lowest concentration of drug inhibiting ≥ 90% of growth) was recorded. *Candida albicans* (ATC90028)*, Candida krusei* (ATCC6258)*, Candida parapsilosis* (ATCC22019) and *C. neoformans* var. *grubii* (ATCC90113) were included as reference strains.

Read trimming and mapping

Quality was analyzed using FastQC (http://www.bioinformatics.bbsrc.ac.uk/projects/fastqc/). Reads were trimmed using CLC Genomics Workbench (4.6.1, CLC bio, Denmark) based on a phred-scaled quality score of 20 prior to mapping with this software. Post-trimming reads ≥ 30 nt were retained (77%, 78% and 77% respectively). BWA 0.5.9 (Li and Durbin 2009) was run on untrimmed reads. Reads were mapped using BWA and CLC Genomics Workbench using the unpublished, annotated H99 genome (www.broadinstitute.org/annotation/genome/cryptococcus_neoformans/MultiHome.html) as a reference. BWA was run with default settings. Marking of duplicate reads, realignment of reads around indels and recalibration of quality scores was then undertaken following the Genome Analysis Toolkit (GATK) pipeline (McKenna *et al.* 2010; DePristo *et al.* 2011). Recalibration was performed using a list of putative SNVs identified with GATK UnifiedGenotyper. Filters were applied as described (DePristo *et al.* 2011) with a required SNV frequency of 70% of reads. CLC Genomics Workbench was run using required read similarity of 95% and an insert size range of 130 to 300 bp. Other settings were left at default values.

***De novo* assembly**

SOAP *de novo* 1.05 (Li *et al.* 2008) and CLC Genomics Workbench were used to produce *de novo* assemblies using reads trimmed either with CLC Genomics Workbench as described above or with Trimmomatic (www.usadellab.org/cms/index.php?page=trimmomatic) using a quality cutoff of 20 across a sliding window of 4 nt, removing leading and trailing 3 nt from all reads and retaining post-trimming reads with length ≥ 30 nt. Assemblies were used as support for structural variation predictions. Separate *de novo* assemblies were run using unmapped reads in order to identity unique insertion sequences. Unmapped reads were extracted from BWA alignments using SAMtools 0.1.17 (Li *et al.* 2009) and Picard SamToFastq (picard.sourceforge.net). Assembled contigs were matched to the H99 genome using BLAST (Altschul *et al.* 1990). Potential insertion contigs from SOAP *de novo* were aligned to CLC Genomics Workbench contigs using BLAST to confirm the insert sequence.

Identification of repetitive regions

Transposable elements were identified using RepeatMasker (version open-3.3.0) (Smit *et al.* 1996-2010) with repeat library 20110419 utilizing the cross_match (version 0.990329) search engine (http://www.phrap.org/index.html). Additional

matches to known transposons of *C. neoformans* were identified via a BLAST search and annotated with blast2gffv3 (code.google.com/p/jperl/source/browse/trunk/scripts/Blast2Gff.pl?r=61). LTRHarvest (Ellinghaus *et al.* 2008) was used to predict LTR retrotransposons. Tandem repeats were identified using Tandem Repeats Finder (Benson 1999) and annotated using TRAP (Sobreira *et al.* 2006).

Genomic variation detection

Structural variation was detected using BreakDancer (Chen *et al.* 2009) (minimum mapping quality = 20, minimum supporting reads = 4), CREST (Wang *et al.* 2011) and Dindel (Albers *et al.* 2011). BreakDancer predictions with >90% confidence were examined manually. Dindel predictions present in both F0 and F2 were identified using BEDTools (Quinlan and Hall 2010). Dindel was also run on H99 mappings and F0 and F2 predictions overlapping with these were excluded. Unique predictions in F0 and F2 were examined manually. Validation was performed using primers listed (Table S4). Identification of synteny was performed using Mauve (Pareek *et al.* 2011). SNVs were identified using GATK UnifiedGenotyper with filters as described and CLC Genomics Workbench with default settings. Centromere regions were excluded from analysis. Comparisons between strains were performed using BEDtools (Quinlan and Hall 2010) and CLC Genomics Gateway beta. Figure 4 was constructed using Circos (Krzywinski *et al.* 2009). Copy number variation was detected using FREEC (Boeva *et al.* 2011) employing a 1,001 bp windows with a 100 bp step size and using H99 as control. Altered copy regions were then manually examined in Integrative Genomics Viewer 1.5 (Robinson *et al.* 2011), filtering out regions identified within telomeres and centromeres due to their repetitive nature. Coverage at the chromosome and gene level was determined using GATK DepthOfCoverage and CLC Genomics Workbench. rDNA and mitochondrial genome copy number was calculated as described (James *et al.* 2009).

References

Albers, C. A., G. Lunter, D. G. MacArthur, G. McVean, W. H. Ouwehand *et al.*, 2011 Dindel: Accurate indel calls from short-read data. Genome Res. **21:** 961-973.

Altschul, S. F., W. Gish, W. Miller, E. W. Myers, and D. J. Lipman, 1990 Basic local alignment search tool. J. Mol. Biol. **215:** 403-410.

Benson, G., 1999 Tandem repeats finder: a program to analyze DNA sequences. Nucleic Acids Res. **27:** 573-580.

Boeva, V., A. Zinovyev, K. Bleakley, J. P. Vert, I. Janoueix-Lerosey *et al.*, 2011 Control-free calling of copy number alterations in deep-sequencing data using GC-content normalization. Bioinformatics **27:** 268-269.

Brenner, S., 1974 Genetics of *Caenorhabditis elegans.* Genetics **77:** 71-94.

Chaskes, S., and R. L. Tyndall, 1975 Pigment production of *Cryptococcus neoformans* from *para*-diphenols and *ortho*-diphenols: Effect of nitrogen source. J. Clin. Microbiol. **1:** 509-514.

Chen, K., J. W. Wallis, M. D. McLellan, D. E. Larson, J. M. Kalicki *et al.*, 2009 BreakDancer: an algorithm for high-resolution mapping of genomic structural variation. Nat Meth **6:** 677-681.

Chen, S. C. A., M. Muller, J. Z. Zhou, L. C. Wright, and T. C. Sorrell, 1997 Phospholipase activity in *Cryptococcus neoformans*: A new virulence factor? J. Infect. Dis. **175:** 414-420.

Christensen, W. B., 1946 Urea decomposition as a means of differentiating *Proteus* and paracolon cultures from each other and from *Salmonella* and *Shigella* types. J. Bacteriol. **52:** 461-466.

DePristo, M., E. Banks, R. Poplin, K. Garimella, J. Maguire *et al.*, 2011 A framework for variation discovery and genotyping using next-generation DNA sequencing data. Nat. Genet. **43:** 491-498.

Ellinghaus, D., S. Kurtz, and U. Willhoeft, 2008 LTRharvest, an efficient and flexible software for de novo detection of LTR retrotransposons. BMC Bioinformatics **9:** 18. doi: 10.1186/1471-2105-9-18.

Ghannoum, M. A., A. S. Ibrahim, Y. Fu, M. C. Shafiq, J. E. Edwards *et al.*, 1992 Susceptibility testing of *Cryptococcus neoformans*: A microdilution technique. J. Clin. Microbiol. **30:** 2881-2886.

James, S. A., M. J. T. O'Kelly, D. M. Carter, R. P. Davey, A. van Oudenaarden *et al.*, 2009 Repetitive sequence variation and dynamics in the ribosomal DNA array of *Saccharomyces cerevisiae* as revealed by whole-genome resequencing. Genome Res. **19:** 626-635.

Krzywinski, M., J. Schein, I. Birol, J. Connors, R. Gascoyne *et al.*, 2009 Circos: An information aesthetic for comparative genomics. Genome Res. **19:** 1639-1645.

Li, H., and R. Durbin, 2009 Fast and accurate short read alignment with Burrows-Wheeler transform. Bioinformatics **25:** 1754-1760.

Li, H., B. Handsaker, A. Wysoker, T. Fennell, J. Ruan *et al.*, 2009 The Sequence Alignment/Map format and SAMtools. Bioinformatics **25:** 2078-2079.

Li, R., Y. Li, K. Kristiansen, and J. Wang, 2008 SOAP: short oligonucleotide alignment program. Bioinformatics **24:** 713-714.

McKenna, A., M. Hanna, E. Banks, A. Sivachenko, K. Cibulskis *et al.*, 2010 The Genome Analysis Toolkit: a MapReduce framework for analyzing next-generation DNA sequencing data. Genome Res. **20:** 1297-1303.

Pareek, C. S., R. Smoczynski, and A. Tretyn, 2011 Sequencing technologies and genome sequencing. J. Appl. Genetics **52:** 413-435.

Quinlan, A. R., and I. M. Hall, 2010 BEDTools: A flexible suite of utilities for comparing genomic features. Bioinformatics **26:** 841-842.

Robinson, J. T., H. Thorvaldsdottir, W. Winckler, M. Guttman, E. S. Lander *et al.*, 2011 Integrative genomics viewer. Nat. Biotechnol. **29:** 24-26.

Smit, A. F. A., R. Hubley, and P. Green, 1996-2010 RepeatMasker Open-3.0.

Sobreira, T. J. P., A. M. Durham, and A. Gruber, 2006 TRAP: automated classification, quantification and annotation of tandemly repeated sequences. Bioinformatics **22:** 361-362.

Wang, J. M., C. G. Mullighan, J. Easton, S. Roberts, S. L. Heatley *et al.*, 2011 CREST maps somatic structural variation in cancer genomes with base-pair resolution. Nat. Methods **8:** 652-654.

Zaragoza, O., B. C. Fries, and A. Casadevall, 2003 Induction of capsule growth in *Cryptococcus neoformans* by mammalian serum and CO_2. Infect. Immun. **71:** 6155-6164.

Table S1A Indels identified between H99 and F0 and F2 within genes with functional annotation

Chr	H99	F0 & F2	Gene	Gene function	Effect of mutation on protein
**1**	T	TCCCACA	CNAG_00545	IDN3-B	Insertion of PH in 5x string at 259 (of 1,933)
**1**	T	TGGTTCA	CNAG_00619	tubulin folding cofactor C	Insertion of EP in 4x string at 224 (of 356)
**3**	GTTC	G	CNAG_02824	uncharacterized ACR, COG1565	Deletion of R11 (of 543)
**3**	GCAT	G	CNAG_02796	phospho-2-dehydro-3-deoxyheptonate aldolase	Deletion of S in 7x string at 281 (of 544)
**3**	AGA	AC	CNAG_07527	α-1,6-mannosyl transferase	H232E, truncation at 250 (of 518)
**3**	A	AAGAAGC	CNAG_07580	CAMK/CAMKL/MARK protein kinase	Insertion of AS in 3x string at 513 (of 1,172)
**3**	GATCGTC	G	CNAG_06888	cytoplasmic protein	Deletion of SS in 5x string at 414 (1,037)
**3**	AG	A	CNAG_06888	cytoplasmic protein	G1024A (of 1,037)
**3**	A	ACAAGCC	CNAG_06920	ubiquitin-specific protease	Insertion of AQ in 3x string at 276 (of 1,109)
**3**	GAGA	G	CNAG_06920	ubiquitin-specific protease	Deletion of K501 (of 1,109)
**3**	C	CCTT	CNAG_06927	peptidyl-prolyl cis-trans isomerase	Insertion of E480 (of 513)
**4**	A	ATGATGAAG G	CNAG_05096	histone deacetylase 3	Insertion of SSP151 (of 490)
**4**	G	GGTT	CNAG_05138	exo-β-1,3-glucanase	Insertion of N37 (of 785)
**4**	C	CCAGAAA	CNAG_05248	clathrin binding protein	Insertion of KQ738 (of 751)
**4**	G	GT	CNAG_05274	STE/STE20/YSK protein kinase	G727V, truncation at 727 (of 765)
**5**	TA	T	CNAG_00974	beta-lactamase domain-containing protein	C188Stop (of 300)
**7**	G	GATGGA	CNAG_06533	ATP-dependent permease	Insertion of NGNG in 2x string at 648 (of 1,051)
**7**	AAAGAGACA TACGT	A	CNAG_06556	oxidoreductase	Q242R, truncation at 260 (of 323)
**7**	TC	T	CNAG_06623	*myo*-inositol oxygenase	S124L, truncation at 151 (of 315)
**8**	TCCCGCCACC	T	CNAG_03189	DIL and ankyrin domain-containing protein	G1093D (of 1,105)
**8**	TGTC	T	CNAG_03321	vacuolar protein sorting-associated protein 9	Deletion of T in 7x string at 131 (of 714)
**8**	TG	T	CNAG_03339	biotin transporter	H439P, truncation at 458 (of 503)
**8**	AG	ATA	CNAG_03519	cytoplasmic protein	Q453H, truncation at 459 (of 502)

Chr	H99	F0 & F2	Gene	Gene function	Effect of mutation on protein
**8**	TC	T	CNAG_03533	JmjC domain-containing histone demethylation protein 1	A505G, truncation at 557 (of 874)
**9**	A	AACC	CNAG_04213	signal transducer	Insertion of G152 (of 978)
**9**	A	AAGC	CNAG_04380	peptidase	Insertion of S in 6x string at 250 (of 688)
**10**	T	TAGA	CNAG_04792	phosphatidyl serine decarboxylase	Insertion of E in 4x string at 340 (of 1,230)
**10**	G	GA	CNAG_04696	DNA clamp loader	S438F, truncation at 444 (of 760)
**11**	A	ACGGTGATG G	CNAG_01809	small nuclear ribonucleoprotein hPrp3	Insertion of GGD501 (of 590)
**11**	TCAACTCCAA CTC	T	CNAG_02000	short-chain dehydrogenase	Deletion of SNSN in 3x string at 40 (of 358)
**11**	AG	A	CNAG_02016	DUF1479 domain-containing protein	V158L, truncation at 196 (of 493)
**12**	ACAG	A	CNAG_06193	CMGC/RCK protein kinase	Deletion of Q in 5x string at 94 (of 1,262)
**13**	A	AC	CNAG_06499	phosphatidic acid phosphatase type 2 domain containing 1 protein	C252V, truncation at 297 (of 382)

Table S1B Structural variation identified between H99 and F0 and F2

Chr	Type	Size (bp)	Gene	Gene name
**1**	Deletion	3,407	CNAG_00549	hypothetical protein
**2**	Deletion	47	CNAG_03991	integral membrane protein
**2**	Insertion	656	CNAG_04003 (intron)	pumilio 2
**3**	Deletion	383		
**3**	Deletion	3,409	CNAG_02711	hypothetical protein
**3**	Deletion	150	CNAG_02706, CNAG_02707	hypothetical proteins
**4**	Deletion	539		
**4**	Deletion	557	CNAG_05266 (intron)	membrane protein
**5**	Deletion	302	CNAG_06863	hypothetical protein
**5**	Deletion	716	CNAG_06843	hypothetical protein
**5**	Deletion	7,459	CNAG_01367, CNAG_01368	hypothetical proteins
**5**	Deletion	475		
**5**	Deletion	1,413	CNAG_01032	hypothetical protein
**6**	Deletion	260		
**6**	Insertion	664		
**7**	Tandem duplication	643	CNAG_06557	membrane protein
**7**	Deletion	323		
**7**	Insertion/ duplication	7,464	CNAG_00813/01368, CNAG_00814/01367	hypothetical proteins
**7**	Deletion	7,155	CNAG_05911, CNAG_05910	hypothetical proteins
**7**	Insertion/ duplication	9,600	CNAG_07313, CNAG_00127, CNAG_00128	hypothetical proteins
**8**	Insertion	1,033	CNAG_07707	glycoside hydrolase family 3 domain-containing protein
**8**	Insertion	915	CNAG_03195	hypothetical protein
**8**	Deletion	114		
**8**	Deletion	7,132	CNAG_03477, CNAG_03478	hypothetical proteins
**8**	Deletion	99		
**10**	Deletion	556	CNAG_07836 (intron)	NAD binding dehydrogenase family protein
**10**	Insertion	402		
**10**	Insertion	432	CNAG_04901	hypothetical protein
**10**	Insertion	664		
**10**	Deletion	5,210		
**10**	Deletion	246		
**11**	Deletion	7,427	CNAG_07613, CNAG_01968	hypothetical proteins
**13**	Deletion	77	CNAG_06336	glucan 1,3 beta-glucosidase
**13**	Deletion	15		
**14**	Deletion	591		

**14**	Deletion	165		
**14**	Deletion	248		
**14**	Deletion	85		
**14**	Deletion	592	CNAG_07874	sugar transporter
**14**	Deletion	11	CNAG_05643	hypothetical protein

Table S2 Genes on the left arm of chromosome 12

Strand	Locus	Length	Start	Stop	Name
-	CNAG_06939	1,938	204	2,141	conserved hypothetical protein
-	CNAG_06938	432	4,923	5,354	conserved hypothetical protein
-	CNAG_07026	670	7,207	7,876	conserved hypothetical protein
+	CNAG_07894	287	9,950	10,236	conserved hypothetical protein
-	CNAG_05986	1,052	12,447	13,498	conserved hypothetical protein
-	CNAG_05987	1,083	13,917	14,999	conserved hypothetical protein
-	CNAG_05988	580	15,532	16,111	conserved hypothetical protein
+	CNAG_05989	1,900	17,145	19,044	predicted protein
-	CNAG_05990	2,564	19,424	21,987	predicted protein
+	CNAG_05991	1,886	22,684	24,569	glycosyl hydrolase family 88
+	CNAG_05992	2,338	25,550	27,887	conserved hypothetical protein
-	CNAG_05993	2,668	27,970	30,637	transmembrane transporter Liz1
+	CNAG_05994	2,256	31,431	33,686	multidrug transporter
-	CNAG_05995	2,215	34,421	36,635	conserved hypothetical protein
+	CNAG_05996	3,075	38,318	41,392	amino acid transporter
-	CNAG_05997	849	42,493	43,341	conserved hypothetical protein
+	CNAG_05998	1,353	43,606	44,958	rho GTPase
-	CNAG_05999	974	45,350	46,323	peptide alpha-N-acetyltransferase
+	CNAG_06000	1,986	46,804	48,789	glycoprotein
+	CNAG_06001	1,923	49,240	51,162	phosphomevalonate kinase
-	CNAG_06002	1,752	51,476	53,227	tRNA (5-methylaminomethyl-2-thiouridylate)-methyltransferase
+	CNAG_06003	912	54,177	55,088	conserved hypothetical protein
-	CNAG_06004	1,440	55,430	56,869	conserved hypothetical protein
+	CNAG_06005	3,065	57,220	60,284	conserved hypothetical protein
-	CNAG_06006	1,542	60,590	62,131	conserved hypothetical protein
+	CNAG_06007	1,057	62,416	63,472	hypothetical protein
+	CNAG_06008	1,378	63,801	65,178	asparaginase
+	CNAG_06009	2,427	65,715	68,141	cyclohydrolase
-	CNAG_07895	402	70,290	70,691	predicted protein
-	CNAG_06010	2,541	71,423	73,963	fatty aldehyde dehydrogenase
+	CNAG_06011	2,097	74,927	77,023	conserved hypothetical protein
-	CNAG_06012	1,508	77,134	78,641	NADH-cytochrome b5 reductase
+	CNAG_06013	2,116	78,850	80,965	vacuolar protein
-	CNAG_06014	891	81,022	81,912	predicted protein
-	CNAG_06015	651	81,943	82,593	predicted protein
-	CNAG_07896	702	83,434	84,135	predicted protein
-	CNAG_06016	2,025	85,492	87,516	capsule associated protein 6
+	CNAG_06017	573	88,620	89,192	predicted protein
+	CNAG_06018	2,421	89,818	92,238	aldehyde dehydrogenase
+	CNAG_06019	5,653	93,866	99,518	conserved hypothetical protein

Strand	Locus	Length	Start	Stop	Name
-	CNAG_06020	1,738	100,066	101,803	conserved hypothetical protein
+	CNAG_06021	2,681	103,158	105,838	rab GTPase activator
-	CNAG_06022	1,429	105,986	107,414	trans-aconitate 3-methyltransferase
+	CNAG_06023	678	107,717	108,394	conserved hypothetical protein
-	CNAG_06024	1,294	108,487	109,780	peroxin 14
+	CNAG_06026	2,187	110,990	113,176	aspartate transaminase
-	CNAG_06027	2,064	112,960	115,023	aryl-alcohol dehydrogenase
-	CNAG_06028	1,242	116,243	117,484	conserved hypothetical protein
+	CNAG_06029	741	117,802	118,542	conserved hypothetical protein
-	CNAG_06030	1,968	118,609	120,576	conserved hypothetical protein
+	CNAG_06031	1,177	122,495	123,671	β-glucan synthesis-associated protein
+	CNAG_06032	948	124,300	125,247	ADP-ribosylation factor-like protein 2
-	CNAG_06033	1,661	125,368	127,028	pfkB family carbohydrate kinase superfamily
+	CNAG_06034	2,158	131,492	133,649	allantoin permease
-	CNAG_06035	1,814	134,163	135,976	alcohol dehydrogenase
+	CNAG_06036	2,651	136,817	139,467	aminotransferase LolT-1

Table S3 Statistical parameters of multivariate PCA models of metabolomic data

Model	N	k	A	R^2X	Q^2	Scaling	Comparison	Figure
**M1**	34	9151	6	0.919	0.85	Pareto	All samples	3A
**M2**	11	9151	3	0.876	0.698	Pareto	F0 *vs.* F2 in YNB medium	3B & C
**M3**	11	9151	2	0.936	0.889	Centre	F0 *vs.* F2 in YNB medium	3B & C
**M4**	12	9151	3	0.785	0.458	Pareto	F0 *vs.* F2 in YPD medium	S5A & B
**M5**	12	9151	5	0.98	0.866	Centre	F0 *vs.* F2 in YPD medium	S5A & B
**M6**	12	9151	4	0.916	0.826	Pareto	F2 in YNB *vs.* YPD medium	S5C & D
**M7**	12	9151	3	0.986	0.961	Centre	F2 in YNB *vs.* YPD medium	S5C & D
**M8**	11	9151	3	0.848	0.545	Pareto	F0 in YNB *vs.* YPD medium	S5E & F
**M9**	11	9151	2	0.838	0.567	Centre	F0 in YNB *vs.* YPD medium	S5E & F

N= number of samples, k= number of x-variables (buckets), A= number of principal components in the model, R^2X=sum of squares of all x-variables explained by the model, Q^2= cumulative cross-validated R^2.

Table S4 Primers used in this study

Primer ID	Sequence
**UQ623**	CAGGTCAAGGAAAGGCAACAG
**UQ624**	TGCCCTGAAGTTGGTTAGACT
**UQ702**	ACAAAATCCACGCATACAGAA
**UQ703**	CGCCACCATCCAAACGTCAAA
**UQ704**	CAATCCCTTTGCCATCTGAAC
**UQ705**	GGTCAACCGCCTGTTTCTAAT
**UQ706**	CCCATATTATTCCCAATCTGA
**UQ707**	CCTGCATTGTTCGCCTCTCTA
**UQ708**	GCGTGGCCGACCTTACCTCAG
**UQ709**	CCAATACACCAACAGCGTGAC
**UQ801**	GCGAAAGGGTGAGAAGTGAAA
**UQ802**	CACTAAAGGGCGGCCATTAAA
**UQ1836**	AGCTCTACCCACTACCGAATC
**UQ1837**	CGACGATGACTCCCACTACCA
**UQ1838**	CACCTGGAGAAGTTCGCTGAG
**UQ1839**	GGCTGAACATTGTCGCTTATT
**UQ1840**	TGTGGGATGAATGTAAGTGCT
**UQ1842**	GGACTTTGCTTCGGATGATCT
**UQ1843**	CACATGCTCGCTTAGTTGC
**UQ1844**	TATCAGCGAAATACGACAAGG
**UQ1845**	GCTAGTCACAGGTCGTCGGT
**UQ1846**	GCACTTCCAACGCAGGTT
**UQ1847**	CGCTCTTGCTTTGACCAACTC
**UQ1848**	TTGCAATGACAAAAGGCTTAT
**UQ1849**	TGCTCTCCACCACTTCGATT
**UQ1850**	CGGCAGGTTCGATATGG
**UQ1851**	TGTTGTTGTTGCGTAGTCGTC
**UQ1852**	CAGGCTAGTGAGTCGGCTACA
**UQ1853**	CGGGGAATTCGATCATC
**UQ1854**	CTCCTCGCACGGTTCTC
**UQ1855**	TGGAGGCTCGTGACATATGAA
**UQ1856**	AGCCATTTCTTTCAGTCGAGC
**UQ1857**	GGGGTTTGATTGGTGCAAG
**UQ1858**	CCTCCGTCCTCCGAGTCGTT
**UQ1859**	GGAAGAAATTGGGTAAGCC
**UQ1860**	AAACATGTCTACCGTTGACCC
**UQ1861**	CGGAGGTGAAATAGAACGC
**UQ1862**	CCCTTATCCAAGATTCCGTGA
**UQ1865**	CGGCATAGAGAACGGGAAG
**UQ1866**	ACGCTTTCAGACCCTCGTTCT
**UQ1869**	TCTTGAATCTGCGAGCGTGAA
**UQ1870**	CACCGAAAGAAGCGTCTAAAC
**UQ1873**	CTTCTCCATCCATGCTGACA
**UQ1874**	CGGCCTCATGATGTTAAGT
**UQ1877**	CGGCATAGAGAACGGGAAG
**UQ1878**	GTACCGTAGTGCGCCTGA
**UQ1881**	AGCCGACGACGAAGTTCA
**UQ1882**	CACAAAGACCTTGCCATTATG
**UQ1885**	CAAAAAAAGATTCTCCCTCC
**UQ1886**	CCCTGAACCAGCCGTCT
**UQ1889**	GTATTGGAGGCACGGCAGA

Primer ID	Sequence
**UQ1894**	GGCGATGGTAAAGATGATAGC
**UQ1897**	CTCTCAAGAAGACGCTGACTT
**UQ1898**	GGCCTGGTGTCGGTATTC
**UQ1899**	CGCATCCTTCAATGACATTGG
**UQ1902**	GTCATCCGCAACTCATATCA
**UQ1903**	TGCTGTAGCGTCTGCGTGTGA
**UQ1904**	TCAAGCGAACTTAAAGGGTAA
**UQ1905**	AGGCGTTGGACTTCGAC
**UQ1906**	CCATACTTTTTGCCCGTTTAC
**UQ1907**	GGCCCAGGAGGTGATCAATTT
**UQ1908**	CGCGCCTTTCCTATCTC
**UQ1909**	GATGTGGGAATGACGGGAGTC
**UQ1910**	GAAATGTTCCCGTGTCGTCAT
**UQ1911**	CGAAATTGTTTGCCGATTG
**UQ1986**	GGAGGTGGACTGTATTGTGAG
**UQ1987**	CAATAATATCGTCTCGGGTGG
**UQ1990**	CGATGGATTTTAACCGTGACT
**UQ1991**	CCCGTATTTCAAACACTCTCA
**UQ1994**	CAGGGCACGAAAGGGACAGGT
**UQ1995**	GGGAAGTGATTGAGCATTTTT
**UQ1998**	CGACGGCTGAAGAACAAGGTG
**UQ1999**	ATCCGTAATCATTGCCAACAC
**UQ2000**	GCTCGCACTTCACCACTATTC
**UQ2001**	GTTGTGCTGGCTGCTGTTGTT
**UQ2004**	GCCCAAGGAAGGAAAGCTCAA
**UQ2005**	GGCTTGAGGCAAAAGACGACA
**UQ2006**	GGTGGACGAGAAGGATGAAAA
**UQ2007**	CGTCAGACCTAGCGTTTACTT
**UQ2008**	CTGCCAGTTATGAGCTGTCGG
**UQ2009**	TGCTTGTATGGTTGTCGCATT
**UQ2010**	TGGGGGACAGGGATGCACGGA
**UQ2011**	AAAGGCAAGTCACGTCGAAAA
**UQ2012**	AAGAAGTCGATCTCCCGCTCA
**UQ2013**	TAGGCTTCACTGACACACAAA
**UQ2014**	ATAAAAACCAGCCAGGTCTGC
**UQ2015**	CCTACTTCTTGTAGTAGCTGG
**UQ2016**	ACAGGGAAATGCTAAAAGTAT
**UQ2017**	AAGTTCGGGTACTCTGCTCTC
**UQ2018**	CACTTACTCGCCAGTCCACCA
**UQ2023**	CAGTGAGCCATCCTTACAGTC
**UQ2024**	AAACGATGATGCTGGAATTAC
**UQ2025**	CTGAGCATAATAATGGCGTCC
**UQ2026**	GAGATAAGGTCATCGCAAAAC
**UQ2072**	TGTACGAAGGCTATGAAGCTG
**UQ2073**	GATGGGCGTGTACTGTACTCT
**UQ2074**	TCACCGCTCGCTAAACCTGTT
**UQ2075**	CTAGGGTTTGGACGCACAACT
**UQ2076**	GGATACCAGCAATTCACTCCA
**UQ2087**	TCTCAGATCCTTCCCTTTGTC
**UQ2088**	CGCTCTCCAGCTCACATCCTCGCAGCTGAAACAGGATGT
**UQ2089**	CTACATCTCTTCCGTGTTAATACAGATAAACCGATGGTGGATGAGCTT
**UQ2090**	CTTTTTCGTTCAATCCTGCTT
**UQ2091**	ACATCCTGTTTCAGCTGCGAGGATGTGAGCTGGAGAGCG

Primer ID	Sequence
**UQ2092**	AAGCTCATCCACCATCGGTTTATCTGTATTAACACGGAAGAGATGTAG
**UQ2093**	CCGGAAGAGTGCTTGCAGATT
**UQ2094**	TTCGCCTCTGGACCGTGAATG
**UQ2095**	ACTTGAGCATTTGCGGGTGTG
**UQ2096**	TTTCCAGCACCAGCGTTCTTG
**UQ2097**	TGCTGACTGGGCCCTTGTCCT
**UQ2098**	GTGCGCATGAATTCGTGGACT
**UQ2099**	CAAACCCTTTCCCGGACGACT
**UQ2156**	CTAGGAATGAACATGGGAATG
**UQ2157**	TAGTTTGTAGGTTGACCGGTT
**UQ2158**	AGTGCACGAGGTTATTGAAGA
**UQ2159**	CAGAAAGATCGAGAGAAACAG
**UQ2160**	TAAATCTTGGTCGTTGGGGTC
**UQ2263**	AAATGCAGCGGAACCAAAATC
**UQ2264**	AGAGCTAAGTTAAATGGGGGG
