# Supplementary material for: Comparative Genomics of Serial Isolates of Cryptococcus neoformans Reveals Gene Associated With Carbon Utilization and Virulence
Source: G3 (Bethesda). 2013 Apr 1;3(4):675–86. doi: 10.1534/g3.113.005660 (PMC3618354; doi:10.1534/g3.113.005660)
Supplement: Supporting Information [file supp_g3.113.005660_FigureS1.pdf]

(A)

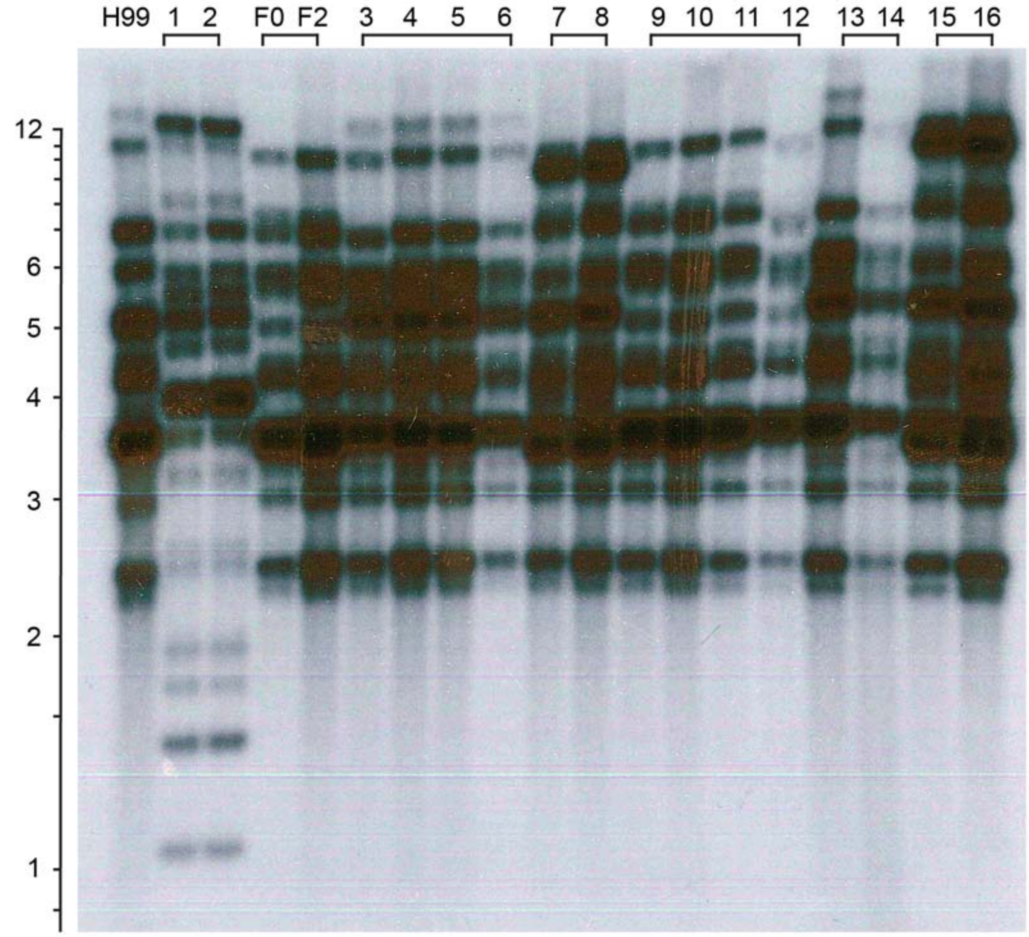

**(B)**

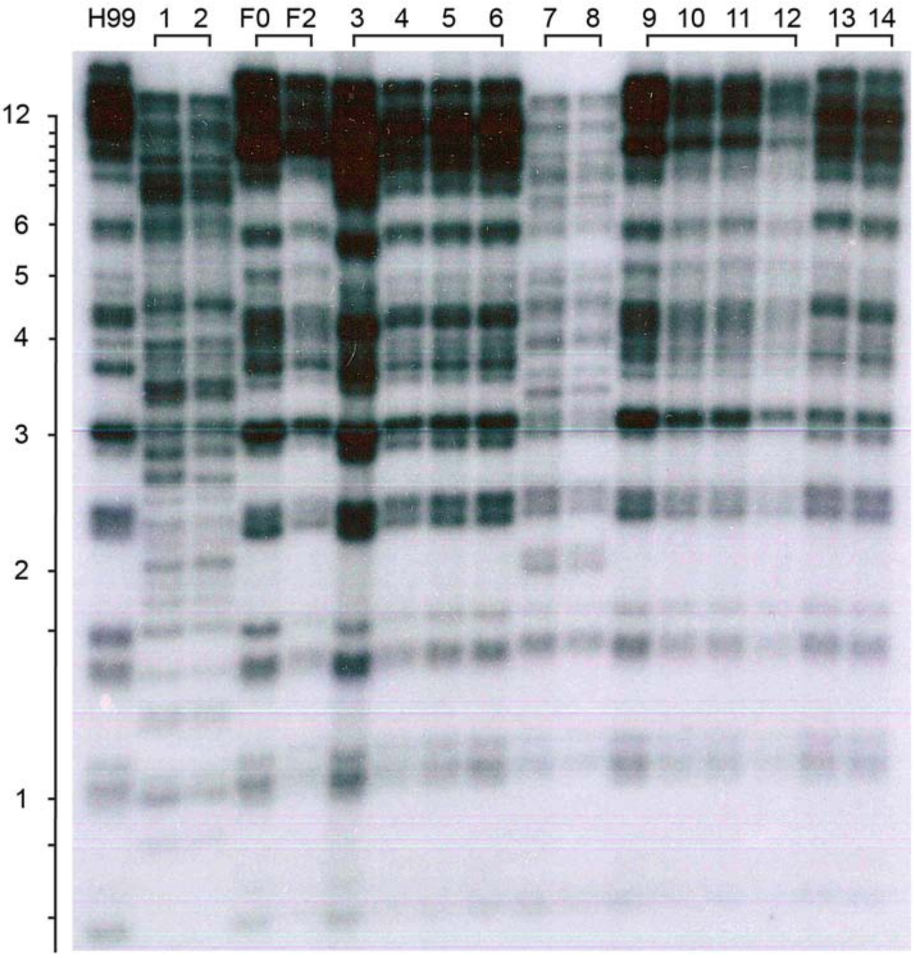

**(C)**

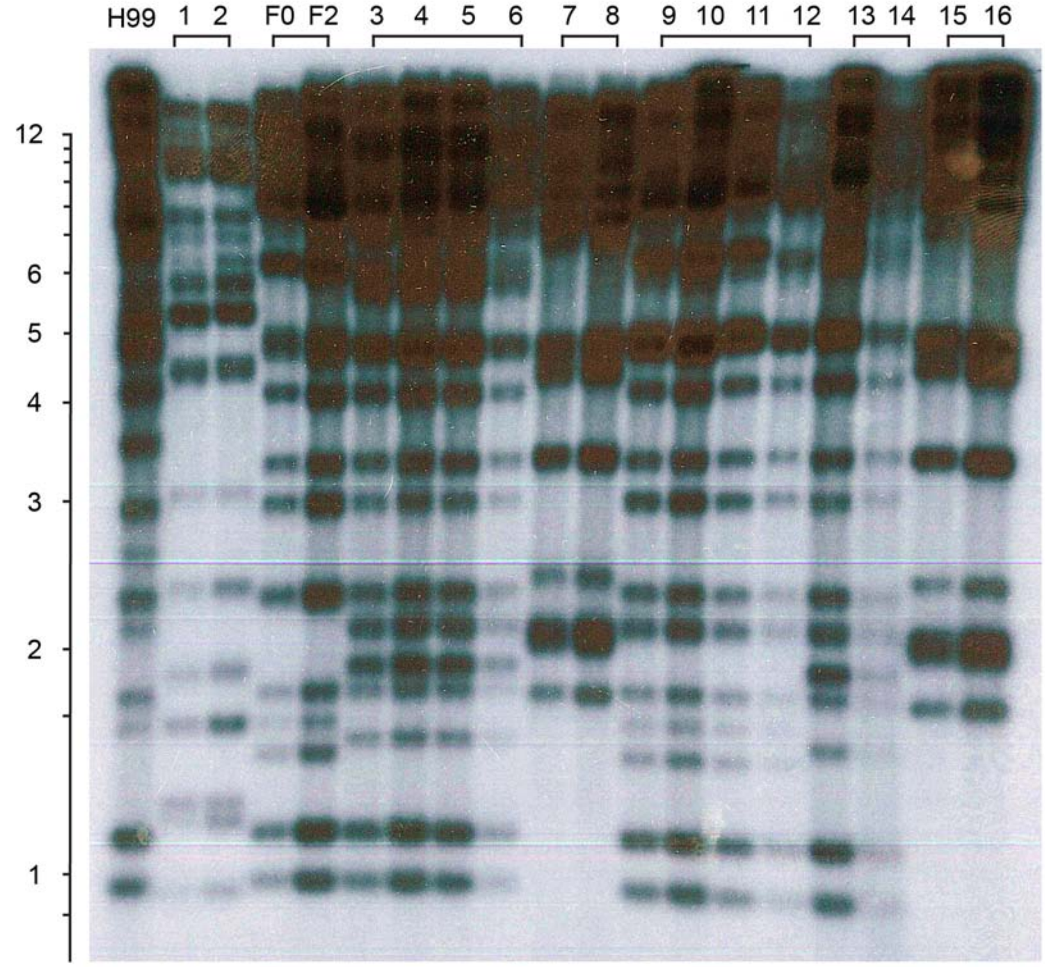

(D)

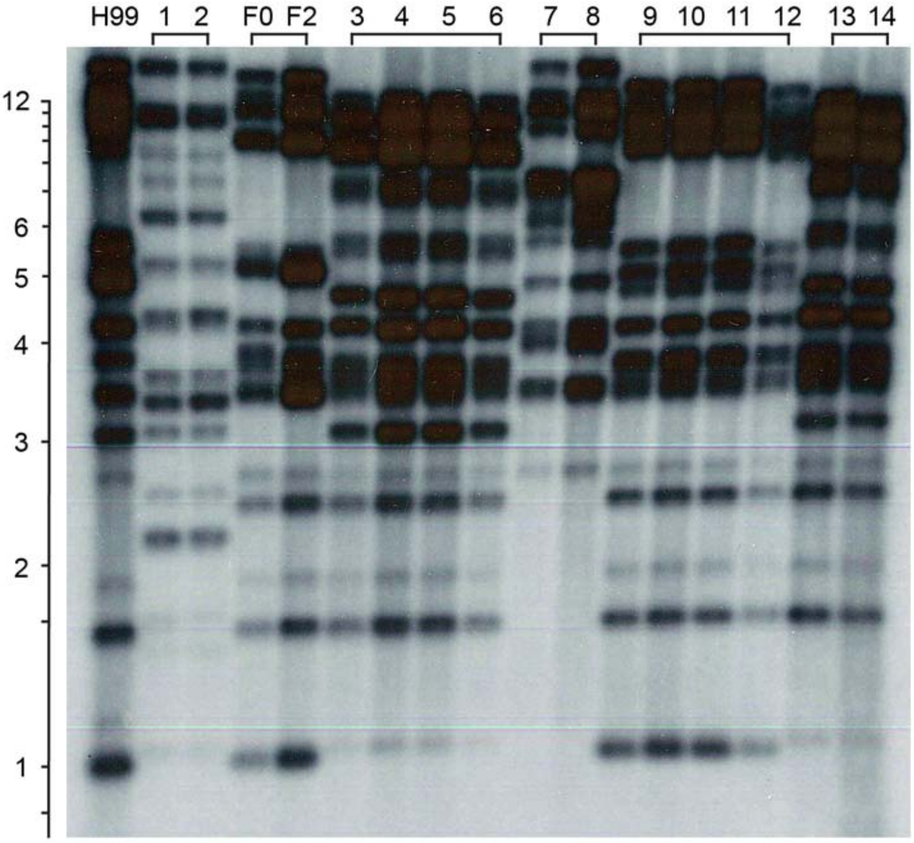

**(E)**

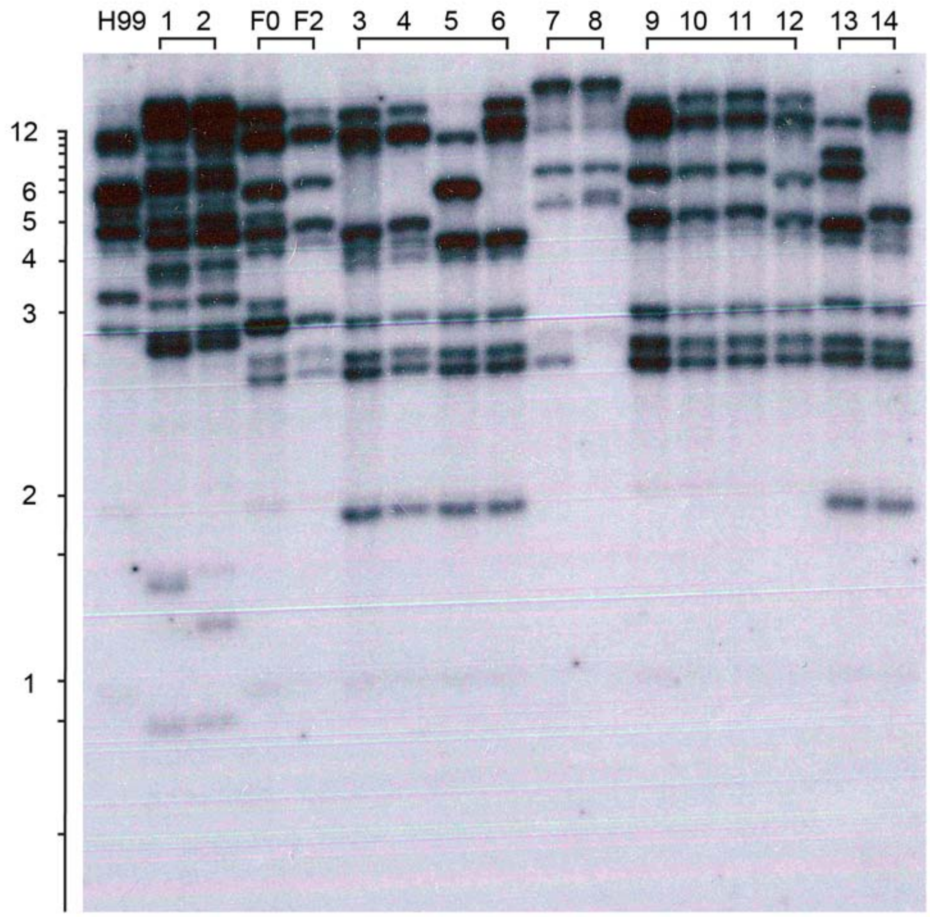

(F)

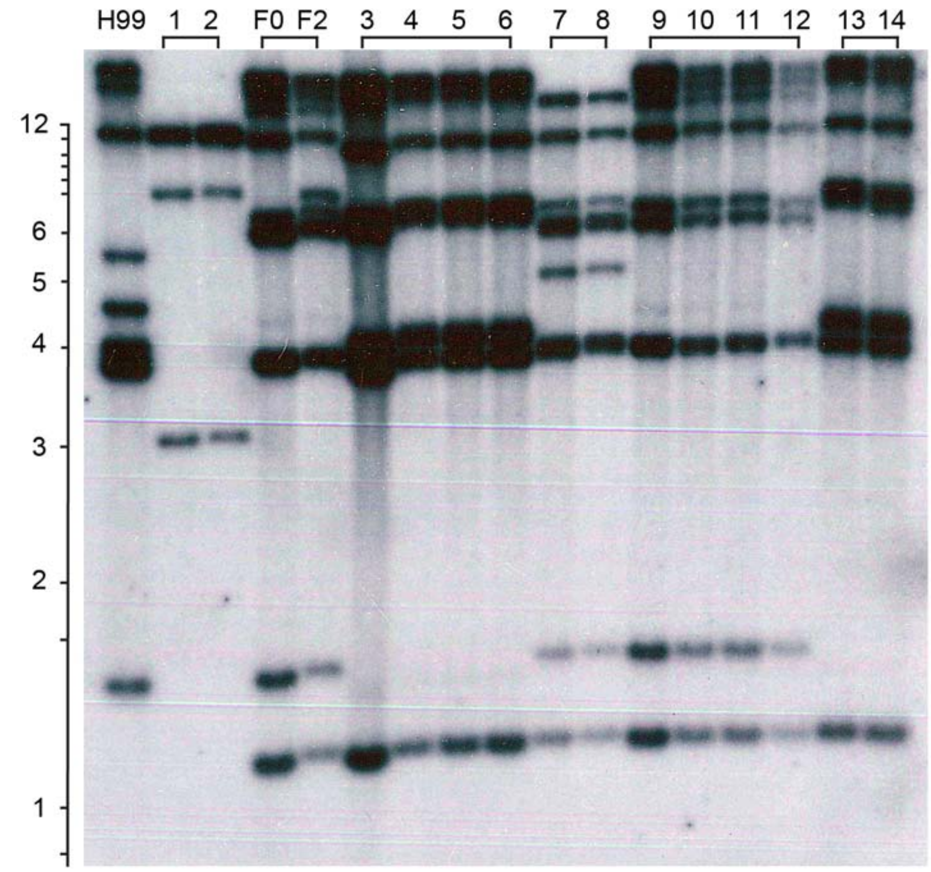


**FIGURE S1 Transposon profiles of F0 and F2 are the same for Cnirt2, Tcn1, Tcn2 and Tcn4 but different for Tcn6, a highly mobile transposon.** Southern blot analysis of serial isolates (sets marked with rectangles) probed with transposon fragments of **(A)** Cnirt2, **(B)** Tcn1, **(C)** Tcn2, **(D)** Tcn4, **(E)** Tcn6 internal region, **(F)** Tcn6 LTR. Strains 1 to 16 are serial clinical isolates that have not as yet been characterized.
