# Supplementary material for: Comparative Genomics of Serial Isolates of Cryptococcus neoformans Reveals Gene Associated With Carbon Utilization and Virulence
Source: G3 (Bethesda). 2013 Apr 1;3(4):675–86. doi: 10.1534/g3.113.005660 (PMC3618354; doi:10.1534/g3.113.005660)
Supplement: Supporting Information [file supp_g3.113.005660_FigureS2.pdf]

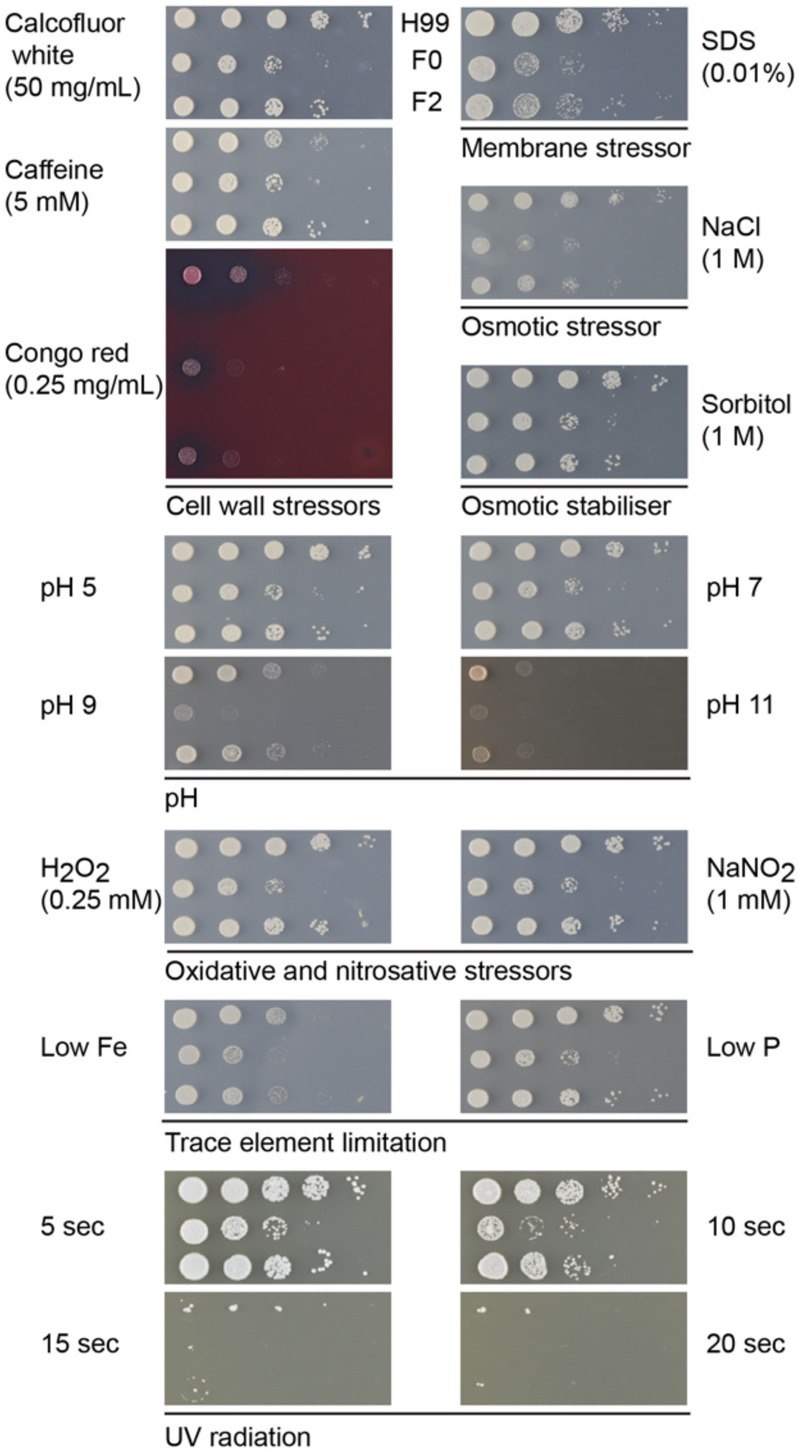

FIGURE S2 F0 and F2 exhibit similar responses to a variety of stresses. 10-fold serial dilutions of indicated strains were spotted onto minimal media supplemented with various stressors and incubated at 30˚ for 2-3 days. The growth defect of F0 is not exacerbated by growth on cell wall stressors. F0 and F2 show increased sensitivity to cell membrane and osmotic stress. No difference in growth is seen on the osmotic stabilizer sorbitol. Increased sensitivity to high pH (9 and 11) is seen in F0. No increased sensitivity is seen in either F0 or F2 under selected oxidative or nitrosative stressors or nutrient limitation. No increased sensitivity is seen in F0 or F2 when exposed to UV radiation.
