# Supplementary material for: Comparative Genomics of Serial Isolates of Cryptococcus neoformans Reveals Gene Associated With Carbon Utilization and Virulence
Source: G3 (Bethesda). 2013 Apr 1;3(4):675–86. doi: 10.1534/g3.113.005660 (PMC3618354; doi:10.1534/g3.113.005660)
Supplement: Supporting Information [file supp_g3.113.005660_FigureS3.pdf]

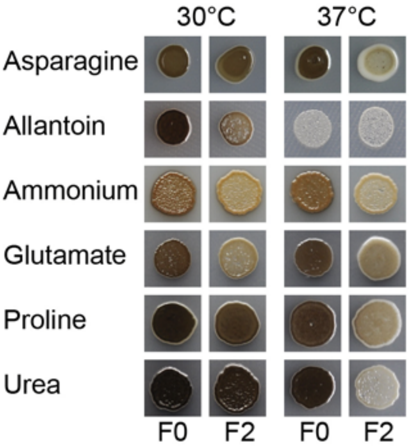

FIGURE S3 Melanin production in F2 is reduced on multiple nitrogen sources. When grown on L-DOPA containing Cryptococcus melanization media supplemented with various nitrogen sources, F2 exhibits a significant melanization defect at 37˚, also visible on many nitrogen sources at 30˚.
