# Supplementary material for: Comparative Genomics of Serial Isolates of Cryptococcus neoformans Reveals Gene Associated With Carbon Utilization and Virulence
Source: G3 (Bethesda). 2013 Apr 1;3(4):675–86. doi: 10.1534/g3.113.005660 (PMC3618354; doi:10.1534/g3.113.005660)
Supplement: Supporting Information [file supp_g3.113.005660_FigureS4.pdf]

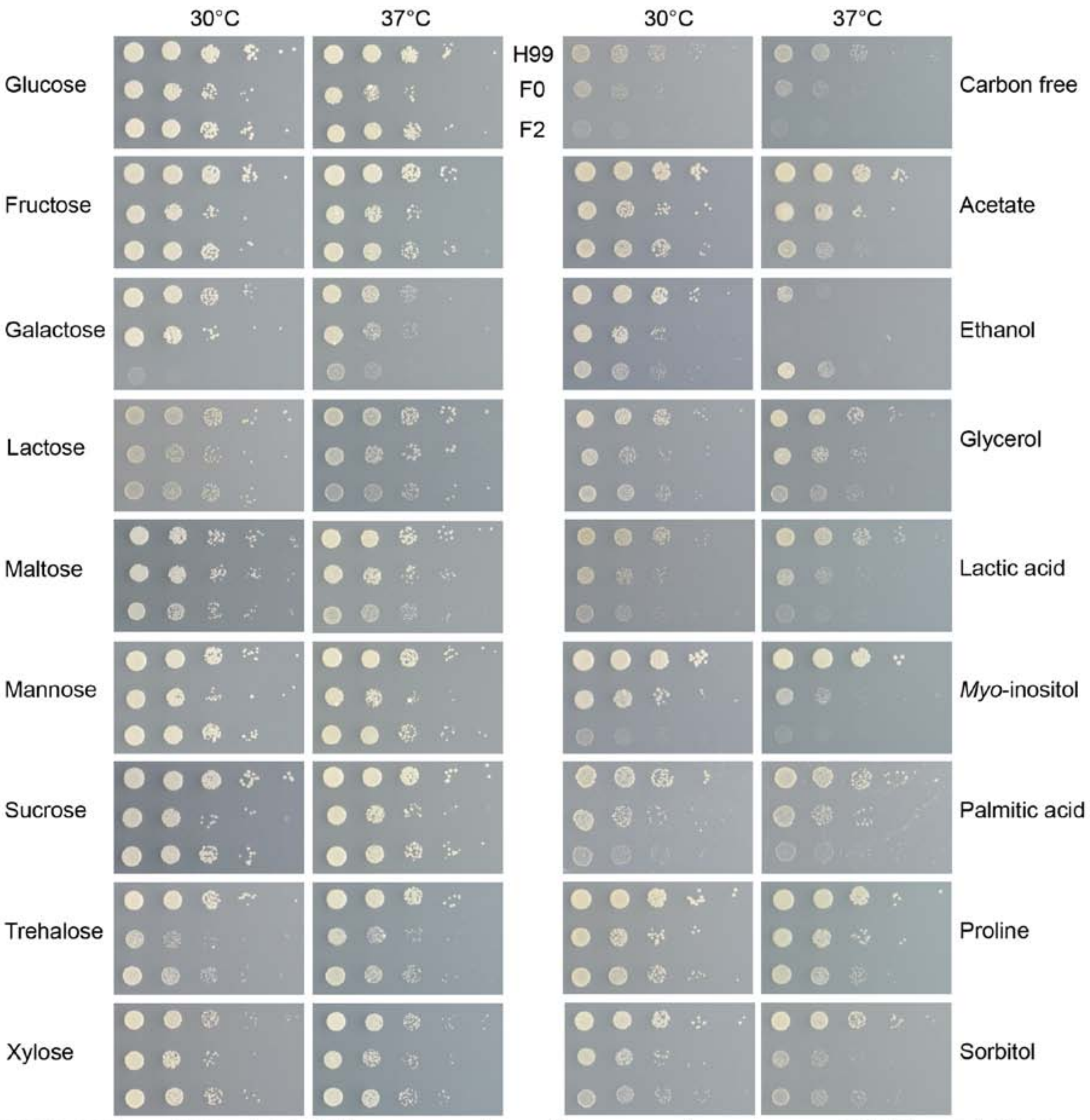

FIGURE S4 F0 and F2 exhibit different growth on alternate carbon sources. 10-fold serial dilutions of indicated strains were spotted onto minimal media supplemented with various carbon sources and incubated at 30 and 37° for 2 to 3 days.
