# Supplementary material for: Comparative Genomics of Serial Isolates of Cryptococcus neoformans Reveals Gene Associated With Carbon Utilization and Virulence
Source: G3 (Bethesda). 2013 Apr 1;3(4):675–86. doi: 10.1534/g3.113.005660 (PMC3618354; doi:10.1534/g3.113.005660)
Supplement: Supporting Information [file supp_g3.113.005660_FigureS5.pdf]

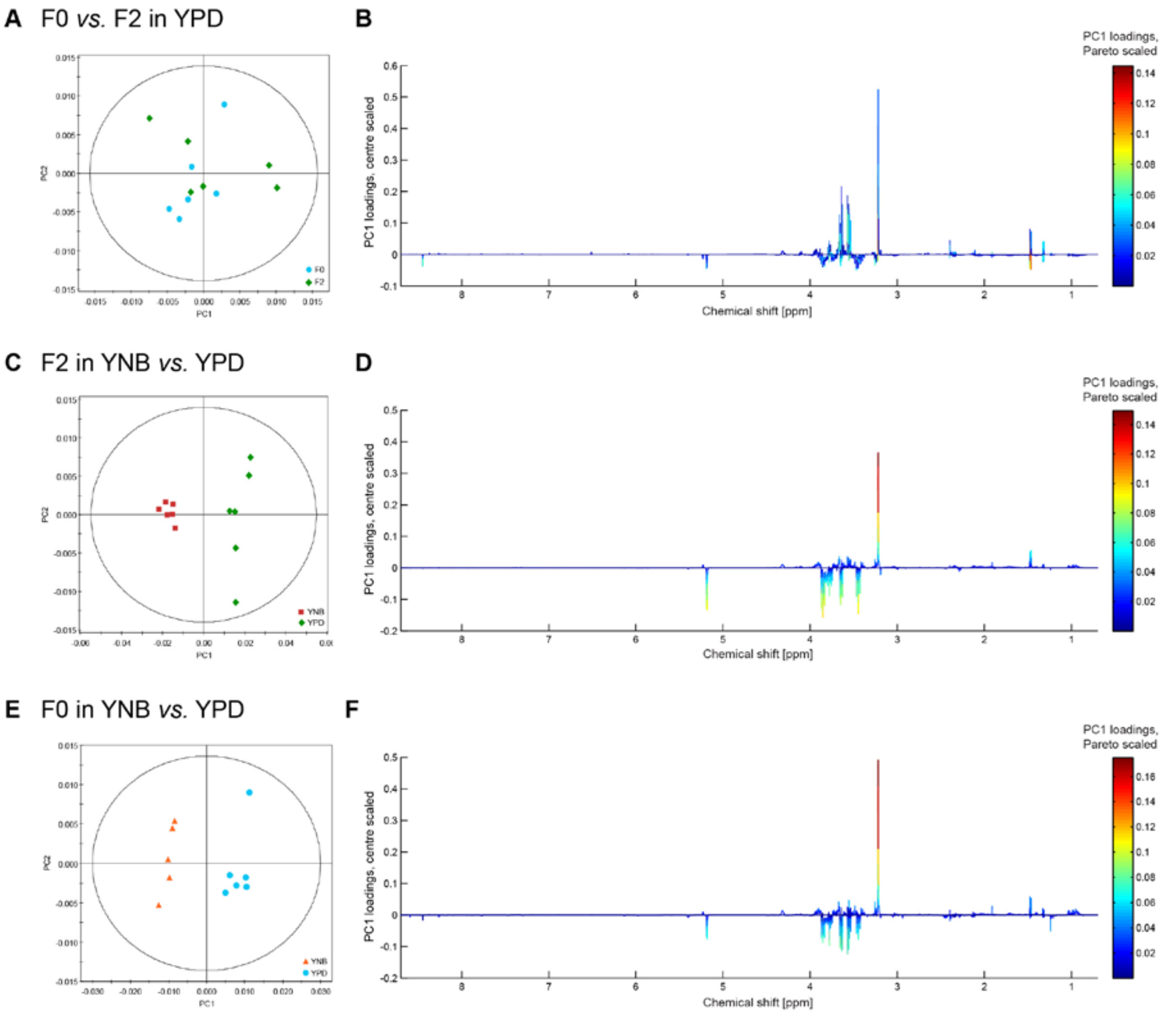

FIGURE S5 Pairwise comparisons of metabolic profiles with principal components analysis. (A) & **(B)** Comparison of F0 (cyan circles) with F2 (green diamonds) in YPD medium. **(C)** & **(D)** Comparison between F2 in YNB (red squares) and YPD (green diamonds) medium. **(E)** & **(F)** Comparison between F0 in YNB (orange triangles) and YPD (cyan circles) medium. **(A)**, **(C)** & **(E)**: Principal components analysis scores plots. Hotelling's 95% confidence range of the PCA model is indicated in the scores plots by an ellipse. **(B)**, **(D)** & **(F)**: Bivariate loadings plot with center scaled loadings coefficients shown as intensity, and Pareto scaled coefficients as heatmap.
