# Supplementary material for: Comparative Genomics of Serial Isolates of Cryptococcus neoformans Reveals Gene Associated With Carbon Utilization and Virulence
Source: G3 (Bethesda). 2013 Apr 1;3(4):675–86. doi: 10.1534/g3.113.005660 (PMC3618354; doi:10.1534/g3.113.005660)
Supplement: Supporting Information [file supp_g3.113.005660_FigureS6.pdf]

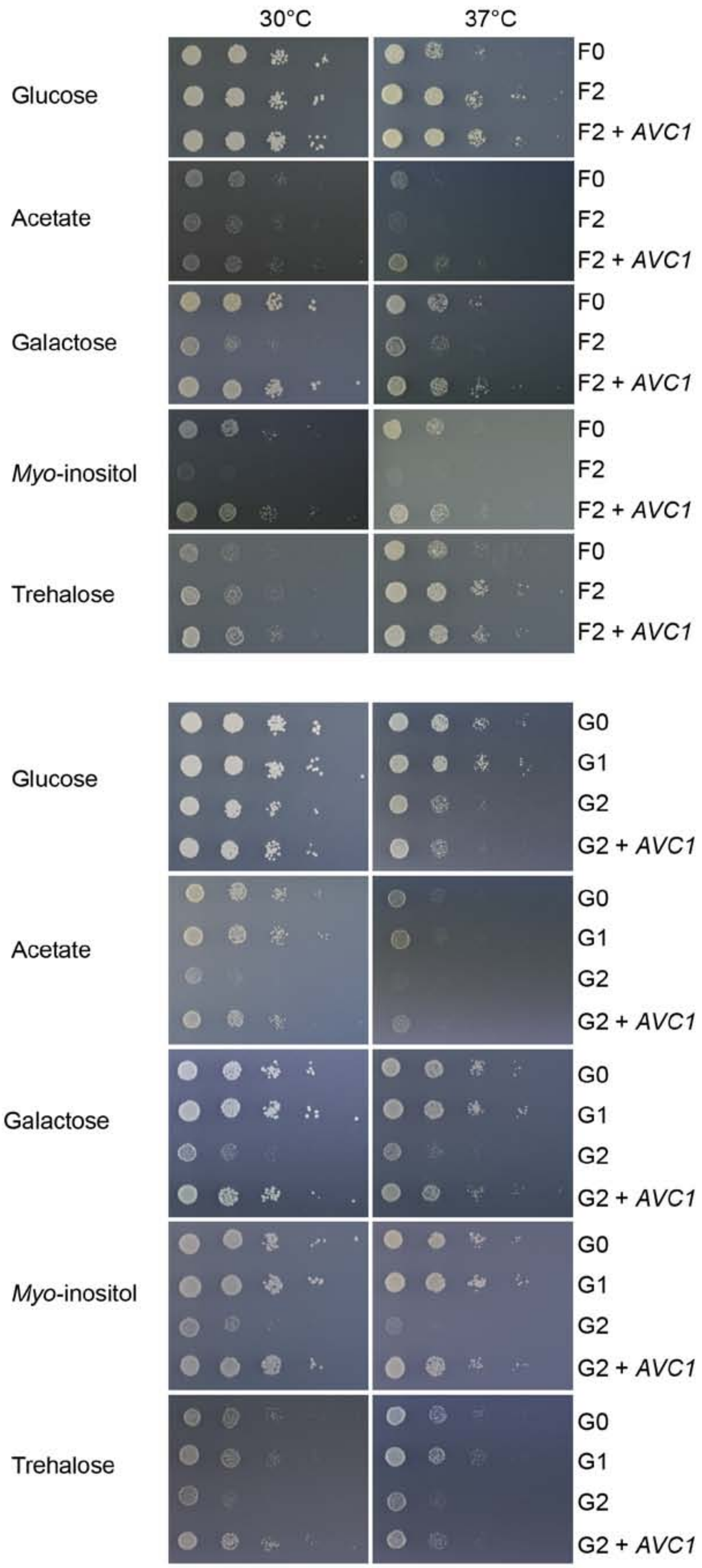

FIGURE S6 Reintroduction of ARID-containing gene *AVC1* rescues growth on alternate carbon sources. Growth assays on minimal media supplemented with various carbon sources.
