# Supplementary material for: Comparative Genomics of Serial Isolates of Cryptococcus neoformans Reveals Gene Associated With Carbon Utilization and Virulence
Source: G3 (Bethesda). 2013 Apr 1;3(4):675–86. doi: 10.1534/g3.113.005660 (PMC3618354; doi:10.1534/g3.113.005660)
Supplement: Supporting Information [file supp_g3.113.005660_FigureS8.pdf]

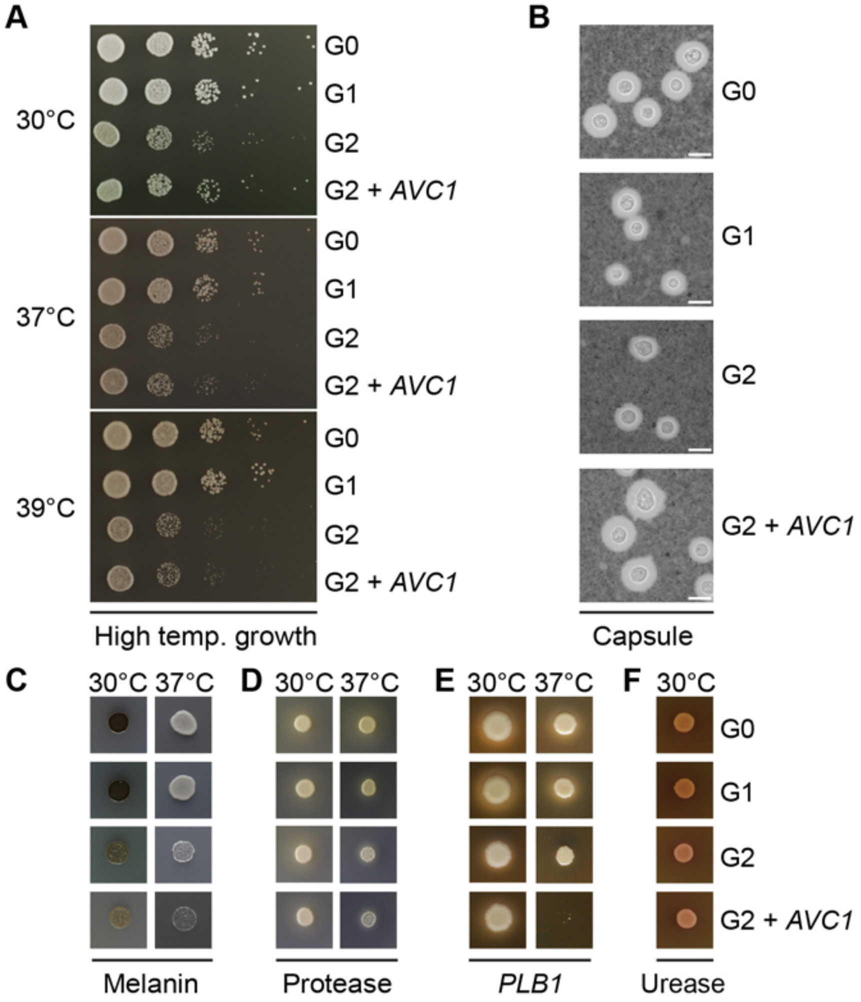

FIGURE S8 Reintroduction of ARID-containing *AVC1* rescues capsule production in G2. (A) Growth assays at 30˚, human body temperature of 37˚ and febrile body temperature of 39˚ on YPD; no change was observed following reintroduction of *AVC1*. **(B)** India ink staining under light microscopy reveals the capsule; capsule production in G2 is increased following reintroduction of *AVC1*. Scale bar is 10 μM. **(C)** Melanization on L-DOPA containing media continued to be inhibited in G2 following reintroduction of *AVC1*. **(D)**, **(E)** & **(F)** Comparable levels of protease, phospholipase and urease production were observed following reintroduction of *AVC1* when strains were grown on BSA, egg yolk and Christensen's agar, respectively. No growth was observed for G2 + *AVC1* on egg yolk agar at 37˚.
