# Supplementary material for: Comparative Genomics of Serial Isolates of Cryptococcus neoformans Reveals Gene Associated With Carbon Utilization and Virulence
Source: G3 (Bethesda). 2013 Apr 1;3(4):675–86. doi: 10.1534/g3.113.005660 (PMC3618354; doi:10.1534/g3.113.005660)
Supplement: Supporting Information [file supp_g3.113.005660_FigureS9.pdf]

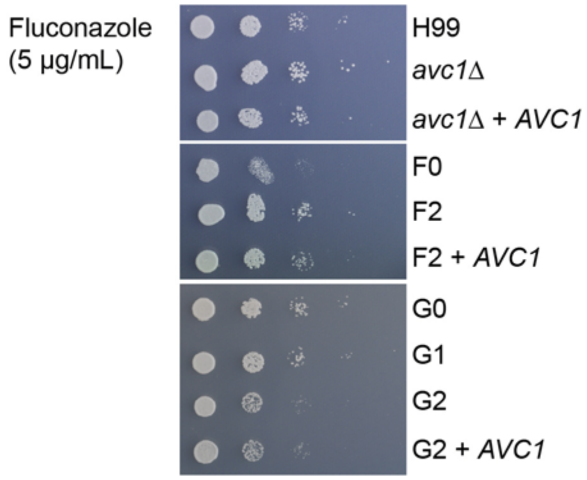

FIGURE S9 Deletion of *AVC1* increases resistance to fluconazole. Both *avc1*Δ and F2 exhibit increased growth on minimal media containing fluconazole (5 µg/mL) which is abolished when the gene is reintroduced. Growth is comparable between G2 and G2 + *AVC1*.
