# Supplementary material for: Comparative Genomics of Serial Isolates of Cryptococcus neoformans Reveals Gene Associated With Carbon Utilization and Virulence
Source: G3 (Bethesda). 2013 Apr 1;3(4):675–86. doi: 10.1534/g3.113.005660 (PMC3618354; doi:10.1534/g3.113.005660)
Supplement: Supporting Information [file supp_g3.113.005660_FileS1.pdf]

File S1

Supplementary materials and methods

Media

Cryptococcus strains were cultured in YPD (1% yeast extract, 2% Bacto-peptone, 2% glucose) and maintained at 4˚C on YPD solidified with 2% agar or stored at -80˚C in 15% glycerol. Phenotypic assays were conducted on YNB (yeast nitrogen base without amino acids and ammonium sulfate (BD, Franklin Lakes, NJ), 2% sugar or 10 mM carbon source, 10 mM nitrogen source, 2% agar). L-3,4-dihydroxyphenylalanine (L-DOPA) media with 10 mM nitrogen source for melanization assays was prepared as described (Chaskes and Tyndall 1975). Urease assays were conducted on Christensen's urea agar (Christensen 1946). Protease production was assayed using YNB with amino acids and ammonium sulfate supplemented with 2% glucose and 0.1% bovine serum albumin (Sigma-Aldrich, St Louis, MO). Phospholipase production was assayed on egg yolk agar as described (Chen *et al.* 1997). Capsule production was induced in liquid RPMI 1640 medium (Life Technologies, Carlsbad, CA) supplemented with 2% glucose and 10% foetal bovine serum (Life Technologies) as described (Zaragoza *et al.* 2003). *C. elegans* was maintained at 16ºC on nematode growth medium as described (Brenner 1974).

Phenotypic assays

Starter cultures were prepared by growth in YPD at 30ºC overnight with shaking, diluted to OD595 nm = 0.05 in water, then further diluted 10-fold in series. For growth, carbon utilization, melanization and stress assays, dilutions were spotted onto YPD, YNB supplemented with 2% sugar or 10 mM carbon source, L-DOPA or YNB supplemented with 5 mM caffeine, 0.25 mg/mL Congo red, 50 mg/mL calcofluor white, 0.01% SDS, 1 M sorbitol, 1 M NaCl, 0.25 mM H_2O_2 or 1 mM $NaNO_2$ as indicated. Images were taken following 2-3 days incubation at 30 (stress, phospholipase, protease, urease, nitrogen utilization), 30 and 37˚C (carbon utilization and melanization), or 30, 37 and 39˚C (growth). UV stress assay was conducted by exposing freshly spotted (YPD) cultures to 48 mJ/cm^2 UV light for 6, 12, 18 or 24 seconds in a UV Stratalinker (Stratagene). Capsule production was assayed at 30̊C with shaking and aliquots taken at 24 h. India ink (BD) staining was performed prior to visualization on a Zeiss Axioplan 2 epifluorescent/light microscope fitted with Axiocam greyscale camera running AxioVision AC software. Difference in capsule size was determined by calculating the ratio of capsule to cell diameter for each strain, measuring 50 cells per strain across 10 independent fields (Zaragoza *et al.* 2003). Statistical significance was determined using the unpaired, two-tailed *t*-test with *p* values <0.05 considered significant.

Fluconazole minimum inhibitory concentration determination

Minimum inhibitory concentration (MIC) assays were undertaken in biological triplicate following the broth microdilution protocol published by the Clinical and Laboratory Standards Institute (M27-A3) modified for *Cryptococcus* (Ghannoum *et al.* 1992). Fluconazole concentrations tested ranged from 0.1 to 50 µg/mL in two-fold dilutions. Plates were incubated at

35˚C for 72 h, scored visually at 24 h intervals and end-point reading taken electronically using a SpectraMax 250 (Molecular Devices). MIC_{90} (the lowest concentration of drug inhibiting ≥ 90% of growth) was recorded. *Candida albicans* (ATC90028)*, Candida krusei* (ATCC6258)*, Candida parapsilosis* (ATCC22019) and *C. neoformans* var. *grubii* (ATCC90113) were included as reference strains.

Read trimming and mapping

Quality was analyzed using FastQC (http://www.bioinformatics.bbsrc.ac.uk/projects/fastqc/). Reads were trimmed using CLC Genomics Workbench (4.6.1, CLC bio, Denmark) based on a phred-scaled quality score of 20 prior to mapping with this software. Post-trimming reads ≥ 30 nt were retained (77%, 78% and 77% respectively). BWA 0.5.9 (Li and Durbin 2009) was run on untrimmed reads. Reads were mapped using BWA and CLC Genomics Workbench using the unpublished, annotated H99 genome (www.broadinstitute.org/annotation/genome/cryptococcus_neoformans/MultiHome.html) as a reference. BWA was run with default settings. Marking of duplicate reads, realignment of reads around indels and recalibration of quality scores was then undertaken following the Genome Analysis Toolkit (GATK) pipeline (McKenna *et al.* 2010; DePristo *et al.* 2011). Recalibration was performed using a list of putative SNVs identified with GATK UnifiedGenotyper. Filters were applied as described (DePristo *et al.* 2011) with a required SNV frequency of 70% of reads. CLC Genomics Workbench was run using required read similarity of 95% and an insert size range of 130 to 300 bp. Other settings were left at default values.

***De novo* assembly**

SOAP *de novo* 1.05 (Li *et al.* 2008) and CLC Genomics Workbench were used to produce *de novo* assemblies using reads trimmed either with CLC Genomics Workbench as described above or with Trimmomatic (www.usadellab.org/cms/index.php?page=trimmomatic) using a quality cutoff of 20 across a sliding window of 4 nt, removing leading and trailing 3 nt from all reads and retaining post-trimming reads with length ≥ 30 nt. Assemblies were used as support for structural variation predictions. Separate *de novo* assemblies were run using unmapped reads in order to identity unique insertion sequences. Unmapped reads were extracted from BWA alignments using SAMtools 0.1.17 (Li *et al.* 2009) and Picard SamToFastq (picard.sourceforge.net). Assembled contigs were matched to the H99 genome using BLAST (Altschul *et al.* 1990). Potential insertion contigs from SOAP *de novo* were aligned to CLC Genomics Workbench contigs using BLAST to confirm the insert sequence.

Identification of repetitive regions

Transposable elements were identified using RepeatMasker (version open-3.3.0) (Smit *et al.* 1996-2010) with repeat library 20110419 utilizing the cross_match (version 0.990329) search engine (http://www.phrap.org/index.html). Additional

matches to known transposons of *C. neoformans* were identified via a BLAST search and annotated with blast2gffv3 (code.google.com/p/jperl/source/browse/trunk/scripts/Blast2Gff.pl?r=61). LTRHarvest (Ellinghaus *et al.* 2008) was used to predict LTR retrotransposons. Tandem repeats were identified using Tandem Repeats Finder (Benson 1999) and annotated using TRAP (Sobreira *et al.* 2006).

Genomic variation detection

Structural variation was detected using BreakDancer (Chen *et al.* 2009) (minimum mapping quality = 20, minimum supporting reads = 4), CREST (Wang *et al.* 2011) and Dindel (Albers *et al.* 2011). BreakDancer predictions with >90% confidence were examined manually. Dindel predictions present in both F0 and F2 were identified using BEDTools (Quinlan and Hall 2010). Dindel was also run on H99 mappings and F0 and F2 predictions overlapping with these were excluded. Unique predictions in F0 and F2 were examined manually. Validation was performed using primers listed (Table S4). Identification of synteny was performed using Mauve (Pareek *et al.* 2011). SNVs were identified using GATK UnifiedGenotyper with filters as described and CLC Genomics Workbench with default settings. Centromere regions were excluded from analysis. Comparisons between strains were performed using BEDtools (Quinlan and Hall 2010) and CLC Genomics Gateway beta. Figure 4 was constructed using Circos (Krzywinski *et al.* 2009). Copy number variation was detected using FREEC (Boeva *et al.* 2011) employing a 1,001 bp windows with a 100 bp step size and using H99 as control. Altered copy regions were then manually examined in Integrative Genomics Viewer 1.5 (Robinson *et al.* 2011), filtering out regions identified within telomeres and centromeres due to their repetitive nature. Coverage at the chromosome and gene level was determined using GATK DepthOfCoverage and CLC Genomics Workbench. rDNA and mitochondrial genome copy number was calculated as described (James *et al.* 2009).

References

Albers, C. A., G. Lunter, D. G. MacArthur, G. McVean, W. H. Ouwehand *et al.*, 2011 Dindel: Accurate indel calls from short-read data. Genome Res. **21:** 961-973.

Altschul, S. F., W. Gish, W. Miller, E. W. Myers, and D. J. Lipman, 1990 Basic local alignment search tool. J. Mol. Biol. **215:** 403-410.

Benson, G., 1999 Tandem repeats finder: a program to analyze DNA sequences. Nucleic Acids Res. **27:** 573-580.

Boeva, V., A. Zinovyev, K. Bleakley, J. P. Vert, I. Janoueix-Lerosey *et al.*, 2011 Control-free calling of copy number alterations in deep-sequencing data using GC-content normalization. Bioinformatics **27:** 268-269.

Brenner, S., 1974 Genetics of *Caenorhabditis elegans.* Genetics **77:** 71-94.

Chaskes, S., and R. L. Tyndall, 1975 Pigment production of *Cryptococcus neoformans* from *para*-diphenols and *ortho*-diphenols: Effect of nitrogen source. J. Clin. Microbiol. **1:** 509-514.

Chen, K., J. W. Wallis, M. D. McLellan, D. E. Larson, J. M. Kalicki *et al.*, 2009 BreakDancer: an algorithm for high-resolution mapping of genomic structural variation. Nat Meth **6:** 677-681.

Chen, S. C. A., M. Muller, J. Z. Zhou, L. C. Wright, and T. C. Sorrell, 1997 Phospholipase activity in *Cryptococcus neoformans*: A new virulence factor? J. Infect. Dis. **175:** 414-420.

Christensen, W. B., 1946 Urea decomposition as a means of differentiating *Proteus* and paracolon cultures from each other and from *Salmonella* and *Shigella* types. J. Bacteriol. **52:** 461-466.

DePristo, M., E. Banks, R. Poplin, K. Garimella, J. Maguire *et al.*, 2011 A framework for variation discovery and genotyping using next-generation DNA sequencing data. Nat. Genet. **43:** 491-498.

Ellinghaus, D., S. Kurtz, and U. Willhoeft, 2008 LTRharvest, an efficient and flexible software for de novo detection of LTR retrotransposons. BMC Bioinformatics **9:** 18. doi: 10.1186/1471-2105-9-18.

Ghannoum, M. A., A. S. Ibrahim, Y. Fu, M. C. Shafiq, J. E. Edwards *et al.*, 1992 Susceptibility testing of *Cryptococcus neoformans*: A microdilution technique. J. Clin. Microbiol. **30:** 2881-2886.

James, S. A., M. J. T. O'Kelly, D. M. Carter, R. P. Davey, A. van Oudenaarden *et al.*, 2009 Repetitive sequence variation and dynamics in the ribosomal DNA array of *Saccharomyces cerevisiae* as revealed by whole-genome resequencing. Genome Res. **19:** 626-635.

Krzywinski, M., J. Schein, I. Birol, J. Connors, R. Gascoyne *et al.*, 2009 Circos: An information aesthetic for comparative genomics. Genome Res. **19:** 1639-1645.

Li, H., and R. Durbin, 2009 Fast and accurate short read alignment with Burrows-Wheeler transform. Bioinformatics **25:** 1754-1760.

Li, H., B. Handsaker, A. Wysoker, T. Fennell, J. Ruan *et al.*, 2009 The Sequence Alignment/Map format and SAMtools. Bioinformatics **25:** 2078-2079.

Li, R., Y. Li, K. Kristiansen, and J. Wang, 2008 SOAP: short oligonucleotide alignment program. Bioinformatics **24:** 713-714.

McKenna, A., M. Hanna, E. Banks, A. Sivachenko, K. Cibulskis *et al.*, 2010 The Genome Analysis Toolkit: a MapReduce framework for analyzing next-generation DNA sequencing data. Genome Res. **20:** 1297-1303.

Pareek, C. S., R. Smoczynski, and A. Tretyn, 2011 Sequencing technologies and genome sequencing. J. Appl. Genetics **52:** 413-435.

Quinlan, A. R., and I. M. Hall, 2010 BEDTools: A flexible suite of utilities for comparing genomic features. Bioinformatics **26:** 841-842.

Robinson, J. T., H. Thorvaldsdottir, W. Winckler, M. Guttman, E. S. Lander *et al.*, 2011 Integrative genomics viewer. Nat. Biotechnol. **29:** 24-26.

Smit, A. F. A., R. Hubley, and P. Green, 1996-2010 RepeatMasker Open-3.0.

Sobreira, T. J. P., A. M. Durham, and A. Gruber, 2006 TRAP: automated classification, quantification and annotation of tandemly repeated sequences. Bioinformatics **22:** 361-362.

Wang, J. M., C. G. Mullighan, J. Easton, S. Roberts, S. L. Heatley *et al.*, 2011 CREST maps somatic structural variation in cancer genomes with base-pair resolution. Nat. Methods **8:** 652-654.

Zaragoza, O., B. C. Fries, and A. Casadevall, 2003 Induction of capsule growth in *Cryptococcus neoformans* by mammalian serum and CO_2. Infect. Immun. **71:** 6155-6164.
