# Supplementary material for: Comparative Genomics of Serial Isolates of Cryptococcus neoformans Reveals Gene Associated With Carbon Utilization and Virulence
Source: G3 (Bethesda). 2013 Apr 1;3(4):675–86. doi: 10.1534/g3.113.005660 (PMC3618354; doi:10.1534/g3.113.005660)
Supplement: Supporting Information [file supp_g3.113.005660_TableS1.pdf]

Table S1A Indels identified between H99 and F0 and F2 within genes with functional annotation

Chr	H99	F0 & F2	Gene	Gene function	Effect of mutation on protein
**1**	T	TCCCACA	CNAG_00545	IDN3-B	Insertion of PH in 5x string at 259 (of 1,933)
**1**	T	TGGTTCA	CNAG_00619	tubulin folding cofactor C	Insertion of EP in 4x string at 224 (of 356)
**3**	GTTC	G	CNAG_02824	uncharacterized ACR, COG1565	Deletion of R11 (of 543)
**3**	GCAT	G	CNAG_02796	phospho-2-dehydro-3-deoxyheptonate aldolase	Deletion of S in 7x string at 281 (of 544)
**3**	AGA	AC	CNAG_07527	α-1,6-mannosyl transferase	H232E, truncation at 250 (of 518)
**3**	A	AAGAAGC	CNAG_07580	CAMK/CAMKL/MARK protein kinase	Insertion of AS in 3x string at 513 (of 1,172)
**3**	GATCGTC	G	CNAG_06888	cytoplasmic protein	Deletion of SS in 5x string at 414 (1,037)
**3**	AG	A	CNAG_06888	cytoplasmic protein	G1024A (of 1,037)
**3**	A	ACAAGCC	CNAG_06920	ubiquitin-specific protease	Insertion of AQ in 3x string at 276 (of 1,109)
**3**	GAGA	G	CNAG_06920	ubiquitin-specific protease	Deletion of K501 (of 1,109)
**3**	C	CCTT	CNAG_06927	peptidyl-prolyl cis-trans isomerase	Insertion of E480 (of 513)
**4**	A	ATGATGAAG G	CNAG_05096	histone deacetylase 3	Insertion of SSP151 (of 490)
**4**	G	GGTT	CNAG_05138	exo-β-1,3-glucanase	Insertion of N37 (of 785)
**4**	C	CCAGAAA	CNAG_05248	clathrin binding protein	Insertion of KQ738 (of 751)
**4**	G	GT	CNAG_05274	STE/STE20/YSK protein kinase	G727V, truncation at 727 (of 765)
**5**	TA	T	CNAG_00974	beta-lactamase domain-containing protein	C188Stop (of 300)
**7**	G	GATGGA	CNAG_06533	ATP-dependent permease	Insertion of NGNG in 2x string at 648 (of 1,051)
**7**	AAAGAGACA TACGT	A	CNAG_06556	oxidoreductase	Q242R, truncation at 260 (of 323)
**7**	TC	T	CNAG_06623	*myo*-inositol oxygenase	S124L, truncation at 151 (of 315)
**8**	TCCCGCCACC	T	CNAG_03189	DIL and ankyrin domain-containing protein	G1093D (of 1,105)
**8**	TGTC	T	CNAG_03321	vacuolar protein sorting-associated protein 9	Deletion of T in 7x string at 131 (of 714)
**8**	TG	T	CNAG_03339	biotin transporter	H439P, truncation at 458 (of 503)
**8**	AG	ATA	CNAG_03519	cytoplasmic protein	Q453H, truncation at 459 (of 502)

Chr	H99	F0 & F2	Gene	Gene function	Effect of mutation on protein
**8**	TC	T	CNAG_03533	JmjC domain-containing histone demethylation protein 1	A505G, truncation at 557 (of 874)
**9**	A	AACC	CNAG_04213	signal transducer	Insertion of G152 (of 978)
**9**	A	AAGC	CNAG_04380	peptidase	Insertion of S in 6x string at 250 (of 688)
**10**	T	TAGA	CNAG_04792	phosphatidyl serine decarboxylase	Insertion of E in 4x string at 340 (of 1,230)
**10**	G	GA	CNAG_04696	DNA clamp loader	S438F, truncation at 444 (of 760)
**11**	A	ACGGTGATG G	CNAG_01809	small nuclear ribonucleoprotein hPrp3	Insertion of GGD501 (of 590)
**11**	TCAACTCCAA CTC	T	CNAG_02000	short-chain dehydrogenase	Deletion of SNSN in 3x string at 40 (of 358)
**11**	AG	A	CNAG_02016	DUF1479 domain-containing protein	V158L, truncation at 196 (of 493)
**12**	ACAG	A	CNAG_06193	CMGC/RCK protein kinase	Deletion of Q in 5x string at 94 (of 1,262)
**13**	A	AC	CNAG_06499	phosphatidic acid phosphatase type 2 domain containing 1 protein	C252V, truncation at 297 (of 382)

Table S1B Structural variation identified between H99 and F0 and F2

Chr	Type	Size (bp)	Gene	Gene name
**1**	Deletion	3,407	CNAG_00549	hypothetical protein
**2**	Deletion	47	CNAG_03991	integral membrane protein
**2**	Insertion	656	CNAG_04003 (intron)	pumilio 2
**3**	Deletion	383		
**3**	Deletion	3,409	CNAG_02711	hypothetical protein
**3**	Deletion	150	CNAG_02706, CNAG_02707	hypothetical proteins
**4**	Deletion	539		
**4**	Deletion	557	CNAG_05266 (intron)	membrane protein
**5**	Deletion	302	CNAG_06863	hypothetical protein
**5**	Deletion	716	CNAG_06843	hypothetical protein
**5**	Deletion	7,459	CNAG_01367, CNAG_01368	hypothetical proteins
**5**	Deletion	475		
**5**	Deletion	1,413	CNAG_01032	hypothetical protein
**6**	Deletion	260		
**6**	Insertion	664		
**7**	Tandem duplication	643	CNAG_06557	membrane protein
**7**	Deletion	323		
**7**	Insertion/ duplication	7,464	CNAG_00813/01368, CNAG_00814/01367	hypothetical proteins
**7**	Deletion	7,155	CNAG_05911, CNAG_05910	hypothetical proteins
**7**	Insertion/ duplication	9,600	CNAG_07313, CNAG_00127, CNAG_00128	hypothetical proteins
**8**	Insertion	1,033	CNAG_07707	glycoside hydrolase family 3 domain-containing protein
**8**	Insertion	915	CNAG_03195	hypothetical protein
**8**	Deletion	114		
**8**	Deletion	7,132	CNAG_03477, CNAG_03478	hypothetical proteins
**8**	Deletion	99		
**10**	Deletion	556	CNAG_07836 (intron)	NAD binding dehydrogenase family protein
**10**	Insertion	402		
**10**	Insertion	432	CNAG_04901	hypothetical protein
**10**	Insertion	664		
**10**	Deletion	5,210		
**10**	Deletion	246		
**11**	Deletion	7,427	CNAG_07613, CNAG_01968	hypothetical proteins
**13**	Deletion	77	CNAG_06336	glucan 1,3 beta-glucosidase
**13**	Deletion	15		
**14**	Deletion	591		

**14**	Deletion	165		
**14**	Deletion	248		
**14**	Deletion	85		
**14**	Deletion	592	CNAG_07874	sugar transporter
**14**	Deletion	11	CNAG_05643	hypothetical protein
