# Supplementary material for: Comparative Genomics of Serial Isolates of Cryptococcus neoformans Reveals Gene Associated With Carbon Utilization and Virulence
Source: G3 (Bethesda). 2013 Apr 1;3(4):675–86. doi: 10.1534/g3.113.005660 (PMC3618354; doi:10.1534/g3.113.005660)
Supplement: Supporting Information [file supp_g3.113.005660_TableS2.pdf]

Table S2 Genes on the left arm of chromosome 12

Strand	Locus	Length	Start	Stop	Name
-	CNAG_06939	1,938	204	2,141	conserved hypothetical protein
-	CNAG_06938	432	4,923	5,354	conserved hypothetical protein
-	CNAG_07026	670	7,207	7,876	conserved hypothetical protein
+	CNAG_07894	287	9,950	10,236	conserved hypothetical protein
-	CNAG_05986	1,052	12,447	13,498	conserved hypothetical protein
-	CNAG_05987	1,083	13,917	14,999	conserved hypothetical protein
-	CNAG_05988	580	15,532	16,111	conserved hypothetical protein
+	CNAG_05989	1,900	17,145	19,044	predicted protein
-	CNAG_05990	2,564	19,424	21,987	predicted protein
+	CNAG_05991	1,886	22,684	24,569	glycosyl hydrolase family 88
+	CNAG_05992	2,338	25,550	27,887	conserved hypothetical protein
-	CNAG_05993	2,668	27,970	30,637	transmembrane transporter Liz1
+	CNAG_05994	2,256	31,431	33,686	multidrug transporter
-	CNAG_05995	2,215	34,421	36,635	conserved hypothetical protein
+	CNAG_05996	3,075	38,318	41,392	amino acid transporter
-	CNAG_05997	849	42,493	43,341	conserved hypothetical protein
+	CNAG_05998	1,353	43,606	44,958	rho GTPase
-	CNAG_05999	974	45,350	46,323	peptide alpha-N-acetyltransferase
+	CNAG_06000	1,986	46,804	48,789	glycoprotein
+	CNAG_06001	1,923	49,240	51,162	phosphomevalonate kinase
-	CNAG_06002	1,752	51,476	53,227	tRNA (5-methylaminomethyl-2-thiouridylate)-methyltransferase
+	CNAG_06003	912	54,177	55,088	conserved hypothetical protein
-	CNAG_06004	1,440	55,430	56,869	conserved hypothetical protein
+	CNAG_06005	3,065	57,220	60,284	conserved hypothetical protein
-	CNAG_06006	1,542	60,590	62,131	conserved hypothetical protein
+	CNAG_06007	1,057	62,416	63,472	hypothetical protein
+	CNAG_06008	1,378	63,801	65,178	asparaginase
+	CNAG_06009	2,427	65,715	68,141	cyclohydrolase
-	CNAG_07895	402	70,290	70,691	predicted protein
-	CNAG_06010	2,541	71,423	73,963	fatty aldehyde dehydrogenase
+	CNAG_06011	2,097	74,927	77,023	conserved hypothetical protein
-	CNAG_06012	1,508	77,134	78,641	NADH-cytochrome b5 reductase
+	CNAG_06013	2,116	78,850	80,965	vacuolar protein
-	CNAG_06014	891	81,022	81,912	predicted protein
-	CNAG_06015	651	81,943	82,593	predicted protein
-	CNAG_07896	702	83,434	84,135	predicted protein
-	CNAG_06016	2,025	85,492	87,516	capsule associated protein 6
+	CNAG_06017	573	88,620	89,192	predicted protein
+	CNAG_06018	2,421	89,818	92,238	aldehyde dehydrogenase
+	CNAG_06019	5,653	93,866	99,518	conserved hypothetical protein

Strand	Locus	Length	Start	Stop	Name
-	CNAG_06020	1,738	100,066	101,803	conserved hypothetical protein
+	CNAG_06021	2,681	103,158	105,838	rab GTPase activator
-	CNAG_06022	1,429	105,986	107,414	trans-aconitate 3-methyltransferase
+	CNAG_06023	678	107,717	108,394	conserved hypothetical protein
-	CNAG_06024	1,294	108,487	109,780	peroxin 14
+	CNAG_06026	2,187	110,990	113,176	aspartate transaminase
-	CNAG_06027	2,064	112,960	115,023	aryl-alcohol dehydrogenase
-	CNAG_06028	1,242	116,243	117,484	conserved hypothetical protein
+	CNAG_06029	741	117,802	118,542	conserved hypothetical protein
-	CNAG_06030	1,968	118,609	120,576	conserved hypothetical protein
+	CNAG_06031	1,177	122,495	123,671	β-glucan synthesis-associated protein
+	CNAG_06032	948	124,300	125,247	ADP-ribosylation factor-like protein 2
-	CNAG_06033	1,661	125,368	127,028	pfkB family carbohydrate kinase superfamily
+	CNAG_06034	2,158	131,492	133,649	allantoin permease
-	CNAG_06035	1,814	134,163	135,976	alcohol dehydrogenase
+	CNAG_06036	2,651	136,817	139,467	aminotransferase LolT-1
