# Supplementary material for: Comparative Genomics of Serial Isolates of Cryptococcus neoformans Reveals Gene Associated With Carbon Utilization and Virulence
Source: G3 (Bethesda). 2013 Apr 1;3(4):675–86. doi: 10.1534/g3.113.005660 (PMC3618354; doi:10.1534/g3.113.005660)
Supplement: Supporting Information [file supp_g3.113.005660_TableS3.pdf]

Table S3 Statistical parameters of multivariate PCA models of metabolomic data

Model	N	k	A	R^2X	Q^2	Scaling	Comparison	Figure
**M1**	34	9151	6	0.919	0.85	Pareto	All samples	3A
**M2**	11	9151	3	0.876	0.698	Pareto	F0 *vs.* F2 in YNB medium	3B & C
**M3**	11	9151	2	0.936	0.889	Centre	F0 *vs.* F2 in YNB medium	3B & C
**M4**	12	9151	3	0.785	0.458	Pareto	F0 *vs.* F2 in YPD medium	S5A & B
**M5**	12	9151	5	0.98	0.866	Centre	F0 *vs.* F2 in YPD medium	S5A & B
**M6**	12	9151	4	0.916	0.826	Pareto	F2 in YNB *vs.* YPD medium	S5C & D
**M7**	12	9151	3	0.986	0.961	Centre	F2 in YNB *vs.* YPD medium	S5C & D
**M8**	11	9151	3	0.848	0.545	Pareto	F0 in YNB *vs.* YPD medium	S5E & F
**M9**	11	9151	2	0.838	0.567	Centre	F0 in YNB *vs.* YPD medium	S5E & F

N= number of samples, k= number of x-variables (buckets), A= number of principal components in the model, R^2X=sum of squares of all x-variables explained by the model, Q^2= cumulative cross-validated R^2.
