# Supplementary material for: Comparative Genomics of Serial Isolates of Cryptococcus neoformans Reveals Gene Associated With Carbon Utilization and Virulence
Source: G3 (Bethesda). 2013 Apr 1;3(4):675–86. doi: 10.1534/g3.113.005660 (PMC3618354; doi:10.1534/g3.113.005660)
Supplement: Supporting Information [file supp_g3.113.005660_TableS4.pdf]

Table S4 Primers used in this study

Primer ID	Sequence
**UQ623**	CAGGTCAAGGAAAGGCAACAG
**UQ624**	TGCCCTGAAGTTGGTTAGACT
**UQ702**	ACAAAATCCACGCATACAGAA
**UQ703**	CGCCACCATCCAAACGTCAAA
**UQ704**	CAATCCCTTTGCCATCTGAAC
**UQ705**	GGTCAACCGCCTGTTTCTAAT
**UQ706**	CCCATATTATTCCCAATCTGA
**UQ707**	CCTGCATTGTTCGCCTCTCTA
**UQ708**	GCGTGGCCGACCTTACCTCAG
**UQ709**	CCAATACACCAACAGCGTGAC
**UQ801**	GCGAAAGGGTGAGAAGTGAAA
**UQ802**	CACTAAAGGGCGGCCATTAAA
**UQ1836**	AGCTCTACCCACTACCGAATC
**UQ1837**	CGACGATGACTCCCACTACCA
**UQ1838**	CACCTGGAGAAGTTCGCTGAG
**UQ1839**	GGCTGAACATTGTCGCTTATT
**UQ1840**	TGTGGGATGAATGTAAGTGCT
**UQ1842**	GGACTTTGCTTCGGATGATCT
**UQ1843**	CACATGCTCGCTTAGTTGC
**UQ1844**	TATCAGCGAAATACGACAAGG
**UQ1845**	GCTAGTCACAGGTCGTCGGT
**UQ1846**	GCACTTCCAACGCAGGTT
**UQ1847**	CGCTCTTGCTTTGACCAACTC
**UQ1848**	TTGCAATGACAAAAGGCTTAT
**UQ1849**	TGCTCTCCACCACTTCGATT
**UQ1850**	CGGCAGGTTCGATATGG
**UQ1851**	TGTTGTTGTTGCGTAGTCGTC
**UQ1852**	CAGGCTAGTGAGTCGGCTACA
**UQ1853**	CGGGGAATTCGATCATC
**UQ1854**	CTCCTCGCACGGTTCTC
**UQ1855**	TGGAGGCTCGTGACATATGAA
**UQ1856**	AGCCATTTCTTTCAGTCGAGC
**UQ1857**	GGGGTTTGATTGGTGCAAG
**UQ1858**	CCTCCGTCCTCCGAGTCGTT
**UQ1859**	GGAAGAAATTGGGTAAGCC
**UQ1860**	AAACATGTCTACCGTTGACCC
**UQ1861**	CGGAGGTGAAATAGAACGC
**UQ1862**	CCCTTATCCAAGATTCCGTGA
**UQ1865**	CGGCATAGAGAACGGGAAG
**UQ1866**	ACGCTTTCAGACCCTCGTTCT
**UQ1869**	TCTTGAATCTGCGAGCGTGAA
**UQ1870**	CACCGAAAGAAGCGTCTAAAC
**UQ1873**	CTTCTCCATCCATGCTGACA
**UQ1874**	CGGCCTCATGATGTTAAGT
**UQ1877**	CGGCATAGAGAACGGGAAG
**UQ1878**	GTACCGTAGTGCGCCTGA
**UQ1881**	AGCCGACGACGAAGTTCA
**UQ1882**	CACAAAGACCTTGCCATTATG
**UQ1885**	CAAAAAAAGATTCTCCCTCC
**UQ1886**	CCCTGAACCAGCCGTCT
**UQ1889**	GTATTGGAGGCACGGCAGA

Primer ID	Sequence
**UQ1894**	GGCGATGGTAAAGATGATAGC
**UQ1897**	CTCTCAAGAAGACGCTGACTT
**UQ1898**	GGCCTGGTGTCGGTATTC
**UQ1899**	CGCATCCTTCAATGACATTGG
**UQ1902**	GTCATCCGCAACTCATATCA
**UQ1903**	TGCTGTAGCGTCTGCGTGTGA
**UQ1904**	TCAAGCGAACTTAAAGGGTAA
**UQ1905**	AGGCGTTGGACTTCGAC
**UQ1906**	CCATACTTTTTGCCCGTTTAC
**UQ1907**	GGCCCAGGAGGTGATCAATTT
**UQ1908**	CGCGCCTTTCCTATCTC
**UQ1909**	GATGTGGGAATGACGGGAGTC
**UQ1910**	GAAATGTTCCCGTGTCGTCAT
**UQ1911**	CGAAATTGTTTGCCGATTG
**UQ1986**	GGAGGTGGACTGTATTGTGAG
**UQ1987**	CAATAATATCGTCTCGGGTGG
**UQ1990**	CGATGGATTTTAACCGTGACT
**UQ1991**	CCCGTATTTCAAACACTCTCA
**UQ1994**	CAGGGCACGAAAGGGACAGGT
**UQ1995**	GGGAAGTGATTGAGCATTTTT
**UQ1998**	CGACGGCTGAAGAACAAGGTG
**UQ1999**	ATCCGTAATCATTGCCAACAC
**UQ2000**	GCTCGCACTTCACCACTATTC
**UQ2001**	GTTGTGCTGGCTGCTGTTGTT
**UQ2004**	GCCCAAGGAAGGAAAGCTCAA
**UQ2005**	GGCTTGAGGCAAAAGACGACA
**UQ2006**	GGTGGACGAGAAGGATGAAAA
**UQ2007**	CGTCAGACCTAGCGTTTACTT
**UQ2008**	CTGCCAGTTATGAGCTGTCGG
**UQ2009**	TGCTTGTATGGTTGTCGCATT
**UQ2010**	TGGGGGACAGGGATGCACGGA
**UQ2011**	AAAGGCAAGTCACGTCGAAAA
**UQ2012**	AAGAAGTCGATCTCCCGCTCA
**UQ2013**	TAGGCTTCACTGACACACAAA
**UQ2014**	ATAAAAACCAGCCAGGTCTGC
**UQ2015**	CCTACTTCTTGTAGTAGCTGG
**UQ2016**	ACAGGGAAATGCTAAAAGTAT
**UQ2017**	AAGTTCGGGTACTCTGCTCTC
**UQ2018**	CACTTACTCGCCAGTCCACCA
**UQ2023**	CAGTGAGCCATCCTTACAGTC
**UQ2024**	AAACGATGATGCTGGAATTAC
**UQ2025**	CTGAGCATAATAATGGCGTCC
**UQ2026**	GAGATAAGGTCATCGCAAAAC
**UQ2072**	TGTACGAAGGCTATGAAGCTG
**UQ2073**	GATGGGCGTGTACTGTACTCT
**UQ2074**	TCACCGCTCGCTAAACCTGTT
**UQ2075**	CTAGGGTTTGGACGCACAACT
**UQ2076**	GGATACCAGCAATTCACTCCA
**UQ2087**	TCTCAGATCCTTCCCTTTGTC
**UQ2088**	CGCTCTCCAGCTCACATCCTCGCAGCTGAAACAGGATGT
**UQ2089**	CTACATCTCTTCCGTGTTAATACAGATAAACCGATGGTGGATGAGCTT
**UQ2090**	CTTTTTCGTTCAATCCTGCTT
**UQ2091**	ACATCCTGTTTCAGCTGCGAGGATGTGAGCTGGAGAGCG

Primer ID	Sequence
**UQ2092**	AAGCTCATCCACCATCGGTTTATCTGTATTAACACGGAAGAGATGTAG
**UQ2093**	CCGGAAGAGTGCTTGCAGATT
**UQ2094**	TTCGCCTCTGGACCGTGAATG
**UQ2095**	ACTTGAGCATTTGCGGGTGTG
**UQ2096**	TTTCCAGCACCAGCGTTCTTG
**UQ2097**	TGCTGACTGGGCCCTTGTCCT
**UQ2098**	GTGCGCATGAATTCGTGGACT
**UQ2099**	CAAACCCTTTCCCGGACGACT
**UQ2156**	CTAGGAATGAACATGGGAATG
**UQ2157**	TAGTTTGTAGGTTGACCGGTT
**UQ2158**	AGTGCACGAGGTTATTGAAGA
**UQ2159**	CAGAAAGATCGAGAGAAACAG
**UQ2160**	TAAATCTTGGTCGTTGGGGTC
**UQ2263**	AAATGCAGCGGAACCAAAATC
**UQ2264**	AGAGCTAAGTTAAATGGGGGG
